# Disruption of Aryl Hydrocarbon Receptor Homeostatic Levels during Embryonic Stem Cell Differentiation Alters Expression of Homeobox Transcription Factors that Control Cardiomyogenesis

**DOI:** 10.1289/ehp.1307297

**Published:** 2013-09-20

**Authors:** Qin Wang, Jing Chen, Chia-I Ko, Yunxia Fan, Vinicius Carreira, Yinglei Chen, Ying Xia, Mario Medvedovic, Alvaro Puga

**Affiliations:** Center for Environmental Genetics, Department of Environmental Health, University of Cincinnati College of Medicine, Cincinnati, Ohio, USA

## Abstract

Background: The aryl hydrocarbon receptor (AHR) is a ligand-activated transcription factor that regulates the expression of xenobiotic detoxification genes and is a critical mediator of gene–environment interactions. Many AHR target genes identified by genome-wide gene expression profiling have morphogenetic functions, suggesting that AHR may play a role in embryonic development.

Objectives: To characterize the developmental functions of the AHR, we studied the consequences of AHR activation by the agonist 2,3,7,8-tetrachlorodibenzo-*p*-doxin (TCDD), and the result of its repression by the antagonists 6,2,4-trimethoxyflavone and CH 223191 or by short-hairpin RNA (shRNA)-mediated *Ahr* knockdown during spontaneous differentiation of embryonic stem (ES) cells into cardiomyocytes.

Methods: We generated an AHR-positive cardiomyocyte lineage differentiated from mouse ES cells that expresses puromycin resistance and enhanced green fluorescent protein (eGFP) under the control of the Cyp1a1 (cytochrome P450 1a1) promoter. We used RNA sequencing (RNA.Seq) to analyze temporal trajectories of TCDD-dependent global gene expression in these cells during differentiation.

Results: Activation, inhibition, and knockdown of *Ahr* significantly inhibited the formation of contractile cardiomyocyte nodes. Global expression analysis of AHR-positive cells showed that activation of the AHR/TCDD axis disrupted the concerted expression of genes that regulate multiple signaling pathways involved in cardiac and neural morphogenesis and differentiation, including dozens of genes encoding homeobox transcription factors and Polycomb and trithorax group proteins.

Conclusions: Disruption of AHR expression levels resulted in gene expression changes that perturbed cardiomyocyte differentiation. The main function of the AHR during development appears to be the coordination of a complex regulatory network responsible for attainment and maintenance of cardiovascular homeostasis.

Citation: Wang Q, Chen J, Ko CI, Fan Y, Carreira V, Chen Y, Xia Y, Medvedovic M, Puga A. 2013. Disruption of aryl hydrocarbon receptor homeostatic levels during embryonic stem cell differentiation alters expression of homeobox transcription factors that control cardiomyogenesis. Environ Health Perspect 121:1334–1343; http://dx.doi.org/10.1289/ehp.1307297

## Introduction

The theory of the developmental origins of adult disease proposes that the environment encountered during fetal life and infancy permanently changes the body’s structure, function, and metabolism and shapes the long-term control of tissue physiology and homeostasis (Barker 2007). Accordingly, damage during fetal life or infancy resulting from maternal stress, poor nutrition, or exposure to environmental pollutants such as dioxin may be at the heart of adult-onset disease. Work in many laboratories has shown that the young are more sensitive to dioxin than adults and that developmental exposure to TCDD (2,3,7,8-tetrachlorodibenzo-*p*-doxin)—the prototypical dioxin—results in disease conditions in adult fish (Plavicki et al. 2013), birds (Walker and Catron 2000), and mammals (Kopf and Walker 2009). Bruner-Tran and Osteen (2011) reported that dioxin exposure reduced fertility and negatively affected pregnancy outcomes across multiple generations. The developmental toxicity of TCDD is of greater concern for humans because pregnant women transfer a fraction of their dioxin body burden to the fetus during pregnancy and to the infant via breastfeeding (Schecter et al. 2001). In addition, dioxin-like organochlorinated compounds are epidemiologically associated with low birth weight and respiratory distress (Lai et al. 2002) as well as cardiac malformations (Dummer et al. 2003). In their study, Dummer et al. (2003) reported that infants born to mothers living near incinerators that emitted complex mixtures of dioxins, furans, particulates, and heavy metals exhibited a higher incidence of lethal congenital heart diseases. Other studies have shown an epidemiological association between the incidence of hypoplastic left heart syndrome and maternal exposure to halogenated hydrocarbons, dioxins, and polychlorinated biphenyls (PCBs) during pregnancy (Kuehl and Loffredo 2006).

Most biological effects of TCDD are mediated by the aryl hydrocarbon receptor (AHR), a ligand-activated transcription factor and a member of the basic-region-helix-loop-helix PER/ARNT/SIM (Bhlh-PAS) superfamily of transcription factors. Members of this superfamily function as sensors of extracellular signals and environmental stresses that may affect growth and development (Gu et al. 2000). Activation by TCDD causes receptor translocation to the nucleus, dissociation from its cytosolic chaperones, and heterodimerization with its AHR nuclear translocator (ARNT) partner, also a member of the bHLH/PAS superfamily (Reyes et al. 1992). Binding of AHR–ARNT complexes to AHR binding sites in the promoters of target genes *a*) recruits transcription cofactors and associated chromatin remodeling proteins, and *b*) signals initiation of gene transcription (Schnekenburger et al. 2007). Increasing evidence indicates that, in addition to the well-known xenobiotic metabolism genes in cytochrome P450, family 1 (Cyp1), there are other AHR transcriptional targets, including genes involved in cell-cycle regulation and morphogenetic processes, that may play a vital function during embryonic development (Sartor et al. 2009b). In this context, following a complex alternating pattern of activation and repression in the preimplantation mouse embryo (Wu et al. 2002), AHR expression can be demonstrated in the postimplantation embryo as early as gestation day (GD) 9.5, followed by widespread expansion into almost all developing organs including brain, heart, liver, somites, and branchial arches (Abbott et al. 1995).

The AHR is a major contributor to cardiovascular homeostasis in all species studied to date. In mice, fish, and avian embryos, the heart is a TCDD target during fetal development, which results in reduced cardiomyocyte proliferation, altered fetal heart size, and disruptions in neovascularization (Ivnitski-Steele and Walker 2005). *In utero* exposure to TCDD increases the susceptibility to cardiovascular dysfunction in adult life (Aragon et al. 2008). Consistent with the concept that the AHR is a major player in cardiac function, knockout of the *Ahr* gene in mice disrupts cardiovascular homeostasis, causing pathological cardiac hypertrophy (Lund et al. 2003).

To address the hypothesis that AHR activation by TCDD during embryonic development disrupts expression of genes critical to cardiac differentiation, we generated an AHR-positive embryonic stem cell lineage that expresses puromycin resistance and enhanced green fluorescent protein (eGFP) under the control of the AHR-responsive *Cyp1a1* promoter. Activation of the AHR/TCDD axis in these cells disrupts the concerted expression of genes that regulate multiple signaling pathways involved in cardiac and neural morphogenesis and differentiation, including dozens of genes encoding homeobox transcription factors and Polycomb and trithorax group genes. Our functional analysis of those genes suggests that homeostatic levels of AHR establish a complex regulatory network that controls various aspects of embryonic development, including cardiomyocyte differentiation.

## Materials and Methods

*Animals and TCDD exposure*. C57BL/6J mice were housed in the Experimental Laboratory Animals Medical Services at the University of Cincinnati under controlled conditions of temperature, humidity, and lighting, and were provided standard mouse chow and water *ad libitum*. All experimental procedures conducted with these animals have been approved by the University of Cincinnati Animal Care and Use Committee. Animals were treated humanely and with regard for alleviation of suffering.

Female mice were mated overnight; on GD5.5 pregnant dams were treated by oral gavage with either corn oil vehicle or with TCDD (5 or 50 μg/kg) in corn oil. Based on previous determinations using isotopically labeled TCDD, these doses to the pregnant dam correspond to doses of 1.7 ng/kg and 17 ng/kg, respectively, to the embryos (Weber and Birnbaum 1985). For analysis of AHR expression, the uteri of pregnant dams were harvested on GD7.5 and prepared for immunofluorescence detection.

*Culture of embryonic stem (ES) cells,* in vitro *differentiation, and treatments.* ES cell culture and differentiation. Undifferentiated C57BL/6N-C2 ES cells (Gertsenstein et al. 2010) were maintained in ES medium consisting of high-glucose Dulbecco’s minimal essential medium (DMEM; Gibco; Carlsbad, CA) supplemented with 15% ES cell qualified serum (Knockout Serum Replacement; Gibco) 2 mM glutamine, 1% nonessential amino acids, 100 U/mL penicillin, 100 μg/​mL streptomycin, 0.1 mM β-mercaptoethanol, and 1,000 U/mL ESGRO leukemia inhibitory factor (LIF; Bioscience Research Reagents, Temecula, CA). Cells were seeded in 0.1% gelatin-coated plates, incubated at 37°C (95% humidity, 5% CO_2_) and passaged every second or third day. Cell differentiation was initiated on day 0 by first forming embryoid bodies (EBs) in hanging drops. Cells were transferred to LIF-free DMEM supplemented with 15% non-ES–qualified fetal bovine serum and suspended at a concentration of 40,000–70,000 cells/mL. Sixty 20-μL aliquots were pipetted onto the inner surface of a bacterial Petri dish lid, and the lid was inverted over the bottom plate containing 15 mL phosphate-buffered saline (PBS) to provide humidity. Plates were incubated at 37°C for 3 days, after which the EBs were flushed with differentiation medium and incubated in 24-well or 10-cm plates for varying periods of time.

EB treatment. Cultured EBs were treated for various lengths of time with TCDD at concentrations of 10 pM to 1 nM (doses commonly used for tissue culture studies of the high-affinity AHR of C57BL/6 mice). TCDD was dissolved in DMSO and diluted in DMEM to reach the desired concentration. DMSO in DMEM served as the vehicle control. For both TCDD and vehicle control, the final concentration of DMSO was ≤ 0.05% of the final volume.

Cardiomyocyte contractility. EBs were individually plated on wells of 24-well plates, allowed to differentiate in the presence of the indicated concentrations of TCDD or vehicle, and visually scored daily for the presence of beating cell (cardiomyocyte) clusters. Beating became evident starting on days 6–7 and became maximal by days 10–11. If a well contained more than one beating cluster, it was scored as a single beating EB. Beating and nonbeating areas of differentiated EBs were manually dissected under a dissecting microscope. In some experiments, differentiating cells were treated with varying concentrations of the AHR antagonists 6,2,4-trimethoxyflavone (TMF; Indofine Chemical Co., Hillsborough, NJ) or CH 223191 (Chembridge, San Diego, CA) to study the role of AHR activation suppression in the beating phenotype.

*Short-hairpin RNA (shRNA) knockdown of* Ahr *expression*. We purchased the validated lentiviral shRNA construct targeting *Ahr* transcripts TRCN0000055410 from the Mission ShRNA Lentiviral Collection (Sigma-Aldrich, St. Louis, MO) and a nonsilencing control construct from the Lentivirus-shRNA Library Core of the Cincinnati Children’s Hospital Medical Center (Cincinnati, OH). Mouse pluripotent ES cells were infected with these viruses in the presence of 8 μg/​mL polybrene, and stable *Ahr*-knockdown ES cells were selected for resistance to 3 μg/mL of puromycin. The efficiency of knockdown was determined by immunoblotting.

*Preparation of whole-cell extracts for immunoblotting*. Cells were washed and harvested in PBS containing 1× complete protease inhibitor and lysed in 300 μL NETN (100 mM NaCl, 20 mM Tris, pH 8.0, 1 mM EDTA, 0.5% NP-40, and 1× complete protease inhibitor). After lysis, cells were sonicated on ice three times for 10 sec each with a Fisher Scientific Sonic Dismembrator 60. Protein concentrations were measured using the Pierce BCA protein assay (Thermo Scientific, Florence, KY). Protein extract aliquots (50 μg) were analyzed by SDS-polyacrylamide gel electrophoresis, transferred to polyvinylidene fluoride membranes, and probed for AHR (Enzo Life Sciences Inc., Farmingdale, NY) and β-actin (Sigma-Aldrich).

*Immunofluorescence*. For immunofluorescence studies, cells were seeded on 10-mm glass coverslips, fixed with 4% paraformaldehyde in PBS for 20 min, permeabilized with 0.1% triton X-100 for 20 min, blocked with 5% bovine serum albumin (BSA) for 0.5 hr, and incubated with the primary antibody at 4°C overnight. After washing, coverslips were stained with Alexa 488– or Alexa 567–labeled secondary antibodies and Hoechst solution. We examined the cells and captured the images using a Zeiss Axio microscope (Carl Zeiss Microscopy, Thornwood, NY). At least five fields were evaluated for each treatment group.

For the analysis of AHR localization by immunofluorescence, 3-day-old EBs were collected by low-speed centrifugation and rinsed once with PBS; the pellet was fixed in 4% paraformaldehyde (Sigma-Aldrich) and embedded in HistoGel (Thermo Scientific). For analysis, we used 5-μm sections and the following antibodies: GATA4 (Santa Cruz Biotechnology, Santa Cruz, CA), cardiomyocytes (MF20; Developmental Studies Hybridoma Bank at the University of Iowa, Iowa City, IA), keratin-18 (Thermo Scientific), AHR (Enzo Life Sciences Inc.), cardiac troponin T (Thermo Scientific), SHOX2 (Santa Cruz Biotechnology), and NKX2-5 (Santa Cruz Biotechnology).

Gravid uteri were removed on GD7.5, rinsed in PBS, adhered to white filter paper for proper orientation, and fixed in 4% paraformaldehyde overnight. Following fixation, uterine horns were dissected transversally to include implantation sites. Samples were then processed for histopathology; that is, tissue was dehydrated, clarified, embedded in paraffin (with embryos oriented longitudinally to the plane of section), cut into 5-μm sections, and placed on slides. Deparaffinized and rehydrated tissue sections were boiled in 10 mM citrate, pH 6, for 10 min and allowed to cool to room temperature. After blocking tissue sections in 5% BSA in PBS, pH 7.4, for 30 min at room temperature, the sections were incubated overnight with primary antibody at 4°C, washed three times with PBS, incubated for 1 hr at room temperature with the appropriate fluorescent-labeled secondary antibody (Invitrogen, Carlsbad, CA) diluted in 5% BSA in PBS. Sections were washed again and a coverslip was affixed with mounting medium containing DAPI (4´,6-diamidino-2-phenylindole). Micrographs were taken using a Zeiss Axio Scope.A1 microscope equipped with an AxioCam ICm1 and Zeiss Zen software (all from Carl Zeiss Microscopy).

*Total RNA isolation, reverse transcription, and real-time reverse transcription polymerase chain reaction (RT-PCR)*. Total RNA was extracted with the RNeasy Mini Kit (Qiagen, Valencia, CA) according to the manufacturer’s specifications. First-strand complementary DNAs (cDNAs) were synthesized from 10 μg of total RNA in a volume of 15 μL containing 1× reverse transcriptase buffer, 7 mM random hexamers primer, 0.5 mM dNTP mix, 10 mM dithiothreitol, 5 mM magnesium chloride, 20 U RNase inhibitor (RNasin; Promega, Madison, WI), and 100 U SuperScript III reverse transcriptase (Invitrogen). Samples were denatured and annealed to the primer for 10 min at 70°C and reverse transcribed for 3 hr at 42°C. Before amplification, the reverse transcriptase was inactivated by heating to 70°C for 15 min; RNA was hydrolyzed by incubation with 0.05 N sodium hydroxide at 70°C for 10 min and neutralized with 0.05 N hydrochloric acid (HCl); and the cDNA was precipitated with ethanol. The resulting cDNA products were dissolved in a final volume of 200 μL, and a 2-μL aliquot was used as template for subsequent quantification by real-time PCR amplification. PCR reactions were conducted in duplicate or triplicate in a total volume of 25 μL containing SYBR Green PCR Master Mix (Applied Biosystems, Grand Island, NY) and 0.1 μM of each primer. Primers for the genes tested [*Ahr, Cx40* (connexin 40), *Cyp1a1* (cytochrome P450 1A1), *Ece1* (endothelin converting enzyme 1), *Gata4* (GATA-binding protein 4), *Gata6* (GATA-binding protein 6), *Hcn4* (hyperpolarization-activated, cyclic nucleotide-gated K^+^ 4), *Kdr* (kinase insert domain protein receptor), *Mef2c* (myocyte enhancer factor 2C), *Mlc2v* (myosin, light polypeptide 2, regulatory, cardiac, slow), *Myh6* (myosin, heavy polypeptide 6, cardiac muscle, alpha), *Myh7* (myosin, heavy polypeptide 7, cardiac muscle, beta), *Nanog* (Nanog homeobox), *Nkx2-5* (NK2 homeobox 5), *Nppa* (natriuretic peptide type A; *Anf* ), *Oct4* (POU domain, class 5, transcription factor 1; *Pou5f1*), *Pgp9.5* (ubiquitin carboxy-terminal hydrolase L1; *Uchl1*), *Shox2* (short stature homeobox 2), *Tbx3* (T-box 3), and *Tbx5* (T-box 5)] are shown in Supplemental Material, Table S1.

Amplification was performed in an ABI 7500 real-time PCR system (Applied Biosystems); the reaction was heated to 95°C for 10 min, followed by 40 cycles of denaturation at 95°C for 15 sec and annealing elongation at 60°C for 60 sec. Detection of the fluorescent product was carried out during the 72°C extension period, and emission data were quantified using threshold cycle (C_t_) values. C_t_ values for all genes analyzed were determined in biological duplicates or triplicates, and means were determined from the average C_t_ values for each biological duplicate. All means were then normalized to values for *Gapdh* mRNA. PCR product specificity from each primer pair was confirmed using melting curve analysis and subsequent polyacrylamide gel electrophoresis.

*Selection of ES cells expressing AHR.* A 2-kb fragment of the mouse *Cyp1a1* promoter bearing the AHR-responsive enhancer and proximal promoter domains was inserted upstream of the puromycin resistance–eGFP gene complex in the PuroIRESeGFP vector for promoter sorting cell selection, a kind gift from A. Sachinidis and M. Jesudoss (Institute of Neurophysiology, University of Cologne, Cologne, Germany). This construct was transfected into the C2 ES cells using lipofectamine (Invitrogen) and used to generate an immortalized mouse ES cell line containing the stably integrated pAHRPuroIRESeGFP plasmid by selection with 600 μg/mL G418. Single colonies were picked up after selection and used for the analysis of AHR-dependent growth and differentiation by treatment with 100 pM TCDD for 4 hr, followed by removal of the drug and selection for 2–3 days in the presence of 3 μg/mL puromycin, during which time AHR-negative cells died because of their failure to activate the *Cyp1a1* promoter. As determined by RNA-seq analyses, this low-dose, short TCDD treatment was sufficient to activate the *Cyp1a1* promoter but did not result in the induction or repression of any other genes.

*RNA-seq data analysis*. All steps of library construction, cluster generation, and HiSeq (Illumina, San Diego, CA) sequencing were performed with biological duplicate samples by the Genomics Sequencing Core of the Department of Environmental Health, University of Cincinnati. Library construction was done with the TruSeq RNA sample preparation kit (Illumina) using 1 μg of total RNA, with RNA integrity number ≥ 7.0 (Agilent 2100 Bioanalyzer; Agilent Technologies, Santa Clara, CA) to purify poly-A–containing mRNA with oligo-dT–attached magnetic beads. The purified mRNA was enzymatically fragmented, with random hexamers primed for first and second strand cDNA synthesis, followed by purification using Agencourt AMPure XP beads (Beckman Coulter, Florence, KY). Overhangs in the double-strand cDNA were blunt-ended by end repair and adenylated with a single A-nucleotide at the 3´ end to prevent self-ligation in the following ligation step. AMPure XP bead-purified fragments were ligated to sample-specific indexing adapters and enriched by 10 cycles of PCR using adapter-specific primers. A 1-μL aliquot of purified PCR product (from a total sequencing library of 30 μL) was analyzed in an Agilent bioanalyzer using a DNA 1000 chip to check DNA size (~ 260 bp) and yield. To quantify the library concentration for clustering, the library was diluted 1:100 in a buffer containing 10 mM Tris-HCl, pH 8.0, and 0.05% Tween 20, and analyzed by quantitative PCR (qPCR) with a KAPA Library Quantification kit (KapaBiosystems, Woburn, MA) using an ABI 9700HT real-time PCR machine (Applied Biosystems). Equal amounts of six individually indexed cDNA libraries were pooled for clustering in an Illumina cBot system flow cell at a concentration of 8 pM using Illumina’s TruSeq SR Cluster Kit v3, and sequenced for 50 cycles using a TruSeq SBS kit on the Illumina HiSeq system. Each sample generated approximately 30 million sequence reads.

Sequence reads were demultiplexed and exported to fastq files using CASAVA 1.8 software (Illumina). The reads were then aligned to the reference genome (mm10) using TopHat aligner (Trapnell et al. 2009). The counts of reads aligning to each gene’s coding region were summarized using ShortRead (Morgan et al. 2009) and associated Bioconductor packages (GenomicFeatures, IRanges, GenomicRanges, Biostrings, Rsamtools; http://www.bioconductor.org/packages/release/BiocViews.html#___Software) for manipulating and analysis of next-generation sequencing data and custom-written R programs (Ihaka and Gentleman 1996). Differential gene expression analysis between AHR-positive and unselected cells was performed separately at each of the four different time points (day 5, day 8, day 11, and day 14). We performed statistical analysis to identify differentially expressed genes for each comparison using the negative-binomial model of read counts as implemented in the Biocondoctor DESeq package (Anders and Huber 2010). The same analysis was performed to compare TCDD-treated to control-treated AHR-positive cells at the same time points.

We used differential expression *p*-values in LRpath (http://lrpath.ncibi.org/) gene set enrichment analyses (Sartor et al. 2009a) to identify the top 100 Gene Ontology (GO)–affected categories in each group. These gene ontologies (The Gene Ontology; http://www.geneontology.org/) were hierarchically clustered based on the LRpath enrichment *z*-score, with positive values denoting up-regulation and negative values denoting down-regulation. Clustering was performed using the GENE-E algorithm (http://www.broadinstitute.org/cancer/software/GENE-E/). The gene expression data and results (Wang et al. 2013) have been deposited in the Gene Expression Omnibus (GEO) (Barrett et al. 2009) and can be accessed through Genomics Portals (http://GenomicsPortals.org) (Shinde et al. 2010) or the GEO.

To better understand our findings, we analyzed some RNA-seq data using Ingenuity Pathway Analysis (IPA; Ingenuity® Systems, http://www.ingenuity.com).

*Statistical analysis.* Significant genes were selected based on a false-discovery rate–adjusted *p*-value < 0.0001.

## Results

**Figure 1:**
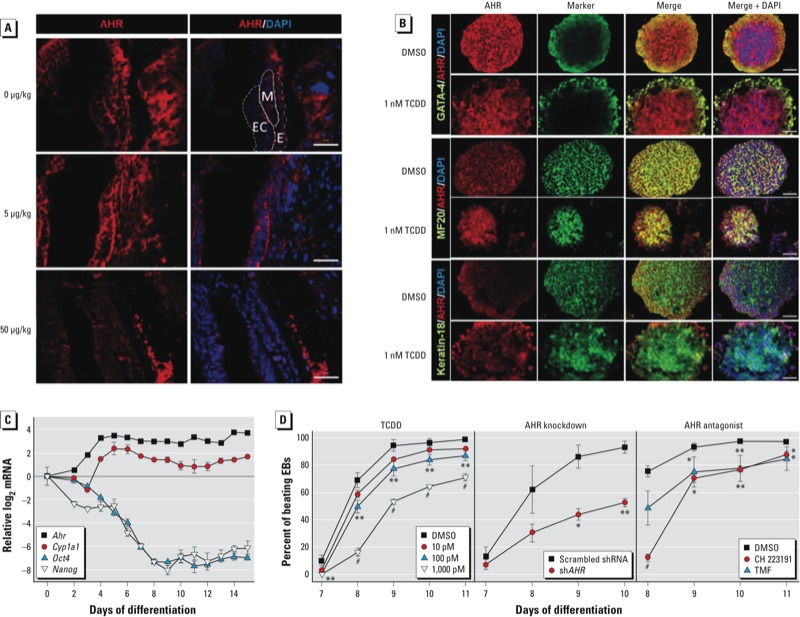
Detection of AHR in embryos (*A*), EBs (*B,C*), and cardio­myocytes (*D*). (*A*) Embryos were exposed to corn oil vehicle or TCDD (5 or 50 μg/kg) on GD5.5 and examined by immuno­fluorescence on GD7.5. Abbreviations: E, endoderm; Ec, ectoderm; M, mesoderm. (*B*) Immunofluorescence detection of lineage markers [AHR, GATA4, MF20 (cardiomyocytes), and keratin-18] in 3‑day-old EBs treated with TCDD (1 nM) or DMSO vehicle (≤ 0.05% in media). Columns 1 and 2 show immuno­fluorescence with AHR or the individual marker antibody, respectively; column 3 shows the merge of columns 1 and 2; and column 4 shows the merge of column 3 with the DAPI nuclear stain. Magnification for *A* and *B*, 20×; bars = 20 μm. (*C*) Expression patterns of *Ahr*, *Cyp1a1*, *Oct4/ (Pou5f1)*, and Nanog in differentiating EBs presented as the ratio of log2 qPCR mRNA level for each day of differentiation (normalized to Gapdh) to the corresponding level in ES cells (differentiation day 0). (*D*) Effect of TCDD treatment, Ahr knockdown, and AHR antagonists TMF and CH 223191 (10 μM each) on cardio­myocyte contractility in 3‑day-old EBs that were allowed to differentiate; EBs were examined daily under the microscope for the presence of a rhythmic beating phenotype. In *C* and *D*, data represent the mean ± SD of three independent experiments. ^*^p < 0.05, ^**^p < 0.01, and ^#^*p* < 0.001, compared with vehicle control by Bonferroni-corrected ANOVA.

*The AHR is expressed in mesendoderm of early embryos and ES cell EBs.* AHR expression in the developing mouse embryo has been detected as early as GD9.5 (Abbott et al. 1995). If the AHR has morphogenetic functions, its presence may be detectable at earlier times, in which case the choice of lineage and temporal-spatial expression pattern could indicate the role the AHR’s role in embryonic development. Using immunofluorescence, we were able to document AHR expression at GD7.5, considerably earlier than previously described by Abbott et al. (1995). By this time, AHR was already clearly expressed in all three embryonic germ layers (ectoderm, mesoderm, and endoderm) and in the surrounding decidual cells (Figure 1A). In all cell lineages of control embryos, the AHR localization was mainly cytosolic; however, in cells treated with 5 μg/kg TCDD, AHR localization was both cytosolic and nuclear, becoming almost completely nuclear in cells treated with 50 μg/kg TCDD (see Supplemental Material, Figure S1, for a higher magnification).

We examined AHR expression at earlier developmental times using pluripotent ES cells differentiated *in vitro* for which temporal expression patterns of markers of all three germ layers can readily be followed (Beddington and Robertson 1989). To assess AHR expression, we used immunofluorescence of 3-day-old EBs treated with TCDD or vehicle. We observed that endodermal cells comprise the outer cell layers of the EB, as shown by the presence of the endodermal marker GATA4 (Figure 1B). The inner cell mass of the EB consists of mesodermal and ectodermal cells, as shown by positive immunofluorescence with MF20 and keratin-18 antibodies, respectively. Control and TCDD-treated EBs show colocalization of AHR with GATA4 and MF20, and to a much lesser extent with keratin-18 (Figure 1B), suggesting that mesoderm and endoderm are the earliest cell lineages to express AHR.

We observed no expression of *Ahr* mRNA in ES cells, but it was detectable in 2-day-old EBs; *Ahr* expression gradually increased to a maximum by 6 days of differentiation and maintained a constant level for the next 8–9 days (Figure 1C). Expression of *Cyp1a1* mRNA followed a similar pattern, gradually increasing until differentiation day 6, when it reached a maximum, and then slowly decreasing to a minimum by day 12 and maintaining a similar level until day 15 (Figure 1C). Interestingly, *Cyp1a1* expression was independent of treatment with an exogenous AHR ligand, suggesting that during this period of early development, the AHR transcriptional functions are ligand independent or are regulated by an endogenous ligand. This finding is in good agreement with previous observations of constitutive *Cyp1a1* expression during early embryonic development in the mouse (Campbell et al. 2005). As we expected, expression of the pluripotency markers *Oct4* (*Pou5f1*) and *Nanog* declined gradually as the cells differentiated, down to their lowest level of expression on differentiation day 9 (Figure 1C).

*AHR activation, knockdown, and inhibition all block cardiomyocyte lineage differentiation.* Pluripotent ES cells have the potential to generate most embryonic cell lineages (Doetschman et al. 1985), including cardiomyocytes (Yamashita et al. 2005). Differentiation of ES cells into cardiomyocytes can be traced microscopically by visual examination of differentiating EBs that spontaneously develop a contractile phenotype. Importantly, beating cardiomyocytes derived from ES cell EBs function in all manners as cardiac cells, forming stable intracardiac grafts when injected into mice (Klug et al. 1996). In our previous study (Wang et al. 2010), we observed that treatment with 1 nM TCDD disrupted the beating phenotype. In the present study, we extended this observation by further characterizing the consequences of treatment with AHR antagonists or molecular inhibitors on cardiomyocyte development. Continuous exposure of differentiating cells to TCDD led to a dose-dependent inhibition of beating, which at 100 pM and 1 nM was significantly different from the control (Figure 1D, left). Knockdown of > 80% of AHR expression with a lentivirus expressing *Ahr* shRNA (see Supplemental Material, Figure S2) or treatment with the AHR antagonists TMF or CH 223191 also significantly decreased the number of beating EB-derived cultures (Figure 1D, center and right, respectively) without affecting cell survival. These results are a good indication that endogenous AHR signaling underlies homeostasis in cardiomyocyte differentiation and function independently of the potential toxicity of its exogenous agonist. Because this critical role can be disrupted by the opposing effects of AHR repression, inhibition, or ligand-mediated activation, it is reasonable to conclude that the level of functional AHR during cardiomyogenesis is a critical determinant of differentiation. That is, too little or too much of this protein adversely affects mesodermal lineage differentiation programs.

**Figure 2:**
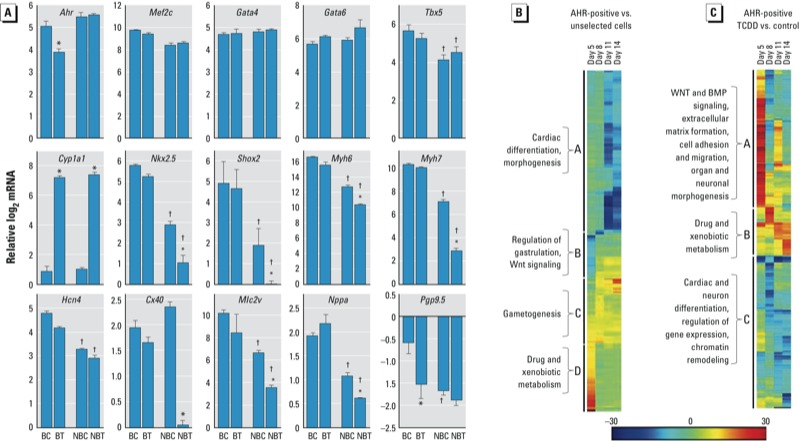
Cardiac marker expression in beating and non­beating differentiated ES cells and cluster analyses of gene expression changes regulated by AHR. (*A*) Cardiac marker expression in beating and non­beating areas from 12-day-old EBs treated with 1 nM TCDD or untreated (see Supplemental Material, Table S2). Data are presented as the log2 qPCR mRNA level (normalized to Gapdh) relative to the corresponding level in the control (mean ± SD). Abbreviations: BC, beating control; BT, beating TCDD treated; NBC, non­beating control; NBT, non­beating TCDD treated. (*B,C*) Hierarchical clustering of the top 100 GO categories by z-score using the GENE-E algorithm shown by (*B*) a heatmap of AHR-positive differentiated cells compared with unselected differentiated cells (see Supplemental Material, Table S3), and (*C*) a heatmap of AHR-positive differentiated cells treated with 1 nM TCDD (48 hr) compared with AHR-positive differentiated cells treated with DMSO vehicle (see Supplemental Material, Table S4). Salient categorical groups in each cluster are indicated. ^*^p < 0.05 compared with the corresponding control, by one-way ANOVA. ^†^*p* < 0.05 compared with the corresponding beating EBs, by one-way ANOVA.

*TCDD treatment disrupts the gene-expression trajectories of cardiac markers*. We previously observed that a 4-day treatment with TCDD after the completion of EB formation deregulated the expression of > 50 homeobox genes, many by as much as 50- to 100-fold above or below the control (Wang et al. 2010). To determine whether any of these changes were responsible for the effect of TCDD on the beating phenotype, in the present study we dissected beating and nonbeating regions of differentiating cultures treated with 1 nM TCDD or vehicle and used qPCR to measure the gene expression levels of several markers relevant to cardiac function (Figure 2A; see also Supplemental Material, Table S2). TCDD-treated cells that continued to beat showed no change in the expression of the markers tested compared with the vehicle control. However, TCDD treatment significantly repressed the expression of *Nkx2-5, Shox2, Myh6, Myh7, Cx40, Mlc2v, Hcn4*, and *Nppa* in nonbeating cells and induced *Cyp1a1* expression in both beating and nonbeating cells (Figure 2A). Interestingly, expression of *Pgp9.5*, a neuroendocrine marker and component of the cardiac conduction system (El Sharaby et al. 2001), was repressed under all conditions tested, indicating that cells of ectoderm lineage were not present in the beating or nonbeating nodes selected. These data suggest that TCDD inhibition of the beating phenotype is independent of TCDD’s role in xenobiotic metabolism, and that this inhibition is likely to be the consequence of silencing the expression of genes critical for the contractile phenotype.

*Gene ontology annotations of genes differentially expressed in AHR-positive cardiomyocytes*. Two major caveats must be considered when interpreting the data described above in the context of AHR-dependent gene expression: *a*) the differentiating cell population is a combination of cells of various lineages, where ≤ 30–40% of all cells are cardiomyocytes (Wang et al. 2010); and *b*) not all cells in the population express AHR. To insure that we tracked only cells positive for a functional AHR, we established the pAHRPuroIRESeGFP cell line, a stable ES cell line that expresses the selection markers puromycin resistance and eGFP under control of the *Cyp1a1* promoter, and therefore responds to TCDD treatment (see Supplemental Material, Figure S3A). These cells were > 90% pure (see Supplemental Material, Figure S3B) and did not overexpress AHR relative to the parental ES cells (see Supplemental Material, Figure S3C) but expressed mesodermal markers characteristic of cardiomyocyte cells (see Supplemental Material, Figure S3D).

We used global gene expression profiling at different times of differentiation to characterize the effect of TCDD-dependent AHR activation on gene expression in AHR-positive pAHRPuroIRESeGFP cardiomyocytes. Cells were allowed to differentiate for 2 days as hanging-drop EBs and then collected on days 5, 8, 11, and 14. To enrich for cells expressing AHR, we treated cells with 3 μg/mL puromycin for 3 days prior to collection in order to select for resistance. A population of untransfected and unselected ES cells was grown and sampled in parallel. To analyze gene expression changes across time, we compared *a*) AHR-positive cells with unselected cells, and *b*) AHR-positive cells treated with TCDD (1 nM) with vehicle-treated AHR-positive cells. In each comparison, several thousand genes had significant expression differences with false-discovery rate–adjusted *p*-value < 0.0001. We used these genes to identify the top 100 GO affected categories in each group, which were hierarchically clustered by *z*-score using the GENE-E algorithm developed by the Broad Institute Cancer Group (http://www.broadinstitute.org/cancer/software/GENE-E/). Relative to unselected cells, AHR-positive cells showed a time-dependent decrease of expression of GO categories involved in *a*) cardiac differentiation and morphogenesis, *b*) increasingly lower expression of categories involved in WNT (wingless-related MMTV integration site 3A) signaling and regulation of gastrulation, *c*) gametogenesis, and *d*) high levels of expression of genes involved in drug and xenobiotic metabolism (Figure 2B; see also Supplemental Material, Table S3).

TCDD treatment of AHR-positive cells identified three clusters of GO categories (Figure 2C). Cluster A includes categories involved in WNT and BMP (bone morphogenetic proteins) signaling, cell adhesion, and organ morphogenesis that are highly induced by TCDD-driven AHR activation at early time points but become repressed as differentiation proceeds. The opposite pattern is seen in cluster B, which includes genes involved in drug and xenobiotic metabolism. Cluster C includes genes with cardiac and neural differentiation functions, which are repressed by TCDD treatment (Figure 2C; see also Supplemental Material, Table S4). Two prominent pathways appear to be targeted by early AHR functions: the regulation of gastrulation and WNT signaling during embryogenesis, both of which are disrupted by TCDD treatment at the earlier time points. Cardiac and neural differentiation, extracellular matrix formation, and cell adhesion and migration are also early targets of TCDD in AHR-positive cells. These data clearly illustrate the intricacy of the AHR’s role during differentiation and the multiplicity of pathways triggered by TCDD-driven AHR activation responses.

**Figure 3:**
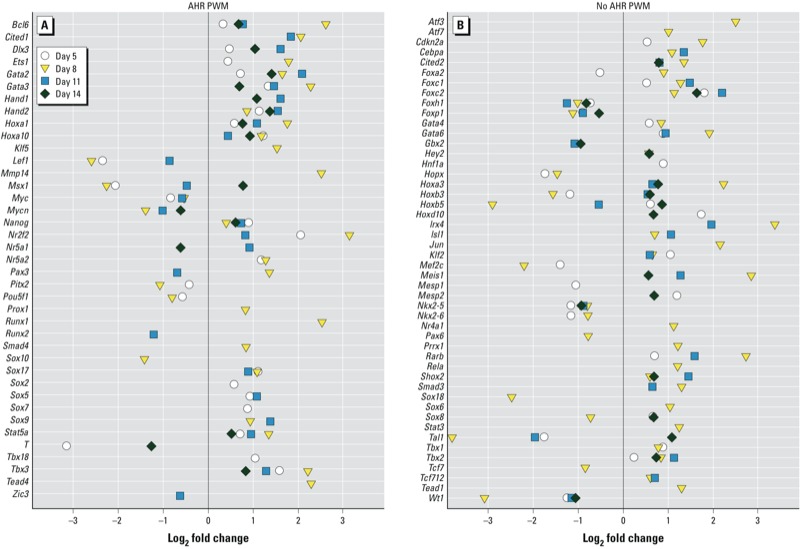
RNA‑seq expression changes of the 100 homeobox transcription factors associated with cardiovascular development that were deregulated by the AHR/TCDD axis. Genes positive (*A*) or negative (*B*) for AHR position weight matrix (PWM) anywhere between coordinates –10,000 and +1,000 nucleotides from the transcription start site. For gene names and gene IDs, see Supplemental Material, Table S6.

*The AHR/TCDD axis disrupts the expression of homeobox transcription factors and Polycomb and trithorax group (PcG and TxG, respectively) genes*. Our RNA-seq results indicated that a few thousand genes, comprising a significant fraction of the genome, were responsive to AHR/TCDD-mediated regulation. The most reasonable explanation for this finding is that the AHR is a master upstream regulator that controls the expression of homeobox transcription factors, which are responsible for the regulation of developmental gene expression in a tissue- and time-dependent fashion (Moreland et al. 2009). In agreement with this hypothesis, we found that 729 transcription factors, most homeodomain factors, were differentially expressed in TCDD-treated AHR-positive cells relative to control (see Supplemental Material, Table S5). From this group, 100 factors with *p*-values < 0.05 were specifically associated with cardiovascular development. To determine whether the AHR binding motif was present in the promoters of the genes coding for these factors, we used the TRANSFAC algorithm (Wingender 2008) to search for the presence of AHR position weight matrix motifs anywhere between –10,000 and +1,000 nucleotides from the transcription start site. Approximately 50% of the genes with log_2_ fold change ≤ 0.5 or > 0.5 had at least one, but often more than one, AHR binding site in this domain, whereas the other 50% did not. We observed no significant difference between these two groups in either the level or the timing of differential expression (Figure 3; see also Supplemental Material, Table S6).

PcG and TxG proteins constitute a group of critical regulators of epigenetic modifications affecting differentiation during development. They act coordinately or antagonistically to repress or promote transcription, respectively, throughout embryonic development (Schuettengruber et al. 2007). In agreement with the master regulatory role consistently shown by the AHR, our RNA-seq gene expression profiles detected the AHR/TCDD-dependent altered expression of 22 PcG and TxG genes in AHR-positive cells (see Supplemental Material, Figure S4 and Table S7).

**Figure 4:**
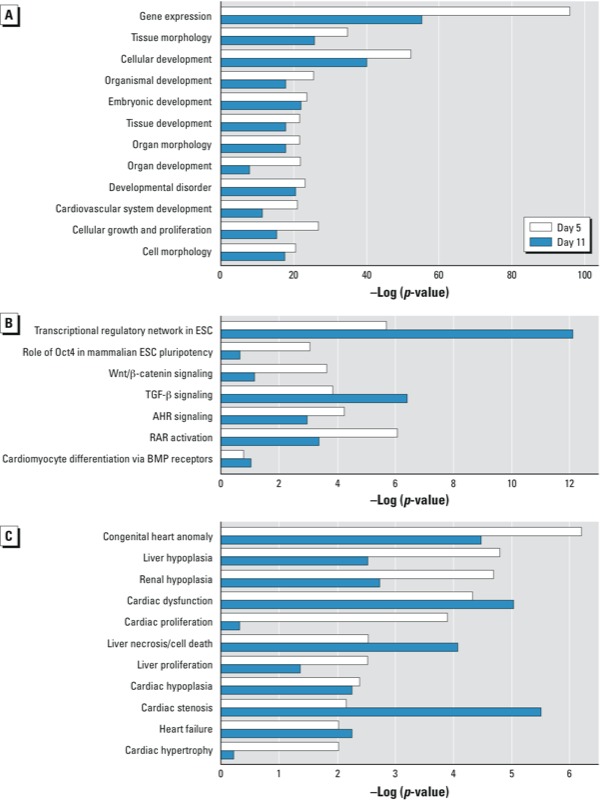
IPA results for gene expression changes induced by TCDD in AHR-positive cardio­myocytes shown by biological (*A*), canonical (*B*), and toxicological (*C*) function on days 5 and 11 of differentiation. Abbreviations: ESC, ES cells; RAR, retinoic acid receptor.

*Functional analyses of gene expression changes resulting from activation of the AHR/TCDD axis*. To better understand the molecular and chemical interactions elicited by TCDD treatment and their phenotypic effects on AHR-positive cells, we input the RNA-seq data for the 729 transcription factors into the Ingenuity Knowledge Base (Ingenuity®Systems) to analyze the AHR/TCDD axis–driven effects on biological, canonical, and toxicological functions by IPA. The most significant change in biological functions took place in gene expression functions, as could be expected from the effects observed on homeobox transcription factors. Other biological changes affected several aspects of embryonic, cardiovascular system, and tissue development; morphology; cell growth; and cell proliferation. These changes were more significant at early stages of differentiation: In all cases, the –log (*p*-value) was greater at day 5 than at day 11 (Figure 4A). Several canonical functions were also significantly affected by TCDD treatment, including transcriptional regulation and various signaling pathways, such as WNT, TGFβ (transforming growth factor β), AHR, and cardiomyocyte differentiation via BMP receptors. As in the case of biological functions, these effects were more significant at early day 5 (Figure 4B). The toxicological functions significantly affected by TCDD comprised a variety of cardiac end points, including congenital heart anomalies, cardiac dysfunction and proliferation, valvular stenosis, hypertrophy, and heart failure, as well as cardiac, liver, and renal hypoplasia (Figure 4C). These analyses indicate only that the pathways or functions are affected, but they do not provide the direction, activation, or inhibition of the effect. We searched the Ingenuity Knowledge Base for upstream regulatory molecules of the transcription factors involved in these functions and found close to 200 such regulators, of which 18–20 had significant *p*-values. When these were ranked by *z*-score, two groups were evident (Table 1). One group comprised regulators that were predicted to be inhibited, including TGFβ, BMP2/4, WNT1/3A, FGFR2 (fibroblast growth factor receptor 2), NFκB (nuclear factor kappa B), NKX2-5, Hedgehog, and a few others that regulate differentiation pathways. The second group included regulators of pluripotency pathways that were predicted to be activated, such as SOX2 (SRY-box containing gene 2), NANOG, KLF4 (Kruppel-like factor 4), and OCT4. The overall effect of TCDD-driven AHR activation during the early stages of differentiation appears to be to maintain the pro-proliferative state of the ES cells and inhibit their differentiation.

**Table 1:** Predicted activation state of upstream transcriptional regulators in TCDD-treated AHR-positive differentiating ES cells.

Differentiation day/ activation state	Regulator	*z*-Score	*p*-Value
Day 5
Inhibited	APLNR	–2.1	5.05 × 10^–7^
	BMP2	–2.4	1.57 × 10^–6^
	BMP4	–2.6	5.93 × 10^–17^
	FGFR2	–2.8	9.26 × 10^–11^
	GLI2	–2.4	3.31 × 10^–7^
	Hedgehog	–2.7	1.42 × 10^–19^
	MLL	–2.2	1.04 × 10^–12^
	NFκB	–2.2	5.33 × 10^–7^
	NKX2-5	–1.1	1.37 × 10^–8^
	SHH	–3.1	5.48 × 10^–13^
	TGFB1	–2.1	2.03 × 10^–7^
	TNF	–2.8	1.75 × 10^–7^
	WNT1	–2.9	1.92 × 10^–10^
	WNT3A	–2.9	7.34 × 10^–11^
Activated	GNL3	2.4	2.31 × 10^–8^
	POU5F1	2.3	1.85 × 10^–19^
	RNF2	2.2	3.01 × 10^–11^
	SOX2	2.3	1.78 × 10^–20^
Day 8
Inhibited	APLNR	–2.4	1.86 × 10^–9^
	BMP4	–3.3	2.81 × 10^–23^
	BMPR1A	–2.7	2.87 × 10^–9^
	CTNNB1	–2.2	2.91 × 10^–22^
	EPHB4	–2.5	8.04 × 10^–9^
	FGFR2	–2.9	4.73 × 10^–8^
	GLI2	–2.4	7.45 × 10^–12^
	MLL	–3.5	2.00 × 10^–29^
	NKX2-5	–0.5	2.98 × 10^–8^
	STAT3	–3.1	3.77 × 10^–8^
	TGFB1	–4.2	6.39 × 10^–15^
	Tretinoin	–2.5	3.41 × 10^–42^
	WNT11	–2.2	2.71 × 10^–8^
Activated	GNL3	2.6	3.28 × 10^–14^
	PHC2	2.6	3.85 × 10^–13^
	POU4F1	2.5	1.16 × 10^–17^
	POU4F2	2.4	5.39 × 10^–19^
	POU5F1	2.7	5.31 × 10^–26^
	RNF2	3.1	1.27 × 10^–16^
	SOX2	2.3	3.87 × 10^–26^
Day 11
Inhibited	ARID4B	–2.1	8.03 × 10^–7^
	BMP2	–2.5	3.10 × 10^–15^
	BMP7	–2.2	2.25 × 10^–8^
	BMPR1A	–2.6	5.76 × 10^–7^
	GLI3	–2.4	1.48 × 10^–11^
	HOXA9	–2.2	9.81 × 10^–10^
	MLL	–3.1	2.58 × 10^–30^
	NKX2-5	–0.7	5.94 × 10^–8^
	SMO	–2.1	7.57 × 10^–10^
	STAT3	–2.6	4.71 × 10^–7^
	TGFB1	–3.4	1.48 × 10^–10^
	Tretinoin	–2.8	1.63 × 10^–36^
Activated	KLF4	2.1	3.18 × 10^–7^
	NANOG	2.1	2.68 × 10^–18^
	OCT4	2.1	1.14 × 10^–9^
	PHC2	2.4	8.70 × 10^–11^
	POU4F2	2.2	7.99 × 10^–15^
	POU5F1	2.5	1.19 × 10^–25^
	RNF2	2.1	1.99 × 10^–15^
	SOX2	2.3	1.07 × 10^–22^
Day 14
Inhibited	ARID4A	–2.1	1.31 × 10^–6^
	ARID4B	–2.1	5.62 × 10^–7^
	BMP2	–2.5	4.31 × 10^–10^
	EPHB4	–2.1	6.34 × 10^–11^
	GLI1	–2.4	8.28 × 10^–7^
	GSC	–2.1	2.56 × 10^–6^
	HDAC	–2.2	3.26 × 10^–10^
	HOXA9	–2.2	1.61 × 10^–8^
	miR-34a-5p	–2.2	2.72 × 10^–8^
	MLL	–2.9	8.01 × 10^–27^
	NKX2-5	–0.7	2.98 × 10^–8^
	SPRY1	–2.2	3.88 × 10^–7^
	STAT3	–3.1	4.67 × 10^–7^
	TGFB1	–3.3	5.52 × 10^–8^
	Tretinoin	–3.1	1.49 × 10^–32^
Activated	PHC2	2.4	5.07 × 10^–11^
	SOX2	2.2	4.68 × 10^–22^

## Discussion

Our results show that AHR activation by TCDD during differentiation of AHR-positive ES cells suppressed the development of the contractile cardiomyocyte phenotype. Concomitantly, activation of the AHR/TCDD axis disrupted the concerted expression of genes that regulate multiple differentiation pathways, including WNT and BMP; genes coding for developmental processes such as gametogenesis, cardiac and neural differentiation, extracellular matrix formation, and cell adhesion and migration; and genes encoding chromatin remodeling factors. Remarkably, a similar pattern of TCDD regulatory effects have been described in the regeneration of adult zebrafish hearts (Hofsteen et al. 2013). In the present study, the pattern of TCDD-induced regulatory effects seemed to be more pronounced in the early stages of development (i.e., during days 5 and 8 of ES cell differentiation), similar to the pattern reported in zebrafish (Lanham et al. 2012), and was accompanied by parallel changes in the expression of genes encoding homeobox transcription factors and PcG and TxG proteins. Furthermore, when beating and nonbeating cardiomyocytes were analyzed separately after TCDD treatment, beating cardiomyocytes retained the expression of the cardiac markers *Nkx2-5, Shox2, Myh6, Myh7, Mlc2v*, and *Cx40* regardless of treatment, whereas nonbeating cells treated with TCDD did not express these markers. These results strongly indicate a causal connection between AHR function, TCDD treatment, and disruption of cardiomyocyte function. Moreover, because both AHR knockdown and its functional inhibition by antagonists suppressed the beating phenotype just as efficiently as TCDD-dependent AHR activation, it is reasonable to conclude that too much or too little functional AHR is equally deleterious to cardiomyocyte function and that the amount of AHR protein itself is a determinant of cardiomyocyte homeostasis.

In addition to metabolic xenobiotic detoxification, the AHR plays an important role in maintenance of cellular homeostasis, often in the absence of a xenobiotic ligand (Bock and Köhle 2006). A physiological role for the receptor independent of xenobiotic ligand has been recognized in *Ahr*-null mice (Gonzalez and Fernandez-Salguero 1998), which show, among others, an impaired cardiovascular phenotype with retained fetal vascular structures in the liver and eye that fail to undergo apoptosis (Lahvis et al. 2005). Comparing gene expression profiles of AHR-positive and unselected cells allowed us to assess which developmental AHR functions may be independent of an exogenous ligand. Expression of genes controlling functions such as cardiac differentiation, regulation of WNT signaling, gametogenesis, and gastrulation were enriched in AHR-positive cells relative to unselected cells. In contrast, genes regulating extracellular matrix formation, cell adhesion and migration, neural differentiation, and chromatin remodeling were deregulated only after TCDD treatment of AHR-positive cells. These two groups of functions may respond to activation by endogenous and exogenous ligands, respectively, segregating physiological processes regulated by an endogenous ligand-activated AHR from toxicological or adaptive responses dependent on AHR activated by a xenobiotic ligand. In this context, it is significant that constitutive expression of *Cyp1a1*, a gene that is normally silent in the absence of ligand, was significantly derepressed during differentiation in the absence of TCDD, suggesting a response to either ligand-independent AHR activation or to activation by an endogenous ligand. Elevated constitutive *Cyp1a1* mRNA levels have also been found *in vivo* in studies of fertilized mouse ova; and were attributed to the need for catalytically active CYP1A1 that might ensure rapid metabolism of unwanted CYP1A1 substrates during critical moments of early development (Dey and Nebert 1998).

A major problem in the interpretation of data pertaining to individual regulatory networks in a mixed-lineage cell population, such as differentiating ES cells, is lineage diversity. We adopted a promoter-mediated dominant selection system, previously established for the characterization of the cardiomyocyte transcriptome (Doss et al. 2007), in order to enrich for a population of AHR-positive cells. These cells, when established as a continuously growing cell line, expressed mesodermal markers specific to the cardiomyocyte lineage. In cells treated with TCDD, global gene expression changes showed the disruption of developmental WNT and BMP signaling pathways. BMP and WNT signaling during pre- and postimplantation embryonic development and their role during cardiomyocyte differentiation have long been recognized (Wang and Dey 2006). In mice, cooperative control of SMAD and WNT signaling pathways activates multiple transcription factors including *Gata4, Nkx2-5*, and *Mef2c*, which control cardiac differentiation (Pal and Khanna 2006). Similarly, temporal modulation of canonical WNT signaling in human pluripotent stem cells results in robust cardiomyocyte differentiation (Lian et al. 2013). Importantly, extensive work in zebrafish has demonstrated the disruption of WNT signaling by TCDD (Hofsteen et al. 2013; Lanham et al. 2012; Mathew et al. 2009).

Homeodomain transcription factors specify the progression of tissue differentiation and embryonic identity during development (Wang et al. 2009). They encode transcription factors that control the expression of multiple developmental gene batteries. Disrupted expression or mutations in these genes result in severe to lethal outcomes for the organism (Wang and Dey 2006). In humans, mutations in 25 different homeobox transcription factors have been found in patients with congenital heart disease (McCulley and Black 2012); expression of 14 of these (*Cited2, Ets1, Foxh1, Gata4, Gata6, Hand1, Hand2, Hoxa1, Irx1, Nkx2-5, Nkx2-6, Pitx2,* and *Tbx1*) was disrupted by TCDD in our mouse ES cell differentiation experiments. Two of these, *Nkx2-5* and *Gata4*, play a central role in cardiac development. *Nkx2-5* is genetically upstream of multiple genes essential for heart development; 33 heterozygous loss-of-function mutations in this gene have been reported to cause heart malformations in humans, including conduction delay and atrial septal dysmorphogenesis (Biben et al. 2000). In mice, homozygous *Nkx2-5* null embryos have shown arrested cardiac development after looping, poor development of blood vessels, and disturbed expression of cardiac genes (Tanaka et al. 1999). Mutations in *Gata4* have been associated with cardiac septal defects (Tomita-Mitchell et al. 2007). These transcription factors do not act alone; their cooperation and interdependent regulation is essential for cardiac development, such that disruption of the expression of any one gene leads to the imbalance of the overall transcriptional network. *Nkx2-5* and *Gata4* are mutual cofactors for each other; their coexpression leads to synergistic, rather than additive, activation of target genes (Riazi et al. 2009) and promotion of cardiomyocyte differentiation (Hiroi et al. 2001). Hence, disruption of homeobox gene expression, a downstream target of the AHR/TCDD axis, is potentially a major component of the inhibition of cardiomyocyte function by TCDD. Interestingly, > 50% of the homeobox genes regulated by the AHR do not have canonical AHR response sites in their promoters, suggesting that their regulation by the AHR may result from a complex combinatorial network of regulatory interactions that reaches beyond direct AHR signaling. Some of these interactions are likely to include epigenetic modifications of histone marks because TCDD induces deregulation of PcG and TxG genes.

## Conclusion

Results of the present study add to the growing body of evidence in all experimental systems tested to date that the AHR is a major contributor to cardiovascular homeostasis. Changes in the homeostatic gene expression levels regulated by the AHR pathway disrupt cardiomyocyte differentiation whether the AHR level is increased (if further activated by TCDD) or decreased (if inhibited by antagonists or shRNA). The significant role that the AHR plays in cardiovascular development makes the heart a very sensitive target of fetal environmental injury.

## Supplemental Material

(3.5 MB) PDF
