# Supplementary material for: Disruption of Aryl Hydrocarbon Receptor Homeostatic Levels during Embryonic Stem Cell Differentiation Alters Expression of Homeobox Transcription Factors that Control Cardiomyogenesis
Source: Environ Health Perspect. 2013 Sep 20;121(11-12):1334–43. doi: 10.1289/ehp.1307297 (PMC3855521; doi:10.1289/ehp.1307297)
Supplement: (3.5 MB) PDF [file ehp.1307297.s001.508.pdf]

Environmental Health Perspectives doi:10.1289/ehp.1307297

Supplemental Material

Disruption of Aryl Hydrocarbon Receptor Homeostatic Levels during Embryonic Stem Cell Differentiation Alters Expression of Homeobox Transcription Factors that Control Cardiomyogenesis

Qin Wang, Jing Chen, Chia-I Ko, Yunxia Fan, Vinicius Carreira, Yinglei Chen, Ying Xia, Mario Medvedovic, and Alvaro Puga

Department of Environmental Health and Center for Environmental Genetics, University of Cincinnati College of Medicine, Cincinnati, Ohio, USA

Table of Contents

Supplemental Table S1. Primer sequences and product size for qPCR analysis of mRNA expression of the indicated genes. 3

Supplemental Table S2. Cardiac Marker Genes Affected in Their Expression by TCDD Treatment of Differentiating Mouse ES Cells. 4

Supplemental Table S3. Z-Scores of Top 100 GO Terms AHR-Positive *vs* Unselected Cells. 7

Supplemental Table S4: Z-Scores of Top 100 GO Terms AHR-Positive 48 h TCDD vs Control Cells. 14

Supplemental Table S5. Expression levels of transcription factors that show differential expression between TCDD-treated AHR-positive cells and controls (Log2Fold change) 23

Supplemental Table S6. Expression levels of transcription factors that regulate cardiomyocyte differentiation in TCDD-treated AHR-positive cells relative to controls (Log2 Fold change) ... 42

Supplemental Table S7. Summary of PcG and TxG genes affected in their expression by TCDD treatment in AHR-positive cells 45

Supplemental Figure S1. Higher magnification of the GD7.5 embryos shown in Fig. 1A. 48

Supplemental Figure S2. Stable integration of an *Ahr* ShRNA lentivirus inhibits AHR expression. 49

Supplemental Figure S3. Characterization of AHR-positive cardiomyocytes. 50

Supplemental Figure S4. Time-dependent expression changes of PcG and TxG genes deregulated by the AHR/TCDD axis. 52

References 53

Supplemental Table S1. Primer sequences and product size for qPCR analysis of mRNA expression of the indicated genes.

Gene	**Forward primer sequence (5'-> 3')**	**Reverse primer sequence (5'-> 3')**	**PCR product size (bp)**
Ahr	GGCCAAGAGCTTCTTTGATG	TGCCAGTCTCTGATTTGTGC	93
Cx40	AAGGCTCGGCCTCGGTCTCC	CCCAGGACCAGCATGCGGAA	146
	CTGGCCACTGGGGAAGTGCC	TAGCCCTGAGGAAGGCGGTGG	125
Cyp1a1	GTGTCTGGTTACTTTGACAAGTGG	AACATGGACATGCAAGGACA	199
Ece1	CAACGGGGGACTCAAGGCGG	TGGTGAGACCCAGGGTGGGC	92
	GAGACGGCGCTGGCCAACAT	TGGCAGGTGCCAAGGTCTGC	106
Gata4	TCTCACTATGGGCACAGCAG	GCGATGTCTGAGTGACAGGA	136
Gata6	TCGAAACGCCGGTGCTCCAC	GTTCACGCACTCGCGGCTCT	116
	CGGTCGCGGCCGTTCTTCTC	CACCAGCCGCCGTCAGTCAA	97
Hcn4	CCACCACTGGGTTCGGCCAC	GCAGCCTGTGGAGAGCGAGC	109
Kdr	GGCGGTGGTGACAGTATCTT	CTCGGTGATGTACACGATGC	189
Mef2c	ACGCCTGTCACCTAACATC	TTTCCCTTTCCTTTCCTTTCC	150
Mlc2v	CCCGAGGGCAAAGGGTCACT	GACGTCAGGGGGAAAGGCTGC	117
	TGGCAACTGGCCTCAGACACC	ACGTTGGAGCTCCCGCCTTCT	71
Myh6	CTGCTGGAGAGGTTATTCCTCG	GGAAGAGTGAGCGGGGCATCAAGG	302
Myh7	TGCAAGGCTCCAGGTCTGAGGGC	GCCAACACCAACCTGTCCAAGTTC	202
Nanog	AGGGTCTGCTACTGAGATGCTCTG	CAACCACTGGTTTTTCTG CCACCG	364
Nkx2-5	CGACGGAAGCCACGCGTGCT	CCGCTGTCGCTTGCACTT	180
Nppa (Anf)	AGCCCAGAGTGGACTAGGCTGC	TGCGTGACACACCACAAGGGC	123
	TCAGATCGTGCCCCGACCCA	GGCCTGGAAGCCAAAAGGCCA	88
Oct4	GGCGTTCTCTTTGGAAAGGTGTTC	CTCGAACCACATCCTTCTCT	313
Pgp9.5 (Uchl1)	TGCAGGTGCCATCCGCGAAG	CCCTAGCACGTCGGCGAAGC	119
	CCATGGCGCCAGCTCAGAGG	CCACGGCAGAGAAGCGGACC	101
Shox2	GGAGCTGGACATGGGA	GCCTCTGCTTGATTTTGGTC	140
Tbx3	CCCCGCTACGGGGGAGCAAT	TGGAACCGCGGCTGGTACTT	129/189
	CAAAGAGCGTGGGAGCCGGG	CAAGCAGGGGCTCGACTGGC	98
Tbx5	TACCCCGCGCCCACTCTCAT	TGCGGTCGGGGTCCAACACT	120
	CCTGTGGTCAGGCAGTGCGG	CTGGGCACGCCGTGAGTGTA	92

Supplemental Table S2. Cardiac Marker Genes Affected in Their Expression by TCDD Treatment of Differentiating Mouse ES Cells.

Gene	Description	Function	Expression	Reference
Cx40	Connexin gene consists of 20 members in mouse. Four isoforms Cx 40, Cx43, Cx45, Cx37 are known as "cardiovascular connexins", playing roles in cardiovascular morphology and function.	Major gap-junction protein in the atrial myocardium. Gap junctions are responsible for cell-cell conduction of the action potential.	Expressed mainly in the atrial working myocardium, the conduction system and the vasculature.	(Sohl and Willecke 2004; Chaldoupi et al. 2009)
Ece1	endothelin-1 converting enzyme-1, type II metallorprotease, Four isoforms Ece1a, Ece1b, Ece1c and Ece1d	Cleave endothelin precursor big-endothelin-1, a 38-amino-acid peptide, to yield functional form of endothilin-1; endothelin-1 and ECE play a role in endocrine function as well as growth and development. ECE-1 is involved in the pathogenesis of disease states including cancer, cardiovascular disease and Alzheimer's disease.	Exists as a dimer on the cell surface	(Kuruppu and Smith 2012)
Gata4	Belong to Zinc finger super family, members of this family including GATA 1-6. Regulate cell-fate specification and differentiation, and control cell proliferation and movement.	Essential for the hypertrophic response; induce the formation of cardiac myocytes in embryonic carcinoma cells.	GATA4/5/6 mainly expressed in endodermally derived tissues including heart, lung, stomach, intestine, ovary blood vessels, etc.	(Patient and McGhee 2002)
Gata6	Refer to the above cell.	Synergistically with NFAT activated smooth-muscle-specific gene transcription to maintain differentiated Vascular smooth muscle cells.	Refer to the above cell.	(Patient and McGhee 2002)
Hcn4	Hyperpolarization-activated cyclic nucleotide-gated channel; members includes HCN 1-4.	"Pacemaker channels", help generate rhythmic activity within groups of heart and brain cells.<br>In the embryo, cAMP-bound HCN4 is a powerful pacemaker. In adult mice, HCN4 ensures stable heart rhythm during and after stress, not pacemaking.	Prominently expressed in the pace maker region of the mammalian heart, also expressed in the brain.	(Stieber et al. 2003; Harzheim et al. 2008; Biel et al. 2009)
Kdr	Kinase insert domain-containing receptor/fetal liver kinase-1 (Flk1),also called VEGFR2 (vascular endothelial growth factor receptor -2) CD309 (cluster of differentiation 309). A type III receptor tyrosine kinase	Required for the differentiation of endothelial progenitor cells (EPCs) and for the movement of EPCs from the posterior primitive streak to the yolk sac, a precondition for the subsequent formation of blood vessels; plays a critical roles in the development and formation of blood vessel networks.	EPCs, endothelial cells, primitive and more mature hematopoietic cells.	(Wang et al. 2007)
Mef2c	Belongs to myocyte enhancer factor-2 family, also referred to as RSRF (Related to Serum Response Factor); members in this gene family include Mef2 a-d.	Pivotal role in morphogenesis and myogenesis of skeletal, cardiac and smooth muscle cells.<br>Regulates cardiac hypertrophy and remodeling.	In all developing muscle cells, non-muscle cells including brain and lymphoid tissue.	(Blackand Olson 1998; Pereira et al. 2009)

**Gene**	**Description**	**Function**	**Expression**	**Reference**
Mlc2v	Regulatory (phosphorylatable) myosin light chain , three members *Mlc2a*, *Mlc2v* and *Mlc2f* belong to this group.	Associated with the cardiac myosin beta heavy chain, Ca^{2+} triggers the phosphorylation of regulatory light chain which in turn triggers contraction.	Ventricular myocardium restricted.	(Franco et al. 1998)
Myh6/7	Myosin heavy chain	A ubiquitous eukaryotic motor protein; interacts with actin to generate the force for cellular movements ranging from cytokinesis to muscle contraction.	Myh6 is expressed in the adult ventricles and atria, exclusively restricted to the heart. Myh7 is expressed in both cardiac and slow (type I) skeletal muscle fibers; the abundant isoform in the ventricles of all mammals during fetal life.	(Weiss and Leinwand 1996)
Nkx2-5	Nkx2 homeobox 5, family members including Nkx2-3, Nkx2-5, Nkx2-6 and Nkx2-7	Early role in cardiomyocyte specification and differentiation and late role in cardiac morphogenesis.	First detected in mesodermal cells specified to form heart at ED 7.5 in mouse and the expression is maintained throughout the developing and adult heart. Transiently expressed in the developing pharynx, thyroid and stomach.	(Harvey 1996; Lien et al. 1999)
Nppa	Atrial natriuretic peptide (ANF)	An inhibitor of renal tubular reabsorption of NaCl produced and excreted by the heart. ANF promoter is a transcriptional target for both GATA-4 and NKX2-5; Induction of *Nppa* is a conserved feature of ventricular hypertrophy.	Expression of *Nppa* is an early and specific marker for the differentiating working myocardium of the atria and ventricles of the developing heart.	(Durocher et al. 1997; Houweling et al. 2005)
Pgp9.5	Protein gene product 9.5	A specific tissue marker for the neuroendocrine system; corresponds with abnormal ventricular automaticity.	Cytoplasm of neuronal cells; morphologically dynamic myocardial regions during heart ontogenesis; expressed in myocardial sleeves, cardiac nerves and components of the cardiac conduction systems.	(El Sharaby et al. 2001; Otsuki et al. 2004)
Shox2	Short stature homeobox 2	Pacemaking function; an essential transcription factor for the differentiation of cardiac pacemaker cells through repressing NKX2-5.	Restricted to the sinus venosus region and eventually to SAN, including the pacemaker and sinus valves in the developing heart.	(Espinoza-Lewis et al. 2009)

Gene	Description	Function	Expression	Reference
Tbx3	Belongs to T-box gene family encoding more than 20 transcription factors, which share a highly conserved DNA-binding region.	Inhibition of chamber myocardium, conduction system pattering.	Non-chamber myocardium of the atrioventricular canal (AVC) during cardiac development; epithelial and neural crest cells in the pharyngeal region, in the sinoatrial node (SAN) primordium, and in all mature components of the central conduction system.	(Greulich et al. 2011)
Tbx5	Refer to the above cell.	Chamber formation, septation and cardiomyocyte differentiation.	First expressed in bilateral wings of the cardiogenic mesoderm and later in the endocardium and myocardium of the inflow region, the atria, the AVC and the left ventricle.	(Greulich et al. 2011)

Supplemental Table S3. Z-Scores of Top 100 GO Terms AHR-Positive *vs* Unselected Cells.

Cluster	GO Term	Name	Day 5	Day 8	Day 11	Day 14
A	GO:0061311	cell surface receptor signaling pathway involved in heart development	-9.89	-3.06	-16.16	-10.56
A	GO:0005615	extracellular space	-7.71	-3.21	-9.62	-10.57
A	GO:0009887	organ morphogenesis	-5.17	-2.73	-8.57	-9.78
A	GO:0001501	skeletal system development	-4.42	-2.70	-8.07	-9.56
A	GO:0001944	vasculature development	-4.74	-3.08	-8.23	-10.95
A	GO:0001568	blood vessel development	-4.68	-3.12	-8.40	-10.89
A	GO:0072358	cardiovascular system development	-5.11	-3.26	-9.45	-12.58
A	GO:0072359	circulatory system development	-5.11	-3.26	-9.45	-12.58
A	GO:0007507	heart development	-4.54	-3.39	-11.19	-11.08
A	GO:0048738	cardiac muscle tissue development	-4.00	-3.07	-10.26	-11.32
A	GO:0061371	determination of heart left/right asymmetry	-6.68	-2.47	-10.31	-7.03
A	GO:0001947	heart looping	-6.62	-2.54	-10.60	-7.28
A	GO:0003143	embryonic heart tube morphogenesis	-6.27	-2.40	-10.08	-6.93
A	GO:0003209	cardiac atrium morphogenesis	-5.60	-2.22	-12.36	-9.40
A	GO:0003230	cardiac atrium development	-5.49	-2.16	-12.09	-9.44
A	GO:0035051	cardiac cell differentiation	-5.40	-2.55	-11.66	-9.20
A	GO:0003151	outflow tract morphogenesis	-5.23	-2.20	-10.85	-9.04
A	GO:0060976	coronary vasculature development	-4.02	-3.18	-12.00	-9.16
A	GO:0048762	mesenchymal cell differentiation	-3.96	-2.20	-11.26	-9.28
A	GO:0060485	mesenchyme development	-3.83	-2.38	-11.22	-9.46
A	GO:0014031	mesenchymal cell development	-3.63	-2.20	-11.06	-8.51
A	GO:0048644	muscle organ morphogenesis	-2.71	-2.89	-11.60	-10.28
A	GO:0048483	autonomic nervous system development	-2.52	-2.17	-11.12	-9.32
A	GO:2000826	regulation of heart morphogenesis	-4.23	-2.36	-14.18	-8.94
A	GO:0005110	frizzled-2 binding	-3.76	-2.15	-14.26	-6.93
A	GO:0005518	collagen binding	-3.75	-0.73	-12.38	-5.34
A	GO:0033613	activating transcription factor binding	-3.24	-3.13	-9.92	-6.86
A	GO:0060317	cardiac epithelial to mesenchymal transition	-2.39	-1.97	-10.33	-6.13
A	GO:0048538	thymus development	-1.78	-1.65	-10.24	-6.07
A	GO:0060043	regulation of cardiac muscle cell proliferation	-1.80	-2.19	-9.91	-7.38
A	GO:0045668	negative regulation of osteoblast differentiation	-1.08	-2.10	-10.48	-7.18
A	GO:0060716	labyrinthine layer blood vessel development	-0.75	-2.05	-10.06	-5.26
A	GO:0060840	artery development	-2.53	-2.44	-10.22	-7.96
A	GO:0048844	artery morphogenesis	-2.52	-2.37	-10.67	-8.32
A	GO:0060045	positive regulation of cardiac muscle cell proliferation	-2.04	-1.95	-11.46	-8.22

Cluster	GO Term	Name	Day 5	Day 8	Day 11	Day 14
A	GO:0043392	negative regulation of DNA binding	-1.72	-1.70	-11.07	-7.68
A	GO:0043388	positive regulation of DNA binding	-0.93	-1.71	-10.90	-8.50
A	GO:0033275	actin-myosin filament sliding	-0.76	-1.55	-10.05	-8.57
A	GO:0001755	neural crest cell migration	-1.13	1.94	-10.66	-7.46
A	GO:0003156	regulation of organ formation	-5.90	-2.94	-16.70	-10.11
A	GO:0048645	organ formation	-5.56	-3.03	-15.35	-9.70
A	GO:0060914	heart formation	-4.13	-2.70	-17.43	-11.62
A	GO:0003206	cardiac chamber morphogenesis	-4.34	-3.40	-15.44	-13.47
A	GO:0003205	cardiac chamber development	-4.32	-3.36	-15.04	-13.29
A	GO:0048485	sympathetic nervous system development	-3.43	-3.09	-15.12	-12.83
A	GO:0003007	heart morphogenesis	-4.74	-3.26	-13.23	-11.08
A	GO:0003208	cardiac ventricle morphogenesis	-2.58	-2.77	-13.63	-12.85
A	GO:0003231	cardiac ventricle development	-3.18	-2.53	-11.80	-11.99
A	GO:0055008	cardiac muscle tissue morphogenesis	-2.07	-3.24	-13.12	-11.78
A	GO:0060415	muscle tissue morphogenesis	-1.95	-3.05	-12.41	-11.12
A	GO:0060039	pericardium development	-2.51	-2.49	-14.53	-11.17
A	GO:0010463	mesenchymal cell proliferation	-3.00	-2.72	-15.63	-10.81
A	GO:2000677	regulation of transcription regulatory region DNA binding	-2.00	-2.47	-15.93	-10.76
A	GO:0060977	coronary vasculature morphogenesis	-1.55	-3.34	-13.86	-10.03
A	GO:0003215	cardiac right ventricle morphogenesis	-2.11	-3.77	-17.54	-12.81
A	GO:0042415	norepinephrine metabolic process	-1.12	-2.62	-18.24	-11.56
A	GO:0010658	striated muscle cell apoptosis	-0.64	-2.35	-15.71	-13.67
A	GO:0010659	cardiac muscle cell apoptosis	-0.64	-2.35	-15.71	-13.67
A	GO:0010656	negative regulation of muscle cell apoptosis	-0.61	-2.41	-16.00	-15.26
A	GO:0061046	regulation of branching involved in lung morphogenesis	-1.68	-2.44	-16.73	-8.35
A	GO:0060431	primary lung bud formation	-1.55	-2.40	-16.68	-8.09
A	GO:0060501	positive regulation of epithelial cell proliferation involved in lung morphogenesis	-0.76	-2.43	-16.83	-8.87
A	GO:0060502	epithelial cell proliferation involved in lung morphogenesis	-0.91	-2.06	-14.19	-7.56
A	GO:2000794	regulation of epithelial cell proliferation involved in lung morphogenesis	-0.66	-2.19	-15.34	-8.08
A	GO:0043586	tongue development	-1.07	-2.41	-14.87	-9.62
A	GO:0001967	suckling behavior	-0.82	-2.27	-15.95	-10.17
A	GO:0009713	catechol-containing compound biosynthetic process	0.81	-2.16	-14.88	-9.67
A	GO:0034312	diol biosynthetic process	0.81	-2.16	-14.88	-9.67
A	GO:0042423	catecholamine biosynthetic process	0.81	-2.16	-14.88	-9.67
A	GO:0010660	regulation of muscle cell apoptosis	-0.35	-1.82	-12.50	-12.06
A	GO:0010657	muscle cell apoptosis	-0.27	-1.61	-11.32	-10.84

Cluster	GO Term	Name	Day 5	Day 8	Day 11	Day 14
A	GO:0070888	E-box binding	0.98	-1.66	-12.77	-9.50
A	GO:0022610	biological adhesion	-3.77	-6.67	-5.14	-9.41
A	GO:0007155	cell adhesion	-3.62	-6.70	-5.17	-9.42
A	GO:0019838	growth factor binding	-2.54	-2.86	-5.75	-10.67
A	GO:0005201	extracellular matrix structural constituent	-2.14	-3.24	-5.98	-10.57
A	GO:0071230	cellular response to amino acid stimulus	-1.59	-3.90	-6.08	-12.97
A	GO:0071229	cellular response to acid	-1.73	-3.56	-5.50	-11.74
A	GO:0043200	response to amino acid stimulus	-1.34	-3.49	-5.46	-11.58
A	GO:0071417	cellular response to organic nitrogen	-1.14	-3.52	-5.53	-11.68
A	GO:0071418	cellular response to amine stimulus	-1.22	-3.58	-5.61	-11.89
A	GO:0014075	response to amine stimulus	-0.69	-2.68	-4.35	-9.78
A	GO:0070206	protein trimerization	-1.87	-1.56	-2.37	-9.83
A	GO:0048012	hepatocyte growth factor receptor signaling pathway	-1.49	-1.45	-2.67	-9.97
A	GO:0051450	myoblast proliferation	-0.15	-0.37	-2.05	-10.97
A	GO:0003810	protein-glutamine gamma-glutamyltransferase activity	-1.09	-0.12	-0.10	-9.42
A	GO:0030199	collagen fibril organization	-0.68	-3.36	-6.16	-16.12
A	GO:0070208	protein heterotrimerization	-0.13	-1.94	-4.02	-16.05
A	GO:0048407	platelet-derived growth factor binding	-2.78	-6.01	-8.67	-16.53
A	GO:0044420	extracellular matrix part	-2.20	-3.81	-11.88	-17.14
A	GO:0005578	proteinaceous extracellular matrix	-3.53	-9.93	-15.05	-20.78
A	GO:0031012	extracellular matrix	-3.47	-9.79	-14.34	-20.24
A	GO:0005581	collagen	-1.56	-3.08	-15.86	-22.25
A	GO:0060973	cell migration involved in heart development	-6.02	-4.27	-24.85	-18.01
A	GO:0061323	cell proliferation involved in heart morphogenesis	-1.99	-3.50	-23.91	-16.06
A	GO:2000679	positive regulation of transcription regulatory region DNA binding	-1.97	-3.73	-24.32	-16.05
A	GO:0061307	cardiac neural crest cell differentiation involved in heart development	-1.77	-3.46	-24.20	-16.53
A	GO:0061308	cardiac neural crest cell development involved in heart development	-1.97	-3.81	-26.55	-18.17
A	GO:0003680	AT DNA binding	-1.78	-3.76	-25.51	-17.23
A	GO:0042421	norepinephrine biosynthetic process	-1.72	-3.82	-25.97	-16.69
A	GO:0005583	fibrillar collagen	-1.31	-6.15	-28.49	-18.32
A	GO:0071526	semaphorin-plexin signaling pathway	-4.62	-3.49	-17.65	-15.07
A	GO:0003211	cardiac ventricle formation	-4.17	-3.74	-20.23	-14.68
A	GO:0003207	cardiac chamber formation	-4.03	-3.69	-19.49	-14.52
A	GO:0060536	cartilage morphogenesis	-2.17	-3.64	-20.14	-14.63
A	GO:0048934	peripheral nervous system neuron differentiation	-2.11	-3.31	-20.94	-13.99
A	GO:0048935	peripheral nervous system neuron development	-2.11	-3.31	-20.94	-13.99

Cluster	GO Term	Name	Day 5	Day 8	Day 11	Day 14
A	GO:0003263	cardioblast proliferation	-1.76	-3.20	-20.90	-13.16
A	GO:0003264	regulation of cardioblast proliferation	-1.76	-3.20	-20.90	-13.16
A	GO:0003266	regulation of secondary heart field cardioblast proliferation	-1.76	-3.20	-20.90	-13.16
A	GO:0003228	atrial cardiac muscle tissue development	-1.48	-2.91	-21.93	-15.27
A	GO:0055009	atrial cardiac muscle tissue morphogenesis	-1.48	-2.91	-21.93	-15.27
A	GO:0061032	visceral serous pericardium development	-1.14	-3.23	-21.18	-15.76
A	GO:0010667	negative regulation of cardiac muscle cell apoptosis	-0.97	-3.13	-20.43	-17.84
A	GO:0007512	adult heart development	-0.93	-2.54	-19.51	-16.27
A	GO:0010664	negative regulation of striated muscle cell apoptosis	-0.90	-2.95	-19.35	-16.86
A	GO:0010662	regulation of striated muscle cell apoptosis	-0.83	-2.80	-18.42	-16.15
A	GO:0010665	regulation of cardiac muscle cell apoptosis	-0.83	-2.80	-18.42	-16.15
A	GO:0030198	extracellular matrix organization	-2.80	-13.06	-7.68	-11.41
A	GO:0043062	extracellular structure organization	-2.80	-13.06	-7.68	-11.41
A	GO:0008593	regulation of Notch signaling pathway	-2.46	-20.13	-4.21	-2.42
A	GO:0007219	Notch signaling pathway	-1.59	-11.90	-2.55	-1.80
B	GO:0051151	negative regulation of smooth muscle cell differentiation	-16.90	-2.06	-3.61	-2.11
B	GO:0060395	SMAD protein signal transduction	-12.70	-3.35	-0.80	1.34
B	GO:0010470	regulation of gastrulation	-11.91	-2.99	-0.52	-0.09
B	GO:0051150	regulation of smooth muscle cell differentiation	-11.68	-1.73	-4.13	-2.63
B	GO:0045995	regulation of embryonic development	-9.05	-2.63	-4.88	-2.49
B	GO:0051145	smooth muscle cell differentiation	-9.43	-1.52	-3.66	-3.93
B	GO:0051148	negative regulation of muscle cell differentiation	-9.00	-1.22	-2.60	-2.34
B	GO:0042661	regulation of mesodermal cell fate specification	-9.55	-0.47	-0.30	-0.37
B	GO:0050710	negative regulation of cytokine secretion	-8.43	-0.68	0.49	0.32
B	GO:0001542	ovulation from ovarian follicle	-8.49	-0.31	-0.32	-0.23
B	GO:2000380	regulation of mesoderm development	-7.97	-0.36	-0.24	-0.17
B	GO:0018022	peptidyl-lysine methylation	-8.20	-0.65	-1.46	-0.60
B	GO:0018024	histone-lysine N-methyltransferase activity	-7.88	-0.61	-1.38	-0.53
B	GO:0016278	lysine N-methyltransferase activity	-7.57	-0.62	-1.30	-0.47
B	GO:0016279	protein-lysine N-methyltransferase activity	-7.57	-0.62	-1.30	-0.47
B	GO:0001711	endodermal cell fate commitment	-10.45	2.50	6.35	5.51
B	GO:0035987	endodermal cell differentiation	-10.31	2.32	6.00	5.21
B	GO:0003306	Wnt receptor signaling pathway involved in heart development	-9.51	2.19	6.33	4.75
B	GO:0001714	endodermal cell fate specification	-9.93	2.92	7.19	6.25
B	GO:0003307	regulation of Wnt receptor signaling pathway involved in heart development	-9.51	2.88	8.03	6.06
B	GO:2000043	regulation of cardiac cell fate specification	-8.83	2.67	7.42	5.59

Cluster	GO Term	Name	Day 5	Day 8	Day 11	Day 14
B	GO:0001706	endoderm formation	-12.35	1.36	4.09	3.59
B	GO:0007369	gastrulation	-8.74	2.57	3.13	3.46
B	GO:0007492	endoderm development	-8.70	2.41	3.02	3.27
B	GO:0001704	formation of primary germ layer	-8.02	1.74	2.40	3.08
B	GO:0060911	cardiac cell fate commitment	-7.47	1.93	5.56	4.18
B	GO:0042074	cell migration involved in gastrulation	-8.51	7.32	4.69	5.56
B	GO:0001710	mesodermal cell fate commitment	-5.64	4.74	5.66	5.60
B	GO:0007398	ectoderm development	-4.17	4.91	5.48	4.80
B	GO:0009948	anterior/posterior axis specification	-4.02	4.84	3.40	3.32
C	GO:0008584	male gonad development	-1.78	4.67	3.10	7.32
C	GO:0005160	transforming growth factor beta receptor binding	-1.67	5.70	12.86	15.20
C	GO:0033327	Leydig cell differentiation	-1.87	6.73	7.77	18.40
C	GO:0030238	male sex determination	1.26	5.84	6.56	15.79
C	GO:0060008	Sertoli cell differentiation	2.16	6.96	7.50	17.73
C	GO:0001825	blastocyst formation	-3.71	9.05	10.30	7.81
C	GO:0001824	blastocyst development	-2.05	6.96	7.67	5.92
C	GO:0001829	trophectodermal cell differentiation	-0.88	6.20	9.72	8.51
C	GO:0030325	adrenal gland development	-4.75	4.54	5.28	13.03
C	GO:0060363	cranial suture morphogenesis	-0.61	3.31	5.56	10.84
C	GO:0097094	craniofacial suture morphogenesis	-0.61	3.31	5.56	10.84
C	GO:0007530	sex determination	0.90	4.71	5.18	12.76
C	GO:0045120	pronucleus	3.91	2.19	9.69	5.30
C	GO:0045743	positive regulation of fibroblast growth factor receptor signaling pathway	-0.47	4.84	1.36	1.89
C	GO:0035283	central nervous system segmentation	0.21	5.86	1.64	2.28
C	GO:0035284	brain segmentation	0.21	5.86	1.64	2.28
C	GO:0045103	intermediate filament-based process	0.89	5.19	1.37	1.75
C	GO:0045104	intermediate filament cytoskeleton organization	0.93	5.30	1.41	1.79
C	GO:0048892	lateral line nerve development	1.26	6.61	2.59	1.98
C	GO:0048925	lateral line system development	1.26	6.61	2.59	1.98
C	GO:0048880	sensory system development	1.31	5.75	2.44	1.89
C	GO:0001547	antral ovarian follicle growth	1.59	6.66	4.14	1.37
C	GO:2000194	regulation of female gonad development	1.73	7.13	4.17	1.28
C	GO:0060052	neurofilament cytoskeleton organization	1.62	8.53	2.45	2.36
C	GO:0060053	neurofilament cytoskeleton	1.98	8.27	2.38	2.27
C	GO:0005883	neurofilament	2.22	10.01	2.91	2.76
C	GO:0017158	regulation of calcium ion-dependent exocytosis	0.72	5.71	6.23	3.58
C	GO:0045835	negative regulation of meiosis	2.37	5.58	4.86	3.72
C	GO:0007128	meiotic prophase I	4.39	6.15	5.42	4.87

Cluster	GO Term	Name	Day 5	Day 8	Day 11	Day 14
C	GO:0051324	prophase	4.39	6.15	5.42	4.87
C	GO:0060631	regulation of meiosis I	4.15	5.31	4.32	3.65
C	GO:0048477	oogenesis	4.89	5.14	4.93	3.57
C	GO:0032504	multicellular organism reproduction	4.66	4.88	4.50	5.68
C	GO:0048609	multicellular organismal reproductive process	4.66	4.88	4.50	5.68
C	GO:0048610	cellular process involved in reproduction	5.41	5.08	3.97	5.31
C	GO:0051177	meiotic sister chromatid cohesion	5.95	5.24	5.05	4.74
C	GO:0007276	gamete generation	5.33	5.91	5.47	6.35
C	GO:0019953	sexual reproduction	5.65	6.15	5.79	6.50
C	GO:0007283	spermatogenesis	5.82	6.05	5.68	6.73
C	GO:0048232	male gamete generation	5.82	6.05	5.68	6.73
C	GO:0007127	meiosis I	7.25	7.25	4.47	3.78
C	GO:0051321	meiotic cell cycle	7.15	5.53	3.54	3.08
C	GO:0007126	meiosis	7.33	5.69	3.65	3.18
C	GO:0051327	M phase of meiotic cell cycle	7.33	5.69	3.65	3.18
C	GO:0070193	synaptonemal complex organization	6.29	8.55	6.06	5.04
C	GO:0007130	synaptonemal complex assembly	6.69	9.26	6.55	5.46
C	GO:0000801	central element	7.39	8.48	5.41	4.55
C	GO:0070192	chromosome organization involved in meiosis	9.39	10.50	6.53	5.64
C	GO:0007129	synapsis	9.79	11.16	6.92	5.99
D	GO:0009055	electron carrier activity	7.54	0.76	2.95	1.19
D	GO:0046906	tetrapyrrole binding	7.79	-1.46	2.93	1.27
D	GO:0020037	heme binding	8.34	-1.29	3.07	1.36
D	GO:0072593	reactive oxygen species metabolic process	9.18	1.96	3.84	1.73
D	GO:0051320	S phase	7.48	0.64	0.40	0.78
D	GO:0000084	S phase of mitotic cell cycle	8.41	0.77	0.33	0.79
D	GO:0046686	response to cadmium ion	8.69	1.38	0.42	0.69
D	GO:0045931	positive regulation of mitotic cell cycle	7.73	-0.40	0.35	0.44
D	GO:0006776	vitamin A metabolic process	7.71	-0.45	0.09	0.47
D	GO:0009110	vitamin biosynthetic process	8.29	-0.48	0.26	0.18
D	GO:0006720	isoprenoid metabolic process	8.39	-0.70	0.34	0.41
D	GO:0033261	regulation of S phase	8.43	-0.29	0.26	0.49
D	GO:0008299	isoprenoid biosynthetic process	8.83	-0.40	0.33	0.66
D	GO:0042737	drug catabolic process	8.74	0.09	0.25	-0.07
D	GO:0009410	response to xenobiotic stimulus	8.26	-1.70	0.24	0.62
D	GO:0006805	xenobiotic metabolic process	8.62	-1.81	0.28	0.63
D	GO:0071466	cellular response to xenobiotic stimulus	8.62	-1.81	0.28	0.63
D	GO:0019439	aromatic compound catabolic process	8.72	-1.22	-1.40	0.28
D	GO:0042738	exogenous drug catabolic process	9.16	0.09	0.31	0.31

Cluster	GO Term	Name	Day 5	Day 8	Day 11	Day 14
D	GO:0016725	oxidoreductase activity, acting on CH or CH2 groups	9.56	0.15	0.33	0.30
D	GO:0070989	oxidative demethylation	9.58	0.14	0.34	0.08
D	GO:0042573	retinoic acid metabolic process	9.66	-0.43	0.17	0.34
D	GO:0008395	steroid hydroxylase activity	9.86	-0.25	0.33	0.47
D	GO:0007090	regulation of S phase of mitotic cell cycle	10.00	-0.27	0.30	0.48
D	GO:0042362	fat-soluble vitamin biosynthetic process	10.83	-0.35	0.29	0.42
D	GO:0006721	terpenoid metabolic process	11.08	-0.49	0.16	0.09
D	GO:0006778	porphyrin-containing compound metabolic process	12.28	1.78	0.98	1.12
D	GO:0033013	tetrapyrrole metabolic process	12.28	1.78	0.98	1.12
D	GO:0071276	cellular response to cadmium ion	14.05	2.64	0.99	0.48
D	GO:0019748	secondary metabolic process	12.36	-0.55	-0.32	-0.66
D	GO:0016098	monoterpenoid metabolic process	12.64	0.11	0.62	0.12
D	GO:0070988	demethylation	13.42	0.29	0.48	0.37
D	GO:0017144	drug metabolic process	13.58	0.16	0.41	0.35
D	GO:0009698	phenylpropanoid metabolic process	13.58	-0.22	0.49	0.71
D	GO:0016712	oxidoreductase activity, acting on paired donors, with incorporation or reduction of molecular oxygen	14.78	-0.18	0.50	0.69
D	GO:0042178	xenobiotic catabolic process	14.82	0.12	0.63	0.83
D	GO:0070330	aromatase activity	15.19	-0.21	0.51	0.53
D	GO:0032451	demethylase activity	16.16	0.23	0.60	0.77
D	GO:0035238	vitamin A biosynthetic process	17.45	0.12	0.83	1.02
D	GO:0042904	9-cis-retinoic acid biosynthetic process	17.45	0.12	0.83	1.02
D	GO:0042905	9-cis-retinoic acid metabolic process	17.45	0.12	0.83	1.02
D	GO:0009812	flavonoid metabolic process	18.56	-0.06	0.90	1.12
D	GO:0045750	positive regulation of S phase of mitotic cell cycle	19.07	-0.05	0.95	1.20
D	GO:0016679	oxidoreductase activity, acting on diphenols and related substances as donors	19.42	0.13	1.01	1.24
D	GO:0009804	coumarin metabolic process	19.56	0.04	0.94	1.21
D	GO:0017085	response to insecticide	21.37	0.51	1.36	1.73
D	GO:0042743	hydrogen peroxide metabolic process	16.46	3.10	7.31	3.48
D	GO:0042537	benzene-containing compound metabolic process	11.69	-0.87	-7.16	-4.68
D	GO:0009404	toxin metabolic process	26.96	-0.13	1.39	1.21
D	GO:0050665	hydrogen peroxide biosynthetic process	28.86	0.18	1.62	1.30

Supplemental Table S4: Z-Scores of Top 100 GO Terms AHR-Positive 48 h TCDD vs Control Cells.

Cluster	GO Term	Name	Day 5	Day 8	Day 11	Day 14
A	GO:0060070	canonical Wnt receptor signaling pathway	3.12	-10.99	-1.35	-3.63
A	GO:0016055	Wnt receptor signaling pathway	3.14	-9.24	-0.97	-5.24
A	GO:0030509	BMP signaling pathway	1.91	-9.53	3.03	-3.33
A	GO:0007341	penetration of zona pellucida	0.72	-8.16	0.54	-2.05
A	GO:0030215	semaphorin receptor binding	1.66	-11.18	-6.12	-4.98
A	GO:0000904	cell morphogenesis involved in differentiation	5.04	-10.91	-4.51	-6.70
A	GO:0000902	cell morphogenesis	6.29	-10.11	-4.16	-5.48
A	GO:0032989	cellular component morphogenesis	7.66	-9.88	-4.77	-6.26
A	GO:0002009	morphogenesis of an epithelium	6.53	-9.26	-2.80	-8.96
A	GO:0051642	centrosome localization	2.21	-8.57	-5.26	-4.42
A	GO:0061371	determination of heart left/right asymmetry	1.52	-8.21	-3.90	-6.58
A	GO:0048812	neuron projection morphogenesis	3.52	-9.07	-4.20	-6.04
A	GO:0007411	axon guidance	2.77	-8.72	-4.09	-5.53
A	GO:0048666	neuron development	3.72	-8.23	-3.29	-5.76
A	GO:0035239	tube morphogenesis	4.31	-8.13	-3.19	-5.74
A	GO:0031175	neuron projection development	4.40	-7.98	-3.43	-5.91
A	GO:0048858	cell projection morphogenesis	3.89	-8.05	-3.51	-5.11
A	GO:0032990	cell part morphogenesis	3.76	-8.03	-3.47	-5.01
A	GO:0009887	organ morphogenesis	5.37	-8.49	-3.93	-7.09
A	GO:0009792	embryo development ending in birth or egg hatching	5.51	-8.11	-4.68	-7.16
A	GO:0043009	chordate embryonic development	5.62	-7.88	-4.59	-7.01
A	GO:0070848	response to growth factor stimulus	5.22	-7.78	-3.23	-6.69
A	GO:0005916	fascia adherens	5.45	-4.26	-6.53	-7.31
A	GO:0008284	positive regulation of cell proliferation	7.12	-5.33	-4.82	-6.69
A	GO:0003007	heart morphogenesis	4.59	-5.41	-3.16	-7.96
A	GO:0040011	locomotion	10.42	-9.90	-4.97	-6.47
A	GO:0016477	cell migration	11.58	-9.42	-4.88	-6.00
A	GO:0048646	anatomical structure formation involved in morphogenesis	10.44	-9.45	-4.72	-5.40
A	GO:0048870	cell motility	10.91	-8.90	-4.54	-5.66
A	GO:0051674	localization of cell	10.91	-8.90	-4.54	-5.66
A	GO:0006928	cellular component movement	10.47	-8.33	-4.43	-5.30
A	GO:0005604	basement membrane	14.33	-3.53	-2.46	-6.04
A	GO:0031589	cell-substrate adhesion	11.64	-6.13	-3.51	-3.60
A	GO:0010810	regulation of cell-substrate adhesion	10.84	-5.76	-2.62	-2.46
A	GO:0010811	positive regulation of cell-substrate adhesion	10.03	-5.16	-3.30	-2.52
A	GO:0051094	positive regulation of developmental process	10.11	-5.63	-4.94	-4.73

Cluster	GO Term	Name	Day 5	Day 8	Day 11	Day 14
A	GO:0010740	positive regulation of intracellular protein kinase cascade	9.48	-4.35	-4.69	-4.33
A	GO:0010647	positive regulation of cell communication	9.45	-5.40	-3.77	-4.19
A	GO:0048584	positive regulation of response to stimulus	8.97	-4.88	-3.16	-3.43
A	GO:0044319	wound healing, spreading of cells	9.76	-0.52	-4.07	-2.72
A	GO:0031032	actomyosin structure organization	9.66	-0.91	-4.44	-5.54
A	GO:0030036	actin cytoskeleton organization	10.18	-2.81	-3.22	-3.32
A	GO:0051216	cartilage development	9.20	-2.20	-2.06	-5.03
A	GO:0030485	smooth muscle contractile fiber	7.08	1.31	-10.60	-3.96
A	GO:0048729	tissue morphogenesis	8.25	-10.13	3.10	-8.93
A	GO:0060429	epithelium development	9.76	-7.79	3.24	-9.08
A	GO:0022603	regulation of anatomical structure morphogenesis	7.68	-8.33	4.67	-5.01
A	GO:0007169	transmembrane receptor protein tyrosine kinase signaling pathway	7.30	-7.89	3.43	-1.90
A	GO:0022610	biological adhesion	13.61	-9.47	4.00	-6.45
A	GO:0007155	cell adhesion	13.72	-9.36	4.00	-6.53
A	GO:0072358	cardiovascular system development	11.76	-8.29	8.15	-5.34
A	GO:0072359	circulatory system development	11.76	-8.29	8.15	-5.34
A	GO:0007167	enzyme linked receptor protein signaling pathway	11.12	-9.29	6.15	-2.79
A	GO:0048514	blood vessel morphogenesis	11.35	-7.64	6.18	-4.02
A	GO:0031012	extracellular matrix	13.06	-7.55	3.94	-4.83
A	GO:0030334	regulation of cell migration	12.81	-6.29	4.43	-4.60
A	GO:0051270	regulation of cellular component movement	12.55	-6.28	4.41	-4.29
A	GO:2000145	regulation of cell motility	12.44	-6.09	4.23	-4.41
A	GO:0040012	regulation of locomotion	12.07	-6.68	4.04	-4.40
A	GO:0005578	proteinaceous extracellular matrix	12.19	-6.38	3.27	-4.11
A	GO:0044420	extracellular matrix part	12.44	-3.58	3.86	-4.11
A	GO:0019838	growth factor binding	10.78	-4.57	5.43	-4.01
A	GO:0007160	cell-matrix adhesion	9.69	-5.27	3.32	-2.43
A	GO:0009967	positive regulation of signal transduction	9.76	-5.36	4.16	-4.33
A	GO:0023056	positive regulation of signaling	9.35	-5.42	3.72	-4.11
A	GO:0009986	cell surface	9.42	-7.18	4.15	-3.79
A	GO:0005615	extracellular space	9.01	-6.45	4.28	-3.38
A	GO:0030335	positive regulation of cell migration	13.48	-3.13	2.69	-2.10
A	GO:2000147	positive regulation of cell motility	13.28	-3.10	2.60	-2.03
A	GO:0051272	positive regulation of cellular component movement	13.18	-3.00	2.54	-2.04
A	GO:0040017	positive regulation of locomotion	12.94	-2.92	2.69	-2.00
A	GO:0005178	integrin binding	12.08	-2.62	3.44	-2.05
A	GO:0005912	adherens junction	10.83	-2.64	4.06	-3.57
A	GO:0070161	anchoring junction	10.79	-2.54	4.05	-3.35

Cluster	GO Term	Name	Day 5	Day 8	Day 11	Day 14
A	GO:0042060	wound healing	10.67	-1.25	4.26	-4.27
A	GO:0030198	extracellular matrix organization	10.26	-1.30	3.99	-2.83
A	GO:0043062	extracellular structure organization	10.26	-1.30	3.99	-2.83
A	GO:0009611	response to wounding	9.74	-1.36	3.67	-2.55
A	GO:0051146	striated muscle cell differentiation	9.37	-3.72	3.44	-3.05
A	GO:0055002	striated muscle cell development	9.18	-2.56	3.33	-2.84
A	GO:0048407	platelet-derived growth factor binding	11.26	-1.77	7.19	-7.04
A	GO:0005583	fibrillar collagen	7.14	-2.18	5.27	-7.35
A	GO:0001944	vasculature development	12.55	-8.67	8.83	5.24
A	GO:0001568	blood vessel development	12.72	-7.66	9.10	5.45
A	GO:0001525	angiogenesis	11.55	-5.94	5.19	3.38
A	GO:0030155	regulation of cell adhesion	11.18	-5.73	2.79	1.80
A	GO:0045785	positive regulation of cell adhesion	10.47	-4.55	3.44	2.06
A	GO:0030054	cell junction	9.57	-3.57	3.86	2.28
A	GO:0010595	positive regulation of endothelial cell migration	9.72	-2.31	5.23	4.97
A	GO:0032432	actin filament bundle	13.98	5.78	-3.61	-2.14
A	GO:0001725	stress fiber	12.88	4.07	-3.35	-1.99
A	GO:0042641	actomyosin	12.15	3.71	-3.04	-1.70
A	GO:0015629	actin cytoskeleton	12.21	5.21	-3.75	-3.01
A	GO:0030029	actin filament-based process	10.58	3.17	-3.08	-3.17
A	GO:0004859	phospholipase inhibitor activity	12.60	2.69	0.44	-0.99
A	GO:0005587	collagen type IV	12.16	-0.44	-0.53	-0.58
A	GO:0030935	sheet-forming collagen	12.16	-0.44	-0.53	-0.58
A	GO:0043236	laminin binding	11.21	-1.19	1.30	-1.21
A	GO:0050840	extracellular matrix binding	11.60	1.43	-2.17	-2.05
A	GO:0010755	regulation of plasminogen activation	10.29	0.55	-1.90	-0.33
A	GO:0055102	lipase inhibitor activity	9.82	1.89	0.71	-0.50
A	GO:0030195	negative regulation of blood coagulation	8.92	0.93	-0.38	0.18
A	GO:1900047	negative regulation of hemostasis	8.92	0.93	-0.38	0.18
A	GO:0050819	negative regulation of coagulation	10.14	0.94	1.75	1.50
A	GO:0048661	positive regulation of smooth muscle cell proliferation	9.43	1.14	2.70	1.72
A	GO:0048660	regulation of smooth muscle cell proliferation	8.93	1.46	2.47	1.19
A	GO:0060706	cell differentiation involved in embryonic placenta development	9.19	2.43	4.56	1.58
A	GO:0018149	peptide cross-linking	9.39	-0.29	3.32	-0.67
A	GO:0034330	cell junction organization	9.21	-1.70	3.17	1.56
A	GO:0008092	cytoskeletal protein binding	10.69	5.86	2.71	-1.49
A	GO:0061061	muscle structure development	10.69	5.47	4.42	-3.84
A	GO:0042692	muscle cell differentiation	9.52	3.23	4.19	-2.51
A	GO:0005544	calcium-dependent phospholipid binding	10.78	2.47	1.73	-1.90

Cluster	GO Term	Name	Day 5	Day 8	Day 11	Day 14
A	GO:0003779	actin binding	10.15	3.04	2.28	-2.22
A	GO:0030055	cell-substrate junction	9.06	2.46	1.92	-1.83
A	GO:0005925	focal adhesion	9.02	2.44	2.23	-1.49
A	GO:0005924	cell-substrate adherens junction	8.96	2.26	2.09	-1.69
A	GO:0030855	epithelial cell differentiation	8.21	5.30	2.51	-8.73
A	GO:0030216	keratinocyte differentiation	7.60	4.36	-1.18	-7.95
A	GO:0009913	epidermal cell differentiation	6.78	5.32	-1.41	-7.50
A	GO:0048864	stem cell development	1.87	10.17	3.95	-3.18
A	GO:0019827	stem cell maintenance	1.49	9.97	3.95	-2.79
A	GO:0048863	stem cell differentiation	1.69	8.42	3.89	-3.86
A	GO:0015245	fatty acid transporter activity	3.29	10.16	-1.16	-0.97
A	GO:0050542	icosanoid binding	1.51	11.89	1.47	1.70
A	GO:0050543	icosatetraenoic acid binding	1.51	11.89	1.47	1.70
A	GO:0051782	negative regulation of cell division	-0.55	9.17	-1.04	1.16
A	GO:0070841	inclusion body assembly	0.24	8.68	0.58	0.65
A	GO:0090083	regulation of inclusion body assembly	0.61	8.29	0.44	0.93
A	GO:0000407	pre-autophagosomal structure	1.04	7.89	1.73	1.59
A	GO:0046546	development of primary male sexual characteristics	-3.75	7.92	-1.10	-1.66
A	GO:0001829	trophectodermal cell differentiation	-1.92	8.59	3.61	4.33
A	GO:0001892	embryonic placenta development	8.84	7.20	8.47	5.00
A	GO:0001890	placenta development	8.63	6.83	9.08	6.55
A	GO:0043542	endothelial cell migration	9.11	3.49	6.53	5.32
A	GO:0010594	regulation of endothelial cell migration	8.83	2.96	7.19	6.00
A	GO:0045662	negative regulation of myoblast differentiation	3.09	4.56	7.20	6.28
A	GO:0060711	labyrinthine layer development	4.03	4.84	6.95	4.32
A	GO:0032878	regulation of establishment or maintenance of cell polarity	1.00	2.39	7.46	5.95
A	GO:0015665	alcohol transmembrane transporter activity	0.97	3.28	6.99	6.04
A	GO:0045713	low-density lipoprotein particle receptor biosynthetic process	2.14	1.50	7.04	7.64
A	GO:0010871	negative regulation of receptor biosynthetic process	1.90	1.64	6.40	6.91
A	GO:0016936	galactoside binding	4.23	0.94	6.40	5.73
A	GO:0060712	spongiotrophoblast layer development	2.75	1.35	6.36	3.48
A	GO:0009404	toxin metabolic process	5.79	2.70	3.68	6.93
A	GO:0048185	activin binding	-0.14	-2.68	7.08	4.24
A	GO:0030033	microvillus assembly	0.66	-0.88	6.45	5.34
A	GO:0019885	antigen processing and presentation of endogenous peptide antigen via MHC class I	0.59	-3.38	3.33	8.95
A	GO:0002483	antigen processing and presentation of endogenous peptide antigen	-0.17	-3.00	2.87	7.78
A	GO:0019883	antigen processing and presentation of endogenous antigen	0.15	-2.73	2.64	7.26

Cluster	GO Term	Name	Day 5	Day 8	Day 11	Day 14
A	GO:0042573	retinoic acid metabolic process	2.23	0.81	3.11	8.85
A	GO:0035238	vitamin A biosynthetic process	3.30	1.00	2.45	8.34
A	GO:0042904	9-cis-retinoic acid biosynthetic process	3.30	1.00	2.45	8.34
A	GO:0042905	9-cis-retinoic acid metabolic process	3.30	1.00	2.45	8.34
A	GO:0034754	cellular hormone metabolic process	1.79	-1.11	4.28	7.52
A	GO:0004030	aldehyde dehydrogenase [NAD(P)+] activity	0.64	1.16	2.36	7.14
A	GO:0006776	vitamin A metabolic process	1.33	0.85	2.58	7.08
A	GO:0001523	retinoid metabolic process	1.05	0.90	2.37	6.61
A	GO:0016101	diterpenoid metabolic process	1.05	0.90	2.37	6.61
A	GO:0060900	embryonic camera-type eye formation	-0.67	-0.57	2.47	6.96
A	GO:0042612	MHC class I protein complex	0.61	-1.51	2.27	6.60
B	GO:0048864	stem cell development	1.87	10.17	3.95	-3.18
B	GO:0019827	stem cell maintenance	1.49	9.97	3.95	-2.79
B	GO:0048863	stem cell differentiation	1.69	8.42	3.89	-3.86
B	GO:0015245	fatty acid transporter activity	3.29	10.16	-1.16	-0.97
B	GO:0050542	icosanoid binding	1.51	11.89	1.47	1.70
B	GO:0050543	icosatetraenoic acid binding	1.51	11.89	1.47	1.70
B	GO:0051782	negative regulation of cell division	-0.55	9.17	-1.04	1.16
B	GO:0070841	inclusion body assembly	0.24	8.68	0.58	0.65
B	GO:0090083	regulation of inclusion body assembly	0.61	8.29	0.44	0.93
B	GO:0000407	pre-autophagosomal structure	1.04	7.89	1.73	1.59
B	GO:0046546	development of primary male sexual characteristics	-3.75	7.92	-1.10	-1.66
B	GO:0001829	trophectodermal cell differentiation	-1.92	8.59	3.61	4.33
B	GO:0001892	embryonic placenta development	8.84	7.20	8.47	5.00
B	GO:0001890	placenta development	8.63	6.83	9.08	6.55
B	GO:0043542	endothelial cell migration	9.11	3.49	6.53	5.32
B	GO:0010594	regulation of endothelial cell migration	8.83	2.96	7.19	6.00
B	GO:0045662	negative regulation of myoblast differentiation	3.09	4.56	7.20	6.28
B	GO:0060711	labyrinthine layer development	4.03	4.84	6.95	4.32
B	GO:0032878	regulation of establishment or maintenance of cell polarity	1.00	2.39	7.46	5.95
B	GO:0015665	alcohol transmembrane transporter activity	0.97	3.28	6.99	6.04
B	GO:0045713	low-density lipoprotein particle receptor biosynthetic process	2.14	1.50	7.04	7.64
B	GO:0010871	negative regulation of receptor biosynthetic process	1.90	1.64	6.40	6.91
B	GO:0016936	galactoside binding	4.23	0.94	6.40	5.73
B	GO:0060712	spongiotrophoblast layer development	2.75	1.35	6.36	3.48
B	GO:0009404	toxin metabolic process	5.79	2.70	3.68	6.93
B	GO:0048185	activin binding	-0.14	-2.68	7.08	4.24
B	GO:0030033	microvillus assembly	0.66	-0.88	6.45	5.34

Cluster	GO Term	Name	Day 5	Day 8	Day 11	Day 14
B	GO:0019885	antigen processing and presentation of endogenous peptide antigen via MHC class I	0.59	-3.38	3.33	8.95
B	GO:0002483	antigen processing and presentation of endogenous peptide antigen	-0.17	-3.00	2.87	7.78
B	GO:0019883	antigen processing and presentation of endogenous antigen	0.15	-2.73	2.64	7.26
B	GO:0042573	retinoic acid metabolic process	2.23	0.81	3.11	8.85
B	GO:0035238	vitamin A biosynthetic process	3.30	1.00	2.45	8.34
B	GO:0042904	9-cis-retinoic acid biosynthetic process	3.30	1.00	2.45	8.34
B	GO:0042905	9-cis-retinoic acid metabolic process	3.30	1.00	2.45	8.34
B	GO:0034754	cellular hormone metabolic process	1.79	-1.11	4.28	7.52
B	GO:0004030	aldehyde dehydrogenase [NAD(P)+] activity	0.64	1.16	2.36	7.14
B	GO:0006776	vitamin A metabolic process	1.33	0.85	2.58	7.08
B	GO:0001523	retinoid metabolic process	1.05	0.90	2.37	6.61
B	GO:0016101	diterpenoid metabolic process	1.05	0.90	2.37	6.61
B	GO:0060900	embryonic camera-type eye formation	-0.67	-0.57	2.47	6.96
B	GO:0042612	MHC class I protein complex	0.61	-1.51	2.27	6.60
C	GO:0004551	nucleotide diphosphatase activity	-11.22	-9.96	-9.57	-6.96
C	GO:0010424	DNA methylation on cytosine within a CG sequence	-15.05	-7.46	-18.57	-13.84
C	GO:0032776	DNA methylation on cytosine	-15.05	-7.46	-18.57	-13.84
C	GO:0006346	methylation-dependent chromatin silencing	-12.80	-6.34	-15.28	-11.72
C	GO:0045322	unmethylated CpG binding	-12.01	-6.12	-14.46	-10.84
C	GO:0046498	S-adenosylhomocysteine metabolic process	-11.46	-6.10	-13.27	-10.24
C	GO:0023019	signal transduction involved in regulation of gene expression	-5.84	-11.28	0.37	-1.37
C	GO:0007509	mesoderm migration involved in gastrulation	-0.81	-13.88	-0.58	-2.27
C	GO:0001839	neural plate morphogenesis	-0.62	-11.88	-0.57	-2.96
C	GO:0001840	neural plate development	-1.03	-10.64	-0.64	-2.75
C	GO:0060379	cardiac muscle cell myoblast differentiation	-0.55	-10.21	-0.35	-2.59
C	GO:0004768	stearoyl-CoA 9-desaturase activity	-1.34	-10.14	-3.24	-3.29
C	GO:0016215	acyl-CoA desaturase activity	-1.21	-9.94	-4.02	-4.45
C	GO:0016717	oxidoreductase activity, acting on paired donors, with oxidation of a pair of donors	-0.71	-8.26	-3.19	-3.55
C	GO:0021915	neural tube development	-1.96	-8.49	-1.12	-3.85
C	GO:0001707	mesoderm formation	-3.59	-10.89	-3.06	-2.86
C	GO:0001704	formation of primary germ layer	-3.44	-10.57	-2.90	-2.72
C	GO:0048332	mesoderm morphogenesis	-3.44	-10.41	-2.82	-2.65
C	GO:0048667	cell morphogenesis involved in neuron differentiation	-2.90	-8.87	-3.29	-4.95
C	GO:0007409	axonogenesis	-3.20	-8.68	-3.28	-4.90
C	GO:0007498	mesoderm development	-4.75	-9.21	-3.26	-3.89
C	GO:0005003	ephrin receptor activity	-4.18	-8.43	-3.18	-2.89
C	GO:0009952	anterior/posterior pattern specification	-4.34	-8.01	-2.39	-3.13

Cluster	GO Term	Name	Day 5	Day 8	Day 11	Day 14
C	GO:0042758	long-chain fatty acid catabolic process	-4.28	-9.28	-1.62	-1.34
C	GO:0001561	fatty acid alpha-oxidation	-4.65	-9.25	-1.72	-1.32
C	GO:0031957	very long-chain fatty acid-CoA ligase activity	-3.94	-8.66	-1.44	-1.17
C	GO:0035567	non-canonical Wnt receptor signaling pathway	-3.19	-8.66	-1.20	-3.15
C	GO:0060346	bone trabecula formation	-4.16	-8.54	-1.19	-2.60
C	GO:0061430	bone trabecula morphogenesis	-4.16	-8.54	-1.19	-2.60
C	GO:0017147	Wnt-protein binding	-3.99	-8.08	-1.30	-2.52
C	GO:0035121	tail morphogenesis	-4.82	-13.71	-4.30	-4.47
C	GO:0007369	gastrulation	-3.11	-11.53	-4.92	-4.35
C	GO:0042074	cell migration involved in gastrulation	-7.23	-11.32	-7.34	-5.13
C	GO:0030903	notochord development	-4.61	-11.89	-6.02	-7.05
C	GO:0044344	cellular response to fibroblast growth factor stimulus	-6.26	-10.77	-4.94	-6.93
C	GO:0071774	response to fibroblast growth factor stimulus	-6.26	-10.77	-4.94	-6.93
C	GO:0009948	anterior/posterior axis specification	-6.00	-9.91	-5.70	-5.56
C	GO:0048598	embryonic morphogenesis	-3.36	-8.47	-4.42	-6.80
C	GO:0022008	neurogenesis	-5.15	-8.50	-4.98	-6.46
C	GO:0030182	neuron differentiation	-4.35	-8.63	-4.71	-6.76
C	GO:0048699	generation of neurons	-4.42	-8.17	-4.40	-6.31
C	GO:0008543	fibroblast growth factor receptor signaling pathway	-6.75	-8.18	-4.76	-6.01
C	GO:0035282	segmentation	-7.96	-10.35	-3.69	-3.98
C	GO:0001756	somitogenesis	-7.17	-9.72	-3.79	-3.41
C	GO:0061053	somite development	-6.78	-9.20	-3.82	-4.43
C	GO:0046475	glycerophospholipid catabolic process	-9.97	-7.03	-4.67	-3.18
C	GO:0010508	positive regulation of autophagy	-6.92	-8.38	-7.97	-3.04
C	GO:0014054	positive regulation of gamma-aminobutyric acid secretion	-1.27	-9.57	-10.69	-11.48
C	GO:0051957	positive regulation of amino acid transport	-1.27	-9.57	-10.69	-11.48
C	GO:0014050	negative regulation of glutamate secretion	-1.06	-8.51	-8.31	-8.98
C	GO:0014052	regulation of gamma-aminobutyric acid secretion	-1.15	-8.40	-9.23	-10.28
C	GO:0046942	carboxylic acid transport	-2.20	-9.42	-7.15	-6.66
C	GO:0015849	organic acid transport	-2.15	-9.34	-7.08	-6.60
C	GO:0046943	carboxylic acid transmembrane transporter activity	-1.73	-8.31	-6.86	-6.12
C	GO:0005342	organic acid transmembrane transporter activity	-1.65	-8.18	-6.74	-6.02
C	GO:0015837	amine transport	-1.61	-7.49	-6.38	-5.70
C	GO:0006865	amino acid transport	-1.18	-8.83	-8.63	-7.93
C	GO:0015171	amino acid transmembrane transporter activity	-1.63	-8.36	-8.69	-7.31
C	GO:0003333	amino acid transmembrane transport	-1.73	-7.66	-8.34	-7.26
C	GO:0071705	nitrogen compound transport	-1.27	-7.61	-7.87	-7.60
C	GO:0005275	amine transmembrane transporter activity	-1.27	-7.75	-7.66	-6.43

Cluster	GO Term	Name	Day 5	Day 8	Day 11	Day 14
C	GO:0032892	positive regulation of organic acid transport	-2.63	-6.35	-6.98	-6.90
C	GO:0043403	skeletal muscle tissue regeneration	-2.50	-4.18	-6.87	-7.26
C	GO:0014051	gamma-aminobutyric acid secretion	-0.72	-6.86	-7.37	-8.50
C	GO:0015812	gamma-aminobutyric acid transport	-1.05	-6.32	-6.76	-7.77
C	GO:0051955	regulation of amino acid transport	-0.44	-6.06	-6.57	-7.40
C	GO:0015179	L-amino acid transmembrane transporter activity	-0.80	-5.46	-6.18	-6.03
C	GO:0014829	vascular smooth muscle contraction	3.98	-0.84	-7.92	-4.02
C	GO:0043123	positive regulation of I-kappaB kinase/NF-kappaB cascade	2.68	-3.93	-6.90	-5.93
C	GO:0005865	striated muscle thin filament	4.94	-1.50	-6.54	-7.25
C	GO:0060174	limb bud formation	-0.79	-5.69	-8.98	-12.51
C	GO:0007512	adult heart development	-0.53	-3.76	-7.00	-11.02
C	GO:0014866	skeletal myofibril assembly	2.44	-2.31	-7.66	-9.54
C	GO:0030240	skeletal muscle thin filament assembly	2.44	-2.31	-7.66	-9.54
C	GO:0045844	positive regulation of striated muscle tissue development	1.99	-3.09	-7.10	-10.02
C	GO:0048636	positive regulation of muscle organ development	1.99	-3.09	-7.10	-10.02
C	GO:0072530	purine-containing compound transmembrane transport	-0.60	-2.63	-7.78	-8.60
C	GO:0005243	gap junction channel activity	-0.62	-3.28	-7.35	-8.49
C	GO:0015174	basic amino acid transmembrane transporter activity	-0.50	-4.15	-6.85	-8.97
C	GO:0090162	establishment of epithelial cell polarity	0.88	-4.15	-6.09	-8.66
C	GO:0002070	epithelial cell maturation	-0.39	-2.77	-6.16	-7.39
C	GO:0015809	arginine transport	0.36	-3.28	-6.08	-7.78
C	GO:0015802	basic amino acid transport	0.08	-2.99	-5.03	-6.70
C	GO:0001502	cartilage condensation	4.85	-1.52	-4.66	-11.13
C	GO:0005720	nuclear heterochromatin	-9.51	-3.77	-11.86	-8.55
C	GO:0006349	regulation of gene expression by genetic imprinting	-8.11	-3.90	-10.79	-8.25
C	GO:0000792	heterochromatin	-9.01	-3.09	-9.57	-6.42
C	GO:0051571	positive regulation of histone H3-K4 methylation	-8.96	-5.46	-11.29	-11.59
C	GO:0051573	negative regulation of histone H3-K9 methylation	-7.24	-5.45	-10.58	-11.50
C	GO:0051570	regulation of histone H3-K9 methylation	-7.60	-4.52	-10.57	-10.28
C	GO:0031062	positive regulation of histone methylation	-7.25	-4.26	-10.06	-9.74
C	GO:0010216	maintenance of DNA methylation	-8.97	-2.29	-8.81	-2.65
C	GO:0071514	genetic imprinting	-6.60	-3.05	-8.82	-6.68
C	GO:0006342	chromatin silencing	-7.12	-3.05	-8.37	-6.24
C	GO:0045814	negative regulation of gene expression, epigenetic	-6.77	-2.75	-7.85	-5.80
C	GO:0031061	negative regulation of histone methylation	-5.13	-3.78	-8.62	-8.75
C	GO:0031503	protein complex localization	-5.92	-3.77	-8.06	-8.24

Cluster	GO Term	Name	Day 5	Day 8	Day 11	Day 14
C	GO:0051567	histone H3-K9 methylation	-6.35	-3.12	-7.99	-7.62
C	GO:0051569	regulation of histone H3-K4 methylation	-5.56	-3.52	-7.26	-7.56
C	GO:0043200	response to amino acid stimulus	-5.79	-6.74	-7.79	-6.22
C	GO:0071230	cellular response to amino acid stimulus	-6.23	-5.66	-7.85	-5.58
C	GO:0071418	cellular response to amine stimulus	-5.80	-5.31	-7.52	-5.86
C	GO:0071417	cellular response to organic nitrogen	-5.67	-5.20	-7.38	-5.75
C	GO:0071229	cellular response to acid	-6.57	-5.52	-7.06	-5.26
C	GO:0046128	purine ribonucleoside metabolic process	-6.60	-4.27	-7.22	-5.75
C	GO:0042278	purine nucleoside metabolic process	-6.43	-3.92	-6.81	-5.37
C	GO:0031054	pre-miRNA processing	-2.78	-1.35	-8.07	-5.92
C	GO:0034661	ncRNA catabolic process	-3.55	-1.36	-7.22	-4.48
C	GO:0035198	miRNA binding	-3.01	-1.94	-6.81	-4.99
C	GO:0035196	production of miRNAs involved in gene silencing by miRNA	-2.72	-1.00	-7.25	-5.56
C	GO:0031050	dsRNA fragmentation	-2.57	-0.88	-6.86	-5.28
C	GO:0070918	production of small RNA involved in gene silencing by RNA	-2.57	-0.88	-6.86	-5.28
C	GO:0016458	gene silencing	-5.24	-1.69	-7.68	-5.58
C	GO:0006305	DNA alkylation	-6.06	-2.24	-6.68	-4.72
C	GO:0006306	DNA methylation	-6.06	-2.24	-6.68	-4.72
C	GO:0040029	regulation of gene expression, epigenetic	-4.55	-0.77	-6.33	-4.42
C	GO:0031060	regulation of histone methylation	-4.70	-2.75	-6.65	-6.52
C	GO:0031641	regulation of myelination	-0.92	-4.32	-7.08	-4.68
C	GO:0022010	central nervous system myelination	-0.71	-3.14	-6.88	-3.45
C	GO:0032291	axon ensheathment in central nervous system	-0.71	-3.14	-6.88	-3.45
C	GO:0045109	intermediate filament organization	-1.28	-2.59	-0.61	-15.52
C	GO:0045104	intermediate filament cytoskeleton organization	-2.37	-2.03	-3.08	-14.77
C	GO:0045103	intermediate filament-based process	-2.26	-1.94	-2.97	-14.48
C	GO:0000307	cyclin-dependent protein kinase holoenzyme complex	-3.48	1.87	-3.07	-9.47
C	GO:0071772	response to BMP stimulus	1.28	1.43	1.45	-11.36
C	GO:0071773	cellular response to BMP stimulus	1.28	1.43	1.45	-11.36
C	GO:0060272	embryonic skeletal joint morphogenesis	1.35	0.56	0.41	-9.56
C	GO:0031424	keratinization	1.83	-0.91	0.55	-9.72
C	GO:0072498	embryonic skeletal joint development	1.05	-0.50	0.27	-9.04
C	GO:0060351	cartilage development involved in endochondral bone morphogenesis	3.05	-2.65	-1.27	-7.99
C	GO:0045737	positive regulation of cyclin-dependent protein kinase activity	4.27	-1.62	-0.89	-7.45
C	GO:0030836	positive regulation of actin filament depolymerization	0.19	-0.24	-3.64	-6.67
C	GO:0005179	hormone activity	1.71	-1.94	14.54	15.12

Supplemental Table S5. Expression levels of transcription factors that show differential expression between TCDD-treated AHR-positive cells and controls (Log2Fold change)

geneid	symbol	name	Day 5	Day 8	Day 11	Day 14
11910	*Atf3*	activating transcription factor 3	0.37	0.22	2.51	0.44
223922	*Atf7*	activating transcription factor 7	0.21	0.28	1.01	0.23
12053	*Bcl6*	B-cell leukemia/lymphoma 6	0.34	0.68	2.62	0.77
12578	*Cdkn2a*	cyclin-dependent kinase inhibitor 2A	0.54	0.22	1.78	0.41
12606	*Cebpa*	CCAAT/enhancer binding protein (C/EBP), alpha	-0.14	0.33	1.09	1.35
12705	*Cited1*	Cbp/p300-interacting transactivator with Glu/Asp-rich carboxy-terminal domain 1	0.08	1.35	2.06	1.84
17684	*Cited2*	Cbp/p300-interacting transactivator, with Glu/Asp-rich carboxy-terminal domain, 2	0.08	0.79	1.35	0.81
12914	*Crebbp*	CREB binding protein	0.02	0.19	0.06	-0.03
12387	*Ctnnb1*	catenin (cadherin associated protein), beta 1	0.00	0.08	-0.16	0.14
13393	*Dlx3*	distal-less homeobox 3	0.48	1.04	2.99	1.61
13819	*Epas1*	endothelial PAS domain protein 1	0.43	0.11	0.17	-0.06
23871	*Ets1*	E26 avian leukemia oncogene 1, 5' domain	0.45	-0.06	1.79	0.00
15376	*Foxa2*	forkhead box A2	-0.50	-0.10	0.91	0.21
17300	*Foxc1*	forkhead box C1	0.53	0.26	1.28	1.48
14234	*Foxc2*	forkhead box C2	1.81	1.64	1.14	2.21
14106	*Foxh1*	forkhead box H1	-0.72	-0.82	-1.01	-1.25
108655	*Foxp1*	forkhead box P1	-0.89	-0.53	-1.11	-0.90
14461	*Gata2*	GATA binding protein 2	0.73	1.41	1.65	2.09
14462	*Gata3*	GATA binding protein 3	1.34	0.69	2.28	1.47
14463	*Gata4*	GATA binding protein 4	0.59	0.19	0.84	0.69
14465	*Gata6*	GATA binding protein 6	0.90	0.43	1.91	0.94
14472	*Gbx2*	gastrulation brain homeobox 2	0.08	-0.94	-0.20	-1.07
14634	*Gli3*	GLI-Kruppel family member GLI3	-0.24	0.06	0.29	0.20
15110	*Hand1*	heart and neural crest derivatives expressed transcript 1	0.03	1.08	4.32	1.60
15111	*Hand2*	heart and neural crest derivatives expressed transcript 2	1.14	1.37	-0.86	1.55
433759	*Hdac1*	histone deacetylase 1	-0.07	-0.01	-0.29	0.00
15214	*Hey2*	hairy/enhancer-of-split related with YRPW motif 2	-0.33	0.58	0.57	0.30
21405	*Hnf1a*	HNF1 homeobox A	0.90	0.22	-0.25	0.13
74318	*Hopx*	HOP homeobox	-1.73	-0.42	-1.46	-0.35
15394	*Hoxa1*	homeobox A1	0.59	0.77	1.76	1.08
15395	*Hoxa10*	homeobox A10	1.22	0.93	1.19	0.44
15400	*Hoxa3*	homeobox A3	0.32	0.78	2.24	0.66
15410	*Hoxb3*	homeobox B3	-1.18	0.60	-1.56	0.54
15413	*Hoxb5*	homeobox B5	0.61	0.87	-2.91	-0.54
15430	*Hoxd10*	homeobox D10	1.74	0.68	-0.31	0.06

geneid	symbol	name	Day 5	Day 8	Day 11	Day 14
50916	*Irx4*	Iroquois related homeobox 4 (Drosophila)	0.35	-0.01	3.37	1.97
16392	*Isl1*	ISL1 transcription factor, LIM/homeodomain	0.24	0.32	0.70	1.07
16476	*Jun*	Jun oncogene	0.46	0.20	2.16	0.24
16598	*Klf2*	Kruppel-like factor 2 (lung)	1.06	0.38	0.65	0.60
12224	*Klf5*	Kruppel-like factor 5	0.32	0.11	1.53	0.28
16842	*Lef1*	lymphoid enhancer binding factor 1	-2.34	-0.25	-2.59	-0.86
17260	*Mef2c*	myocyte enhancer factor 2C	-1.39	0.47	-2.19	0.23
17268	*Meis1*	Meis homeobox 1	0.33	0.56	2.85	1.28
17292	*Mesp1*	mesoderm posterior 1	-1.05	0.08	-0.37	0.39
17293	*Mesp2*	mesoderm posterior 2	1.21	0.69	-0.46	0.44
17387	*Mmp14*	matrix metallopeptidase 14 (membrane-inserted)	-0.32	-0.15	2.52	0.15
17701	*Msx1*	homeobox, msh-like 1	-2.06	0.77	-2.26	-0.48
17869	*Myc*	myelocytomatosis oncogene	-0.83	-0.31	-0.54	-0.58
18109	*Mycn*	v-myc myelocytomatosis viral related oncogene, neuroblastoma derived (avian)	-0.41	-0.61	-1.39	-1.01
71950	*Nanog*	Nanog homeobox	0.90	0.61	0.40	0.73
18091	*Nkx2-5*	NK2 transcription factor related, locus 5 (Drosophila)	0.10	0.04	0.06	0.81
18092	*Nkx2-6*	NK2 transcription factor related, locus 6 (Drosophila)	-1.15	-0.14	-0.78	-0.11
11819	*Nr2f2*	nuclear receptor subfamily 2, group F, member 2	2.06	0.05	3.14	0.83
15370	*Nr4a1*	nuclear receptor subfamily 4, group A, member 1	-0.08	0.01	1.13	0.37
26423	*Nr5a1*	nuclear receptor subfamily 5, group A, member 1	-0.05	-0.62	0.17	0.93
26424	*Nr5a2*	nuclear receptor subfamily 5, group A, member 2	1.18	0.21	1.28	0.38
18505	*Pax3*	paired box gene 3	-0.21	-0.23	1.36	-0.68
18508	*Pax6*	paired box gene 6	-0.54	-0.26	-0.77	-0.50
18741	*Pitx2*	paired-like homeodomain transcription factor 2	-0.41	0.30	-1.07	0.05
18999	*Pou5f1*	POU domain, class 5, transcription factor 1	-0.57	-0.18	-0.79	-0.32
19130	*Prox1*	prospero-related homeobox 1	-0.01	0.28	0.83	0.14
18933	*Prrx1*	paired related homeobox 1	-0.05	0.29	1.22	-0.05
218772	*Rarb*	retinoic acid receptor, beta	0.71	0.42	2.73	1.59
19411	*Rarg*	retinoic acid receptor, gamma	-0.23	-0.16	-0.47	-0.38
19697	*Rela*	v-rel reticuloendotheliosis viral oncogene homolog A (avian)	0.24	0.08	1.21	0.13
12394	*Runx1*	runt related transcription factor 1	-0.05	-0.19	2.53	0.04
12393	*Runx2*	runt related transcription factor 2	-0.04	-0.35	-0.44	-1.21
20429	*Shox2*	short stature homeobox 2	0.07	0.69	0.60	1.46
17127	*Smad3*	MAD homolog 3 (Drosophila)	0.20	0.21	1.30	0.65
17128	*Smad4*	MAD homolog 4 (Drosophila)	0.22	0.00	0.84	-0.04
20665	*Sox10*	SRY-box containing gene 10	-0.04	0.00	-1.41	0.15
20671	*Sox17*	SRY-box containing gene 17	1.11	0.14	1.08	0.89
20672	*Sox18*	SRY-box containing gene 18	-0.26	0.17	-2.47	-0.11

geneid	symbol	name	Day 5	Day 8	Day 11	Day 14
20674	*Sox2*	SRY-box containing gene 2	0.59	0.12	-0.25	0.10
20677	*Sox4*	SRY-box containing gene 4	-0.42	-0.09	-0.12	-0.19
20678	*Sox5*	SRY-box containing gene 5	0.93	-0.17	0.26	1.09
20679	*Sox6*	SRY-box containing gene 6	-0.19	-0.45	1.04	-0.36
20680	*Sox7*	SRY-box containing gene 7	0.88	0.11	0.13	-0.42
20681	*Sox8*	SRY-box containing gene 8	0.68	0.68	-0.72	0.17
20682	*Sox9*	SRY-box containing gene 9	0.09	-0.23	0.94	1.38
20848	*Stat3*	signal transducer and activator of transcription 3	0.35	0.27	1.25	0.37
20850	*Stat5a*	signal transducer and activator of transcription 5A	0.72	0.52	1.35	0.95
20997	*T*	brachyury	-3.14	-1.26	-0.35	-0.29
21349	*Tal1*	T-cell acute lymphocytic leukemia 1	-1.75	1.09	-3.80	-1.96
21380	*Tbx1*	T-box 1	0.89	-0.17	0.78	0.39
76365	*Tbx18*	T-box18	1.05	0.35	-0.29	0.22
21385	*Tbx2*	T-box 2	0.26	0.74	0.83	1.13
57246	*Tbx20*	T-box 20	-0.40	0.29	0.25	-0.45
21386	*Tbx3*	T-box 3	1.59	0.84	2.22	1.29
21388	*Tbx5*	T-box 5	0.48	-0.09	-0.12	-0.46
21414	*Tcf7*	transcription factor 7, T-cell specific	-0.19	-0.03	-0.84	-0.10
21415	*Tcf7l1*	transcription factor 7-like 1 (T-cell specific, HMG box)	0.00	0.02	-0.51	0.17
21416	*Tcf7l2*	transcription factor 7-like 2, T-cell specific, HMG-box	-0.09	0.22	0.61	0.70
21676	*Tead1*	TEA domain family member 1	0.06	0.07	1.30	0.25
21679	*Tead4*	TEA domain family member 4	0.43	0.19	2.29	0.46
22059	*Trp53*	transformation related protein 53	0.05	-0.09	-0.26	-0.18
22431	*Wt1*	Wilms tumor 1 homolog	-1.24	-1.06	-3.08	-1.15
22773	*Zic3*	zinc finger protein of the cerebellum 3	-0.23	-0.39	-0.43	-0.62
77128	*A930001N09Rik*	RIKEN cDNA A930001N09 gene	0.32	0.32	0.36	0.26
56321	*Aatf*	apoptosis antagonizing transcription factor	0.12	0.05	-0.39	-0.18
11538	*Adnp*	activity-dependent neuroprotective protein	0.09	0.11	0.03	0.13
240442	*Adnp2*	ADNP homeobox 2	-0.07	0.32	-0.16	0.19
17355	*Aff1*	AF4/FMR2 family, member 1	-0.13	-0.31	0.60	-0.47
16764	*Aff3*	AF4/FMR2 family, member 3	-0.58	-0.18	-1.25	-0.40
226747	*Ahctf1*	AT hook containing transcription factor 1	0.02	0.12	-0.25	-0.01
11622	*Ahr*	aryl-hydrocarbon receptor	-0.32	-0.40	-0.52	-0.44
216285	*Alx1*	ALX homeobox 1	0.29	0.78	-0.72	-0.23
11694	*Alx3*	aristaless-like homeobox 3	0.88	1.72	0.75	0.33
11695	*Alx4*	aristaless-like homeobox 4	-0.39	0.08	0.49	1.08
11835	*Ar*	androgen receptor	-1.41	0.27	-3.25	-0.71
13496	*Arid3a*	AT rich interactive domain 3A (BRIGHT-like)	0.51	0.69	2.38	1.01

geneid	symbol	name	Day 5	Day 8	Day 11	Day 14
11863	*Arnt*	aryl hydrocarbon receptor nuclear translocator	0.09	0.23	-0.18	0.18
11864	*Arnt2*	aryl hydrocarbon receptor nuclear translocator 2	-0.15	-0.28	-0.89	-0.73
11865	*Arntl*	aryl hydrocarbon receptor nuclear translocator-like	-0.13	0.25	0.01	0.13
272322	*Arntl2*	aryl hydrocarbon receptor nuclear translocator-like 2	-0.36	0.28	1.31	0.95
11878	*Arx*	aristaless related homeobox	-0.17	0.27	-1.13	-0.09
17172	*Ascl1*	achaete-scute complex homolog 1 (Drosophila)	-0.08	1.48	-1.45	-0.44
17173	*Ascl2*	achaete-scute complex homolog 2 (Drosophila)	0.30	0.17	-0.99	0.42
11908	*Atf1*	activating transcription factor 1	0.01	-0.01	-0.43	-0.24
11909	*Atf2*	activating transcription factor 2	-0.09	0.00	0.02	-0.18
11911	*Atf4*	activating transcription factor 4	0.36	0.01	-0.13	-0.31
107503	*Atf5*	activating transcription factor 5	0.79	0.57	0.38	0.09
12915	*Atf6b*	activating transcription factor 6 beta	-0.74	-0.34	-0.38	-0.37
330361	*AW146020*	expressed sequence AW146020	-0.06	-0.21	-0.15	0.09
12013	*Bach1*	BTB and CNC homology 1	0.10	0.20	0.08	0.11
12014	*Bach2*	BTB and CNC homology 2	0.17	0.16	1.69	0.17
54422	*Barhl1*	BarH-like 1 (Drosophila)	0.14	-0.73	0.30	-0.92
104382	*Barhl2*	BarH-like 2 (Drosophila)	-0.04	0.13	-0.06	-0.75
12022	*Barx1*	BarH-like homeobox 1	0.67	0.21	0.83	0.97
12023	*Barx2*	BarH-like homeobox 2	0.67	0.41	-0.45	1.83
53314	*Batf*	basic leucine zipper transcription factor, ATF-like	-0.94	-0.29	-1.13	0.22
74481	*Batf2*	basic leucine zipper transcription factor, ATF-like 2	0.41	0.44	1.15	0.06
381319	*Batf3*	basic leucine zipper transcription factor, ATF-like 3	-0.69	1.21	-0.37	2.37
12051	*Bcl3*	B-cell leukemia/lymphoma 3	0.35	0.68	1.68	1.04
107771	*Bmyc*	brain expressed myelocytomatosis oncogene	-0.17	0.31	-0.20	0.61
244813	*Bsx*	brain specific homeobox	-0.19	-0.47	0.82	0.17
241066	*Carf*	calcium response factor	0.04	0.03	0.44	0.35
12396	*Cbfa2t2*	core-binding factor, runt domain, alpha subunit 2, translocated to, 2 (human)	0.31	0.17	-0.11	0.25
12398	*Cbfa2t3*	core-binding factor, runt domain, alpha subunit 2, translocated to, 3 (human)	0.95	0.85	1.61	1.53
12590	*Cdx1*	caudal type homeobox 1	0.49	1.03	1.19	0.82
12591	*Cdx2*	caudal type homeobox 2	0.13	1.63	-0.24	2.14
12592	*Cdx4*	caudal type homeobox 4	-2.68	-0.48	-2.61	-1.13
12608	*Cebpb*	CCAAT/enhancer binding protein (C/EBP), beta	0.56	0.71	0.68	1.23
12609	*Cebpd*	CCAAT/enhancer binding protein (C/EBP), delta	0.08	-0.27	-0.30	-0.24
110794	*Cebpe*	CCAAT/enhancer binding protein (C/EBP), epsilon	-0.18	0.39	-2.24	0.18
26371	*Ciao1*	cytosolic iron-sulfur protein assembly 1 homolog (S. cerevisiae)	0.04	0.07	-0.05	0.21
12753	*Clock*	circadian locomotor output cycles kaput	0.13	0.19	0.44	0.16
12912	*Creb1*	cAMP responsive element binding protein 1	-0.25	-0.10	-0.34	-0.29

geneid	symbol	name	Day 5	Day 8	Day 11	Day 14
12913	*Creb3*	cAMP responsive element binding protein 3	0.40	0.23	2.15	0.30
26427	*Creb3l1*	cAMP responsive element binding protein 3-like 1	0.05	-0.05	1.58	-0.05
208647	*Creb3l2*	cAMP responsive element binding protein 3-like 2	0.30	0.40	2.38	0.89
208677	*Creb3l3*	cAMP responsive element binding protein 3-like 3	0.09	0.09	1.07	0.06
78284	*Creb3l4*	cAMP responsive element binding protein 3-like 4	-0.20	0.36	0.68	0.48
231991	*Creb5*	cAMP responsive element binding protein 5	-0.09	0.26	1.90	0.12
232430	*Crebl2*	cAMP responsive element binding protein-like 2	0.35	0.25	1.07	0.35
233490	*Crebzf*	CREB/ATF bZIP transcription factor	-0.33	-0.18	-0.30	-0.20
12916	*Crem*	cAMP responsive element modulator	0.19	0.24	0.27	-0.12
12951	*Crx*	cone-rod homeobox containing gene	-0.20	-0.05	-0.07	-0.10
215418	*Csrnp1*	cysteine-serine-rich nuclear protein 1	0.02	0.35	0.67	0.21
207785	*Csrnp2*	cysteine-serine-rich nuclear protein 2	-0.02	0.50	-0.39	0.30
77771	*Csrnp3*	cysteine-serine-rich nuclear protein 3	-0.27	-0.09	0.19	-0.47
13016	*Ctbp1*	C-terminal binding protein 1	0.00	0.01	-0.19	-0.02
13018	*Ctcf*	CCCTC-binding factor	-0.07	0.08	-0.54	-0.09
13047	*Cux1*	cut-like homeobox 1	0.08	0.24	-0.29	0.24
13048	*Cux2*	cut-like homeobox 2	0.58	0.34	0.91	0.59
13134	*Dach1*	dachshund 1 (Drosophila)	-1.05	0.40	-0.30	1.20
13170	*Dbp*	D site albumin promoter binding protein	0.27	0.39	-0.43	0.61
13172	*Dbx1*	developing brain homeobox 1	0.41	1.11	-0.15	0.50
13198	*Ddit3*	DNA-damage inducible transcript 3	0.89	0.56	1.00	0.16
13390	*Dlx1*	distal-less homeobox 1	-0.38	0.88	0.28	0.91
13392	*Dlx2*	distal-less homeobox 2	1.16	0.21	0.37	0.63
13394	*Dlx4*	distal-less homeobox 4	-0.45	0.99	1.40	1.34
13395	*Dlx5*	distal-less homeobox 5	-0.21	0.18	-0.16	0.59
13396	*Dlx6*	distal-less homeobox 6	-0.79	1.06	-2.81	1.31
140477	*Dmbx1*	diencephalon/mesencephalon homeobox 1	0.54	0.30	0.75	0.82
50796	*Dmrt1*	doublesex and mab-3 related transcription factor 1	1.22	0.76	-1.50	0.54
240590	*Dmrt3*	doublesex and mab-3 related transcription factor 3	1.05	0.71	-0.39	2.21
242523	*Dmrta1*	doublesex and mab-3 related transcription factor like family A1	-0.07	-0.41	-0.53	-0.88
242620	*Dmrta2*	doublesex and mab-3 related transcription factor like family A2	0.88	-0.14	1.80	0.43
56296	*Dmrtb1*	DMRT-like family B with proline-rich C-terminal, 1	0.90	0.14	-2.15	0.23
71241	*Dmrtc2*	doublesex and mab-3 related transcription factor like family C2	0.68	0.68	0.24	0.26
13555	*E2f1*	E2F transcription factor 1	0.33	0.00	-0.33	-0.09
242705	*E2f2*	E2F transcription factor 2	-0.49	-0.04	0.17	-0.19
13557	*E2f3*	E2F transcription factor 3	-0.37	-0.15	-0.29	-0.26
104394	*E2f4*	E2F transcription factor 4	-0.28	-0.13	-0.53	-0.27
13559	*E2f5*	E2F transcription factor 5	0.17	0.04	-0.22	0.22

geneid	symbol	name	Day 5	Day 8	Day 11	Day 14
50496	*E2f6*	E2F transcription factor 6	0.02	0.12	-0.29	-0.01
52679	*E2f7*	E2F transcription factor 7	-0.15	-0.02	-0.30	-0.19
108961	*E2f8*	E2F transcription factor 8	-0.03	0.40	0.20	0.02
13591	*Ebf1*	early B-cell factor 1	-1.31	-0.75	-0.34	-0.45
26940	*Ecsit*	ECSIT homolog (Drosophila)	-0.18	-0.23	-0.50	-0.53
13653	*Egr1*	early growth response 1	-0.72	-0.34	-0.38	-0.61
13654	*Egr2*	early growth response 2	-0.64	0.18	-0.23	-0.76
13661	*Ehf*	ets homologous factor	0.09	-0.28	0.23	0.50
13709	*Elf1*	E74-like factor 1	0.32	0.49	1.10	0.70
69257	*Elf2*	E74-like factor 2	0.33	0.43	0.42	0.47
13710	*Elf3*	E74-like factor 3	0.88	0.54	0.47	0.87
56501	*Elf4*	E74-like factor 4 (ets domain transcription factor)	0.68	0.66	2.20	1.19
13711	*Elf5*	E74-like factor 5	1.08	1.35	1.06	2.31
13712	*Elk1*	ELK1, member of ETS oncogene family	0.11	0.09	0.29	0.03
13713	*Elk3*	ELK3, member of ETS oncogene family	0.01	0.66	1.59	0.69
13714	*Elk4*	ELK4, member of ETS oncogene family	-0.28	0.27	0.22	-0.06
13796	*Emx1*	empty spiracles homolog 1 (Drosophila)	0.85	0.94	1.21	2.95
13797	*Emx2*	empty spiracles homolog 2 (Drosophila)	-0.69	0.28	0.69	0.79
13798	*En1*	engrailed 1	-0.22	0.35	1.01	-0.03
13799	*En2*	engrailed 2	0.27	0.54	-0.70	0.60
13813	*Eomes*	eomesodermin homolog (Xenopus laevis)	-0.48	0.53	-0.31	1.07
328572	*Ep300*	E1A binding protein p300	-0.05	0.15	0.09	0.01
13875	*Erf*	Ets2 repressor factor	0.52	0.20	0.24	0.23
13876	*Erg*	avian erythroblastosis virus E-26 (v-ets) oncogene related	-0.01	0.05	-0.34	0.65
13982	*Esr1*	estrogen receptor 1 (alpha)	-0.41	-0.13	-0.78	1.23
13983	*Esr2*	estrogen receptor 2 (beta)	-0.62	-0.35	-0.67	-0.76
26379	*Esrra*	estrogen related receptor, alpha	0.44	0.11	0.37	0.17
26380	*Esrrb*	estrogen related receptor, beta	1.36	0.34	1.32	0.41
26381	*Esrrg*	estrogen-related receptor gamma	-0.22	-0.46	-0.85	-0.54
23872	*Ets2*	E26 avian leukemia oncogene 2, 3' domain	0.33	1.04	1.06	1.34
14009	*Etv1*	ets variant gene 1	-1.81	-0.78	-1.17	-1.47
14008	*Etv2*	ets variant gene 2	-1.69	-1.41	-2.45	-1.69
27049	*Etv3*	ets variant gene 3	0.22	0.40	0.89	0.53
18612	*Etv4*	ets variant gene 4 (E1A enhancer binding protein, E1AF)	-0.57	-0.49	-0.13	-1.03
104156	*Etv5*	ets variant gene 5	-0.43	-0.20	-0.65	-0.45
14011	*Etv6*	ets variant gene 6 (TEL oncogene)	-0.61	0.01	-0.15	-0.03
14028	*Evx1*	even skipped homeotic gene 1 homolog	-1.84	-0.30	-0.22	-0.53
14029	*Evx2*	even skipped homeotic gene 2 homolog	0.81	0.29	-0.14	0.46
50754	*Fbxw7*	F-box and WD-40 domain protein 7	0.04	0.00	0.13	-0.06

geneid	symbol	name	Day 5	Day 8	Day 11	Day 14
260298	*Fev*	FEV (ETS oncogene family)	0.31	0.29	-0.64	-0.68
14247	*Fli1*	Friend leukemia integration 1	-0.25	1.27	-0.50	-0.46
14281	*Fos*	FBJ osteosarcoma oncogene	0.61	0.45	1.32	1.06
14282	*Fosb*	FBJ osteosarcoma oncogene B	-0.01	0.69	0.64	0.93
14283	*Fosl1*	fos-like antigen 1	-0.23	0.26	0.30	-0.64
14284	*Fosl2*	fos-like antigen 2	0.59	-0.19	3.63	-0.08
15375	*Foxa1*	forkhead box A1	0.03	-0.75	0.32	0.15
15377	*Foxa3*	forkhead box A3	-0.30	0.19	-0.57	-0.20
64290	*Foxb1*	forkhead box B1	-0.71	0.07	0.03	-0.73
14240	*Foxb2*	forkhead box B2	-0.13	1.18	1.45	1.20
15229	*Foxd1*	forkhead box D1	0.26	-0.27	-0.03	0.60
17301	*Foxd2*	forkhead box D2	1.13	-0.40	0.80	1.39
15221	*Foxd3*	forkhead box D3	-1.32	-1.10	-1.40	-0.99
14237	*Foxd4*	forkhead box D4	-1.51	0.12	-0.34	-1.31
110805	*Foxe1*	forkhead box E1	0.03	-2.16	0.72	0.58
30923	*Foxe3*	forkhead box E3	-0.13	0.85	-0.14	-0.11
15227	*Foxf1a*	forkhead box F1a	0.39	0.69	0.43	0.75
14238	*Foxf2*	forkhead box F2	0.86	0.02	1.00	-0.57
14233	*Foxi1*	forkhead box I1	0.41	0.44	-0.14	-0.11
270004	*Foxi2*	forkhead box I2	0.03	-0.53	-0.15	1.62
15223	*Foxj1*	forkhead box J1	0.13	-0.12	-0.18	0.17
60611	*Foxj2*	forkhead box J2	0.35	0.73	1.26	0.78
230700	*Foxj3*	forkhead box J3	0.30	0.28	0.44	0.21
17425	*Foxk1*	forkhead box K1	0.11	0.26	0.12	0.02
68837	*Foxk2*	forkhead box K2	-0.07	-0.05	-0.29	-0.22
14241	*Foxl1*	forkhead box L1	-0.01	-0.02	-0.16	-0.63
26927	*Foxl2*	forkhead box L2	0.62	-0.28	1.62	-0.31
14235	*Foxm1*	forkhead box M1	-0.04	-0.14	-0.39	-0.03
15218	*Foxn1*	forkhead box N1	0.39	-0.15	1.39	-1.82
71375	*Foxn3*	forkhead box N3	-0.14	0.29	-0.09	-0.10
116810	*Foxn4*	forkhead box N4	0.07	0.97	-1.41	1.47
56458	*Foxo1*	forkhead box O1	-0.04	0.03	-0.07	0.07
56484	*Foxo3*	forkhead box O3	0.44	0.45	0.11	0.38
54601	*Foxo4*	forkhead box O4	-0.36	0.78	-0.44	0.91
329934	*Foxo6*	forkhead box O6	0.56	0.77	-0.57	1.17
114142	*Foxp2*	forkhead box P2	-0.25	-0.25	0.38	-0.39
20371	*Foxp3*	forkhead box P3	-0.42	-0.15	-0.15	-0.15
74123	*Foxp4*	forkhead box P4	0.00	-0.03	0.00	0.22
15220	*Foxq1*	forkhead box Q1	0.75	0.19	3.05	0.68
382074	*Foxr1*	forkhead box R1	0.03	-0.22	-0.04	-0.51

geneid	symbol	name	Day 5	Day 8	Day 11	Day 14
436240	*Foxr2*	forkhead box R2	-1.63	-1.00	-1.55	-1.42
14239	*Foxs1*	forkhead box S1	0.90	-1.75	-0.14	0.50
14390	*Gabpa*	GA repeat binding protein, alpha	-0.08	-0.02	-0.14	-0.20
14460	*Gata1*	GATA binding protein 1	0.97	1.41	-0.32	-1.27
14464	*Gata5*	GATA binding protein 5	-0.38	1.01	1.10	0.43
67210	*Gatad1*	GATA zinc finger domain containing 1	-0.05	-0.19	-0.09	-0.09
229542	*Gatad2b*	GATA zinc finger domain containing 2B	0.11	0.25	0.04	-0.04
231044	*Gbx1*	gastrulation brain homeobox 1	0.29	-1.46	-0.31	-0.59
67367	*Gcfc1*	GC-rich sequence DNA-binding factor 1	-0.16	-0.14	-0.02	-0.14
14531	*Gcm1*	glial cells missing homolog 1 (Drosophila)	0.21	0.12	-0.72	-1.64
107889	*Gcm2*	glial cells missing homolog 2 (Drosophila)	0.41	0.86	-0.14	0.68
14633	*Gli2*	GLI-Kruppel family member GLI2	0.06	-0.35	-0.05	-0.35
83396	*Glis2*	GLIS family zinc finger 2	-0.12	0.13	0.82	0.46
226075	*Glis3*	GLIS family zinc finger 3	0.01	0.42	0.93	0.51
225908	*Gm98*	predicted gene 98	-1.48	-1.35	-1.43	-1.99
56809	*Gmeb1*	glucocorticoid modulatory element binding protein 1	-0.40	-0.24	-0.65	-0.44
73274	*Gpbp1*	GC-rich promoter binding protein 1	0.23	0.26	0.06	0.26
195733	*Grhl1*	grainyhead-like 1 (Drosophila)	0.40	-0.03	-0.18	0.31
252973	*Grhl2*	grainyhead-like 2 (Drosophila)	-0.18	-0.49	-0.57	-0.33
14836	*Gsc*	goosecoid homeobox	0.46	0.87	-0.31	1.99
195333	*Gsc2*	goosecoid homebox 2	0.51	0.24	-1.02	1.18
14842	*Gsx1*	GS homeobox 1	-0.74	0.04	-2.10	-1.56
14843	*Gsx2*	GS homeobox 2	2.28	0.13	-0.08	1.46
57080	*Gtf2ird1*	general transcription factor II I repeat domain-containing 1	0.13	-0.02	-0.06	0.18
66596	*Gtf3a*	general transcription factor III A	0.05	0.03	-0.26	-0.01
15182	*Hdac2*	histone deacetylase 2	-0.06	-0.07	-0.09	-0.07
245596	*Hdx*	highly divergent homeobox	-0.75	-0.12	-0.57	-0.77
234219	*Helt*	Hey-like transcription factor (zebrafish)	0.14	0.44	-0.66	-1.08
15205	*Hes1*	hairy and enhancer of split 1 (Drosophila)	-0.24	-0.03	0.44	0.41
55927	*Hes6*	hairy and enhancer of split 6 (Drosophila)	-0.93	-0.56	-1.58	-0.64
15209	*Hesx1*	homeobox gene expressed in ES cells	1.06	-0.01	0.15	0.33
15213	*Hey1*	hairy/enhancer-of-split related with YRPW motif 1	-0.29	0.02	0.03	0.85
56198	*Heyl*	hairy/enhancer-of-split related with YRPW motif-like	-0.37	0.86	0.04	1.23
15242	*Hhex*	hematopoietically expressed homeobox	0.26	0.28	0.05	0.30
15251	*Hif1a*	hypoxia inducible factor 1, alpha subunit	-0.05	0.18	-0.15	-0.01
53417	*Hif3a*	hypoxia inducible factor 3, alpha subunit	0.02	-0.02	-0.63	0.43
102423	*Hinfp*	histone H4 transcription factor	0.07	0.14	0.39	-0.01
15273	*Hivep2*	human immunodeficiency virus type I enhancer binding protein 2	-0.64	0.19	1.11	0.25

geneid	symbol	name	Day 5	Day 8	Day 11	Day 14
217082	*Hlf*	hepatic leukemia factor	-0.74	-0.80	-1.35	-2.22
15284	*Hlx*	H2.0-like homeobox	-0.56	1.14	0.13	0.25
219150	*Hmbox1*	homeobox containing 1	-0.13	0.31	0.38	0.06
15353	*Hmg20b*	high mobility group 20 B	0.24	0.07	0.70	0.40
15361	*Hmga1*	high mobility group AT-hook 1	-0.26	-0.43	-1.18	-0.74
15289	*Hmgb1*	high mobility group box 1	-0.15	-0.11	-0.84	-0.33
97165	*Hmgb2*	high mobility group box 2	0.03	-0.01	-0.83	-0.25
15371	*Hmx1*	H6 homeobox 1	0.41	0.99	-0.04	1.60
15372	*Hmx2*	H6 homeobox 2	0.27	0.38	-0.14	0.23
15373	*Hmx3*	H6 homeobox 3	0.91	0.08	-0.14	-0.11
21410	*Hnf1b*	HNF1 homeobox B	0.56	0.47	0.63	1.49
15378	*Hnf4a*	hepatic nuclear factor 4, alpha	0.41	0.88	0.85	1.96
30942	*Hnf4g*	hepatocyte nuclear factor 4, gamma	0.11	-0.13	0.10	-0.11
15384	*Hnrnpab*	heterogeneous nuclear ribonucleoprotein A/B	-0.14	-0.15	-0.57	-0.27
239099	*Homez*	homeodomain leucine zipper-encoding gene	-0.49	0.07	-0.41	0.06
15396	*Hoxa11*	homeobox A11	0.48	0.68	0.03	-0.26
15398	*Hoxa13*	homeobox A13	0.81	0.61	0.86	1.32
15399	*Hoxa2*	homeobox A2	0.64	0.07	0.23	0.36
15401	*Hoxa4*	homeobox A4	0.08	1.53	-1.09	1.41
15402	*Hoxa5*	homeobox A5	-0.13	1.56	0.33	0.45
15403	*Hoxa6*	homeobox A6	0.81	0.52	0.86	1.48
15404	*Hoxa7*	homeobox A7	0.46	0.51	0.31	0.16
15405	*Hoxa9*	homeobox A9	-0.70	1.05	-0.27	0.09
15407	*Hoxb1*	homeobox B1	-0.89	0.93	-2.89	-0.53
15408	*Hoxb13*	homeobox B13	-0.46	0.87	-0.16	0.79
103889	*Hoxb2*	homeobox B2	-0.93	0.68	-2.85	1.54
15412	*Hoxb4*	homeobox B4	0.78	0.31	0.93	0.78
15414	*Hoxb6*	homeobox B6	1.48	0.44	-1.57	-1.08
15415	*Hoxb7*	homeobox B7	0.18	0.99	-0.16	-0.38
15416	*Hoxb8*	homeobox B8	-0.12	0.05	-0.56	-1.09
15417	*Hoxb9*	homeobox B9	0.61	-0.99	1.49	-1.98
209448	*Hoxc10*	homeobox C10	0.60	1.29	-1.05	1.21
109663	*Hoxc11*	homeobox C11	0.81	-0.17	-0.14	-0.11
15421	*Hoxc12*	homeobox C12	-0.59	-0.12	-0.50	-0.07
15422	*Hoxc13*	homeobox C13	0.61	-0.52	0.41	0.02
15423	*Hoxc4*	homeobox C4	0.15	0.93	0.69	2.02
15424	*Hoxc5*	homeobox C5	-0.22	0.93	-0.22	-0.27
15425	*Hoxc6*	homeobox C6	1.10	0.19	0.37	0.54
15426	*Hoxc8*	homeobox C8	-0.26	0.26	-0.17	-0.30
15427	*Hoxc9*	homeobox C9	1.41	0.20	-0.42	0.62

**geneid**	**symbol**	**name**	**Day 5**	**Day 8**	**Day 11**	**Day 14**
15429	*Hoxd1*	homeobox D1	-1.32	0.79	-0.85	1.07
15431	*Hoxd11*	homeobox D11	0.50	0.65	-1.41	0.20
15432	*Hoxd12*	homeobox D12	1.08	0.08	-2.14	-0.68
15433	*Hoxd13*	homeobox D13	-0.57	0.60	-2.50	-0.07
15434	*Hoxd3*	homeobox D3	-1.01	0.59	-0.92	0.18
15436	*Hoxd4*	homeobox D4	1.06	1.51	-0.94	1.72
15437	*Hoxd8*	homeobox D8	1.28	0.02	2.43	1.04
15438	*Hoxd9*	homeobox D9	0.54	1.74	1.54	0.95
15499	*Hsf1*	heat shock factor 1	-0.09	0.14	-0.06	0.12
15500	*Hsf2*	heat shock factor 2	0.23	0.03	-0.07	-0.04
245525	*Hsf3*	heat shock transcription factor 3	-0.13	0.44	-0.52	-0.54
26386	*Hsf4*	heat shock transcription factor 4	0.56	0.31	-0.08	0.82
327992	*Hsf5*	heat shock transcription factor family member 5	0.31	-0.08	-0.57	0.23
15901	*Id1*	inhibitor of DNA binding 1	-0.19	-0.21	0.51	0.66
22778	*Ikzf1*	IKAROS family zinc finger 1	-0.76	0.72	-0.62	0.44
16362	*Irf1*	interferon regulatory factor 1	-0.73	0.06	-1.40	-0.04
16363	*Irf2*	interferon regulatory factor 2	0.20	1.01	2.15	1.15
54131	*Irf3*	interferon regulatory factor 3	-0.07	0.09	0.16	0.27
16364	*Irf4*	interferon regulatory factor 4	-0.37	1.54	-1.68	-0.42
27056	*Irf5*	interferon regulatory factor 5	0.16	-0.10	0.99	0.54
54139	*Irf6*	interferon regulatory factor 6	0.58	-0.18	1.45	0.37
54123	*Irf7*	interferon regulatory factor 7	0.08	1.87	0.04	2.72
15900	*Irf8*	interferon regulatory factor 8	-0.70	0.56	-1.22	0.06
16391	*Irf9*	interferon regulatory factor 9	-0.01	0.46	0.54	0.59
16371	*Irx1*	Iroquois related homeobox 1 (Drosophila)	0.23	0.31	0.02	0.92
16372	*Irx2*	Iroquois related homeobox 2 (Drosophila)	0.51	0.87	0.56	1.23
16373	*Irx3*	Iroquois related homeobox 3 (Drosophila)	0.06	1.03	-0.62	2.50
54352	*Irx5*	Iroquois related homeobox 5 (Drosophila)	-0.35	-0.04	-0.92	0.54
64379	*Irx6*	Iroquois related homeobox 6 (Drosophila)	-0.25	0.63	-2.25	2.74
104360	*Isl2*	insulin related protein 2 (islet 2)	0.73	-0.06	0.21	0.01
71597	*Isx*	intestine specific homeobox	-0.59	-1.16	0.26	-0.11
81703	*Jdp2*	Jun dimerization protein 2	0.93	0.69	1.95	0.93
16477	*Junb*	Jun-B oncogene	-0.13	0.61	1.90	0.90
16478	*Jund*	Jun proto-oncogene related gene d	0.47	0.36	0.62	0.79
99982	*Kdm1a*	lysine (K)-specific demethylase 1A	0.12	0.20	-0.15	0.25
194655	*Klf11*	Kruppel-like factor 11	1.02	0.35	0.34	0.44
16597	*Klf12*	Kruppel-like factor 12	-0.65	-0.35	0.36	-0.91
66277	*Klf15*	Kruppel-like factor 15	-0.08	0.12	0.42	0.46
118445	*Klf16*	Kruppel-like factor 16	0.09	-0.05	-0.43	-0.04
75753	*Klf17*	Kruppel-like factor 17	0.08	-0.38	-0.52	-0.82

geneid	symbol	name	Day 5	Day 8	Day 11	Day 14
16600	*Klf4*	Kruppel-like factor 4 (gut)	1.41	0.27	1.62	0.37
23849	*Klf6*	Kruppel-like factor 6	0.25	0.59	1.64	0.65
93691	*Klf7*	Kruppel-like factor 7 (ubiquitous)	-0.30	0.11	0.84	-0.43
320858	*L3mbtl4*	l(3)mbt-like 4 (Drosophila)	0.27	-0.87	-0.14	-0.11
76893	*Lass2*	LAG1 homolog, ceramide synthase 2	-0.40	0.22	0.40	0.38
67260	*Lass4*	LAG1 homolog, ceramide synthase 4	-0.46	-0.93	-0.15	-0.78
71949	*Lass5*	LAG1 homolog, ceramide synthase 5	-0.25	0.01	0.49	0.00
241447	*Lass6*	LAG1 homolog, ceramide synthase 6	-0.43	-0.07	0.94	-0.30
16814	*Lbx1*	ladybird homeobox homolog 1 (Drosophila)	0.75	0.87	0.59	-0.09
16815	*Lbx2*	ladybird homeobox homolog 2 (Drosophila)	1.02	0.43	0.44	-0.44
212391	*Lcor*	ligand dependent nuclear receptor corepressor	0.22	0.43	0.69	0.51
16869	*Lhx1*	LIM homeobox protein 1	-2.22	-0.20	0.48	0.72
16870	*Lhx2*	LIM homeobox protein 2	1.35	1.97	-0.33	1.35
16871	*Lhx3*	LIM homeobox protein 3	-1.15	-0.39	-3.28	-0.45
16872	*Lhx4*	LIM homeobox protein 4	-0.24	-0.84	-0.03	-0.32
16873	*Lhx5*	LIM homeobox protein 5	-0.27	-0.57	0.35	-0.67
16874	*Lhx6*	LIM homeobox protein 6	-0.08	0.33	0.38	-0.01
16875	*Lhx8*	LIM homeobox protein 8	-0.24	0.21	-1.66	-0.11
16876	*Lhx9*	LIM homeobox protein 9	1.39	1.33	-1.41	-0.10
110648	*Lmx1a*	LIM homeobox transcription factor 1 alpha	-0.10	0.19	-1.00	0.28
16917	*Lmx1b*	LIM homeobox transcription factor 1 beta	1.33	0.44	-0.59	1.27
17132	*Maf*	avian musculoaponeurotic fibrosarcoma (v-maf) AS42 oncogene homolog	0.30	0.45	1.08	0.37
378435	*Mafa*	v-maf musculoaponeurotic fibrosarcoma oncogene family, protein A (avian)	0.93	0.54	-0.25	0.81
16658	*Mafb*	v-maf musculoaponeurotic fibrosarcoma oncogene family, protein B (avian)	0.34	0.37	-0.98	-0.48
17133	*Maff*	v-maf musculoaponeurotic fibrosarcoma oncogene family, protein F (avian)	-0.19	0.35	0.70	-0.31
17134	*Mafg*	v-maf musculoaponeurotic fibrosarcoma oncogene family, protein G (avian)	0.06	0.01	-0.23	-0.06
17135	*Mafk*	v-maf musculoaponeurotic fibrosarcoma oncogene family, protein K (avian)	0.13	0.46	0.87	0.22
17187	*Max*	Max protein	-0.05	0.07	-0.14	-0.06
14013	*Mecom*	MDS1 and EVI1 complex locus	-0.65	-0.68	0.09	0.16
17257	*Mecp2*	methyl CpG binding protein 2	0.04	0.03	0.34	0.22
17258	*Mef2a*	myocyte enhancer factor 2A	0.09	0.07	0.87	0.07
17259	*Mef2b*	myocyte enhancer factor 2B	0.69	0.14	-0.08	-0.26
17261	*Mef2d*	myocyte enhancer factor 2D	0.16	0.60	1.09	0.66
17536	*Meis2*	Meis homeobox 2	-0.51	0.46	1.59	0.98
17537	*Meis3*	Meis homeobox 3	-0.16	0.11	1.85	0.73
17285	*Meox1*	mesenchyme homeobox 1	-0.05	0.52	0.80	0.14
17286	*Meox2*	mesenchyme homeobox 2	-0.59	0.08	-0.92	-0.07

geneid	symbol	name	Day 5	Day 8	Day 11	Day 14
29808	*Mga*	MAX gene associated	0.02	-0.04	-0.04	-0.20
17342	*Mitf*	microphthalmia-associated transcription factor	0.35	0.38	1.00	0.80
27217	*Mixl1*	Mix1 homeobox-like 1 (Xenopus laevis)	-2.07	0.00	-1.43	0.19
210719	*Mkx*	mohawk homeobox	-0.37	0.20	-0.48	0.18
17428	*Mnt*	max binding protein	0.16	0.40	-0.01	0.16
15285	*Mnx1*	motor neuron and pancreas homeobox 1	0.23	-0.62	-0.64	1.23
67871	*Mrrf*	mitochondrial ribosome recycling factor	0.28	0.05	-0.31	-0.35
17702	*Msx2*	homeobox, msh-like 2	0.46	-0.16	2.86	0.47
17703	*Msx3*	homeobox, msh-like 3	-0.62	-0.12	-1.41	-1.32
116870	*Mta1*	metastasis associated 1	-0.22	0.02	-0.48	-0.01
23942	*Mta2*	metastasis-associated gene family, member 2	-0.07	0.03	-0.07	-0.03
116871	*Mta3*	metastasis associated 3	0.40	0.29	-0.29	0.32
17764	*Mtf1*	metal response element binding transcription factor 1	0.10	0.39	0.30	0.30
17863	*Myb*	myeloblastosis oncogene	-0.84	-1.03	-1.53	-1.31
16918	*Mycl1*	v-myc myelocytomatosis viral oncogene homolog 1, lung carcinoma derived (avian)	-0.36	-0.38	-0.90	-0.07
17870	*Mycs*	myc-like oncogene, s-myc protein	0.81	0.44	-0.14	-0.07
17927	*Myod1*	myogenic differentiation 1	-1.22	-0.75	-2.53	-1.11
17928	*Myog*	myogenin	0.59	1.53	-0.54	1.19
232934	*Mypop*	Myb-related transcription factor, partner of profilin	-0.30	0.36	-0.62	0.43
217127	*Myst2*	MYST histone acetyltransferase 2	0.40	0.36	-0.05	0.23
17932	*Myt1*	myelin transcription factor 1	-0.08	0.21	0.57	1.40
17933	*Myt1l*	myelin transcription factor 1-like	-1.49	0.49	-1.41	-0.35
20185	*Ncor1*	nuclear receptor co-repressor 1	-0.12	0.10	-0.18	0.05
18012	*Neurod1*	neurogenic differentiation 1	-0.39	-1.40	-2.37	-2.14
11925	*Neurog3*	neurogenin 3	0.79	-1.56	-2.91	-2.83
54446	*Nfat5*	nuclear factor of activated T-cells 5	-0.27	-0.02	0.64	0.04
18018	*Nfatc1*	nuclear factor of activated T-cells, cytoplasmic, calcineurin-dependent 1	0.45	0.41	0.62	0.60
18019	*Nfatc2*	nuclear factor of activated T-cells, cytoplasmic, calcineurin-dependent 2	-0.05	0.28	1.20	0.48
18021	*Nfatc3*	nuclear factor of activated T-cells, cytoplasmic, calcineurin-dependent 3	0.01	0.26	-0.11	0.08
73181	*Nfatc4*	nuclear factor of activated T-cells, cytoplasmic, calcineurin-dependent 4	-0.02	0.05	0.35	0.46
18022	*Nfe2*	nuclear factor, erythroid derived 2	-0.31	0.55	0.47	-0.32
18023	*Nfe2l1*	nuclear factor, erythroid derived 2,-like 1	-0.25	0.07	0.26	0.04
18024	*Nfe2l2*	nuclear factor, erythroid derived 2, like 2	0.24	0.12	-0.24	0.16
18025	*Nfe2l3*	nuclear factor, erythroid derived 2, like 3	-0.59	0.19	-0.28	0.59
18027	*Nfia*	nuclear factor I/A	0.53	0.32	0.05	0.47
18028	*Nfib*	nuclear factor I/B	0.05	-0.31	0.13	-0.27
18029	*Nfic*	nuclear factor I/C	0.26	0.11	0.19	0.18

geneid	symbol	name	Day 5	Day 8	Day 11	Day 14
18030	*Nfil3*	nuclear factor, interleukin 3, regulated	0.13	0.23	0.50	0.19
18032	*Nfix*	nuclear factor I/X	0.73	0.74	0.24	0.43
18033	*Nfkb1*	nuclear factor of kappa light polypeptide gene enhancer in B-cells 1, p105	0.16	0.14	0.70	0.27
18034	*Nfkb2*	nuclear factor of kappa light polypeptide gene enhancer in B-cells 2, p49/p100	-0.03	-0.05	1.34	-0.05
74164	*Nfx1*	nuclear transcription factor, X-box binding 1	-0.13	0.01	-0.43	-0.14
18044	*Nfya*	nuclear transcription factor-Y alpha	0.06	0.33	-0.08	0.20
18045	*Nfyb*	nuclear transcription factor-Y beta	0.19	-0.18	-0.48	-0.23
18046	*Nfyc*	nuclear transcription factor-Y gamma	0.06	0.00	-0.59	-0.16
20231	*Nkx1-2*	NK1 transcription factor related, locus 2 (Drosophila)	-3.89	0.05	-1.63	-0.40
21869	*Nkx2-1*	NK2 homeobox 1	-0.29	-0.62	0.66	-0.56
18088	*Nkx2-2*	NK2 transcription factor related, locus 2 (Drosophila)	-0.14	0.18	0.57	-0.50
18089	*Nkx2-3*	NK2 transcription factor related, locus 3 (Drosophila)	-1.00	0.32	-0.43	1.67
228731	*Nkx2-4*	NK2 transcription factor related, locus 4 (Drosophila)	-0.09	0.55	-1.61	0.81
18094	*Nkx2-9*	NK2 transcription factor related, locus 9 (Drosophila)	0.67	-0.50	1.11	-1.01
18095	*Nkx3-1*	NK-3 transcription factor, locus 1 (Drosophila)	-0.18	0.81	-0.50	0.54
12020	*Nkx3-2*	NK3 homeobox 2	1.39	0.44	-0.25	-0.71
18096	*Nkx6-1*	NK6 homeobox 1	0.80	0.75	-0.05	0.54
14912	*Nkx6-2*	NK6 homeobox 2	0.28	-0.21	0.30	-0.04
74561	*Nkx6-3*	NK6 homeobox 3	0.16	0.99	1.18	0.07
18291	*Nobox*	NOBOX oogenesis homeobox	-0.20	-0.23	-0.06	-0.03
70769	*Nolc1*	nucleolar and coiled-body phosphoprotein 1	-0.20	-0.32	-0.85	-0.55
18128	*Notch1*	Notch gene homolog 1 (Drosophila)	0.01	-0.65	0.21	-0.19
384452	*Noto*	notochord homolog (Xenopus laevis)	-0.53	-0.41	-0.14	0.50
18143	*Npas2*	neuronal PAS domain protein 2	0.04	-0.08	0.49	0.68
244879	*Npat*	nuclear protein in the AT region	0.13	0.14	0.21	0.15
11614	*Nr0b1*	nuclear receptor subfamily 0, group B, member 1	1.13	0.04	0.83	0.08
23957	*Nr0b2*	nuclear receptor subfamily 0, group B, member 2	0.16	1.18	-0.62	0.12
217166	*Nr1d1*	nuclear receptor subfamily 1, group D, member 1	0.28	0.18	-0.35	0.20
353187	*Nr1d2*	nuclear receptor subfamily 1, group D, member 2	0.28	0.06	-0.16	-0.21
22260	*Nr1h2*	nuclear receptor subfamily 1, group H, member 2	-0.02	0.08	-0.11	0.06
22259	*Nr1h3*	nuclear receptor subfamily 1, group H, member 3	-0.41	-0.15	0.23	-0.05
20186	*Nr1h4*	nuclear receptor subfamily 1, group H, member 4	-0.22	-0.13	0.50	-0.11
18171	*Nr1i2*	nuclear receptor subfamily 1, group I, member 2	-0.53	2.19	0.50	2.65
12355	*Nr1i3*	nuclear receptor subfamily 1, group I, member 3	-0.53	-0.87	-0.51	-0.59
22025	*Nr2c1*	nuclear receptor subfamily 2, group C, member 1	-0.35	-0.22	0.23	0.18
22026	*Nr2c2*	nuclear receptor subfamily 2, group C, member 2	-0.10	0.02	-0.03	-0.13
21907	*Nr2e1*	nuclear receptor subfamily 2, group E, member 1	0.18	0.83	-0.54	-1.44

geneid	symbol	name	Day 5	Day 8	Day 11	Day 14
23958	*Nr2e3*	nuclear receptor subfamily 2, group E, member 3	0.41	0.98	0.57	-0.11
13865	*Nr2f1*	nuclear receptor subfamily 2, group F, member 1	-0.20	0.20	1.39	0.99
13864	*Nr2f6*	nuclear receptor subfamily 2, group F, member 6	-0.19	-0.07	-0.21	0.24
14815	*Nr3c1*	nuclear receptor subfamily 3, group C, member 1	0.12	0.18	0.00	0.28
110784	*Nr3c2*	nuclear receptor subfamily 3, group C, member 2	0.59	1.33	0.72	0.90
18227	*Nr4a2*	nuclear receptor subfamily 4, group A, member 2	-0.09	0.38	0.55	0.46
18124	*Nr4a3*	nuclear receptor subfamily 4, group A, member 3	0.76	1.22	1.35	1.63
14536	*Nr6a1*	nuclear receptor subfamily 6, group A, member 1	-0.01	0.23	-0.36	0.09
18181	*Nrf1*	nuclear respiratory factor 1	-0.06	0.07	-0.26	-0.10
18185	*Nrl*	neural retina leucine zipper gene	0.81	0.66	-0.03	-0.44
50914	*Olig1*	oligodendrocyte transcription factor 1	-0.58	0.08	0.41	0.29
50913	*Olig2*	oligodendrocyte transcription factor 2	-0.89	-0.52	-0.62	-0.25
15379	*Onecut1*	one cut domain, family member 1	0.22	0.92	-1.22	1.67
225631	*Onecut2*	one cut domain, family member 2	-0.37	0.10	0.78	0.52
246086	*Onecut3*	one cut domain, family member 3	0.34	1.06	-0.29	-0.02
18420	*Otp*	orthopedia homolog (Drosophila)	0.27	0.29	-0.03	-0.68
18423	*Otx1*	orthodenticle homolog 1 (Drosophila)	-0.01	0.93	-0.86	0.72
18424	*Otx2*	orthodenticle homolog 2 (Drosophila)	-1.66	-1.32	-2.17	-1.83
18426	*Ovol1*	OVO homolog-like 1 (Drosophila)	0.13	0.37	-0.67	0.69
107586	*Ovol2*	ovo-like 2 (Drosophila)	-0.04	-0.09	-0.96	0.34
18813	*Pa2g4*	proliferation-associated 2G4	-0.18	-0.24	-0.73	-0.43
18503	*Pax1*	paired box gene 1	0.02	-0.44	-1.47	-1.20
18504	*Pax2*	paired box gene 2	0.20	0.82	-0.82	0.25
18506	*Pax4*	paired box gene 4	0.41	0.44	-0.14	-0.11
18507	*Pax5*	paired box gene 5	-0.13	1.18	0.34	-0.71
18509	*Pax7*	paired box gene 7	-0.13	-0.38	-1.05	-0.11
18510	*Pax8*	paired box gene 8	-0.76	-0.24	-2.09	-1.39
18511	*Pax9*	paired box gene 9	0.03	-0.47	0.32	0.97
18514	*Pbx1*	pre B-cell leukemia transcription factor 1	-0.48	0.60	-0.69	1.06
18515	*Pbx2*	pre B-cell leukemia transcription factor 2	0.08	0.22	0.08	0.57
18516	*Pbx3*	pre B-cell leukemia transcription factor 3	-0.22	0.14	-0.91	0.12
80720	*Pbx4*	pre-B-cell leukemia homeobox 4	0.06	-0.27	0.31	0.06
71041	*Pcgf6*	polycomb group ring finger 6	-0.03	-0.26	-0.73	-0.41
18609	*Pdx1*	pancreatic and duodenal homeobox 1	-0.22	-0.14	0.74	-0.36
18667	*Pgr*	progesterone receptor	-1.08	-0.25	-1.63	-1.09
68479	*Phf5a*	PHD finger protein 5A	-0.24	-0.34	-0.71	-0.53
11859	*Phox2a*	paired-like homeobox 2a	0.24	-0.02	-0.42	-0.08
18935	*Phox2b*	paired-like homeobox 2b	0.41	1.44	-0.52	1.23
18740	*Pitx1*	paired-like homeodomain transcription factor 1	1.15	0.56	1.15	1.21
18742	*Pitx3*	paired-like homeodomain transcription factor 3	-0.40	0.41	-1.18	0.53

geneid	symbol	name	Day 5	Day 8	Day 11	Day 14
18771	*Pknox1*	Pbx/knotted 1 homeobox	0.12	0.30	-0.31	-0.09
208076	*Pknox2*	Pbx/knotted 1 homeobox 2	0.19	-0.19	1.53	0.97
18736	*Pou1f1*	POU domain, class 1, transcription factor 1	0.78	-0.14	1.15	-0.08
18986	*Pou2f1*	POU domain, class 2, transcription factor 1	-0.43	-0.27	-0.46	-0.74
18987	*Pou2f2*	POU domain, class 2, transcription factor 2	-0.98	-0.61	-0.89	-0.99
18988	*Pou2f3*	POU domain, class 2, transcription factor 3	-0.98	0.45	0.52	-0.01
18991	*Pou3f1*	POU domain, class 3, transcription factor 1	-1.45	-1.66	-3.59	-2.10
18992	*Pou3f2*	POU domain, class 3, transcription factor 2	-0.24	0.20	-0.37	-0.21
18993	*Pou3f3*	POU domain, class 3, transcription factor 3	0.64	0.38	-0.26	0.82
18994	*Pou3f4*	POU domain, class 3, transcription factor 4	-0.68	-1.22	-0.40	0.91
18996	*Pou4f1*	POU domain, class 4, transcription factor 1	0.65	0.59	0.29	0.36
18997	*Pou4f2*	POU domain, class 4, transcription factor 2	-1.55	-1.38	-0.35	-1.15
18998	*Pou4f3*	POU domain, class 4, transcription factor 3	-0.52	-1.46	-2.17	-0.76
75507	*Pou5f2*	POU domain class 5, transcription factor 2	-0.81	-0.51	0.86	-0.52
19009	*Pou6f1*	POU domain, class 6, transcription factor 1	-0.32	0.11	0.30	0.31
218030	*Pou6f2*	POU domain, class 6, transcription factor 2	-0.59	1.07	-0.73	-0.86
19013	*Ppara*	peroxisome proliferator activated receptor alpha	0.12	-0.09	1.19	0.56
19015	*Ppard*	peroxisome proliferator activator receptor delta	0.21	0.50	0.27	0.67
19016	*Pparg*	peroxisome proliferator activated receptor gamma	0.64	2.10	1.33	2.85
50907	*Preb*	prolactin regulatory element binding	-0.03	0.07	-0.11	0.31
19127	*Prop1*	paired like homeodomain factor 1	0.41	-0.14	-0.14	-0.11
20204	*Prrx2*	paired related homeobox 2	0.49	0.61	2.37	1.20
107751	*Prrxl1*	paired related homeobox protein-like 1	-0.89	0.44	-0.14	-0.71
19290	*Pura*	purine rich element binding protein A	0.44	0.14	0.31	-0.02
19291	*Purb*	purine rich element binding protein B	-0.42	-0.20	-0.10	-0.36
19401	*Rara*	retinoic acid receptor, alpha	0.06	-0.15	-0.79	-0.16
19434	*Rax*	retina and anterior neural fold homeobox	0.13	0.10	-0.28	0.52
19668	*Rbpjl*	recombination signal binding protein for immunoglobulin kappa J region-like	0.15	-0.82	0.56	-0.08
104383	*Rcor2*	REST corepressor 2	0.11	-0.14	-0.32	-0.01
19696	*Rel*	reticuloendotheliosis oncogene	-0.25	0.61	0.33	0.47
19698	*Relb*	avian reticuloendotheliosis viral (v-rel) oncogene related B	0.41	0.00	1.28	0.02
68703	*Rere*	arginine glutamic acid dipeptide (RE) repeats	-0.07	0.30	0.10	0.31
19883	*Rora*	RAR-related orphan receptor alpha	0.49	0.66	0.14	0.38
225998	*Rorb*	RAR-related orphan receptor beta	-0.22	0.44	0.53	-0.23
19885	*Rorc*	RAR-related orphan receptor gamma	-1.17	0.27	-2.33	-1.80
12395	*Runx1t1*	runt-related transcription factor 1; translocated to, 1 (cyclin D-related)	-0.86	-0.15	-1.37	-0.81
12399	*Runx3*	runt related transcription factor 3	-0.23	0.30	-0.05	-0.26
20181	*Rxra*	retinoid X receptor alpha	0.27	0.62	0.45	1.06
20182	*Rxrb*	retinoid X receptor beta	0.05	0.17	-0.24	0.12

geneid	symbol	name	Day 5	Day 8	Day 11	Day 14
20183	*Rxrg*	retinoid X receptor gamma	-2.15	-0.77	-3.11	-2.01
20230	*Satb1*	special AT-rich sequence binding protein 1	-0.76	0.06	-2.09	-0.10
212712	*Satb2*	special AT-rich sequence binding protein 2	0.71	0.49	0.56	0.37
18292	*Sebox*	SEBOX homeobox	-0.71	-1.37	-0.59	-1.52
20375	*Sfpi1*	SFFV proviral integration 1	-0.02	0.95	0.15	0.82
20464	*Sim1*	single-minded homolog 1 (Drosophila)	-0.45	0.24	-0.35	-0.61
20465	*Sim2*	single-minded homolog 2 (Drosophila)	0.15	0.92	1.65	1.07
20466	*Sin3a*	transcriptional regulator, SIN3A (yeast)	-0.02	0.07	-0.41	0.01
20471	*Six1*	sine oculis-related homeobox 1 homolog (Drosophila)	0.42	0.07	-0.49	0.16
20472	*Six2*	sine oculis-related homeobox 2 homolog (Drosophila)	0.00	-0.12	0.09	-0.10
20473	*Six3*	sine oculis-related homeobox 3 homolog (Drosophila)	0.20	0.70	0.38	-0.62
20474	*Six4*	sine oculis-related homeobox 4 homolog (Drosophila)	0.07	-0.24	-0.59	-0.37
20475	*Six5*	sine oculis-related homeobox 5 homolog (Drosophila)	-0.36	0.33	0.97	0.99
20476	*Six6*	sine oculis-related homeobox 6 homolog (Drosophila)	-1.18	0.75	-1.25	-1.15
17125	*Smad1*	MAD homolog 1 (Drosophila)	0.18	0.16	0.37	0.17
17126	*Smad2*	MAD homolog 2 (Drosophila)	0.26	0.21	0.14	0.14
17129	*Smad5*	MAD homolog 5 (Drosophila)	-0.29	0.17	0.31	0.45
17130	*Smad6*	MAD homolog 6 (Drosophila)	0.56	0.58	-0.04	1.41
17131	*Smad7*	MAD homolog 7 (Drosophila)	0.03	0.04	0.15	-0.06
55994	*Smad9*	MAD homolog 9 (Drosophila)	-0.72	0.29	0.29	0.19
30927	*Snai3*	snail homolog 3 (Drosophila)	-0.18	0.01	0.07	0.46
227631	*Sohlh1*	spermatogenesis and oogenesis specific basic helix-loop-helix 1	0.70	1.22	-0.69	0.86
20666	*Sox11*	SRY-box containing gene 11	-0.37	-0.67	-0.35	-0.47
223227	*Sox21*	SRY-box containing gene 21	1.43	-0.13	0.48	0.30
20683	*Sp1*	trans-acting transcription factor 1	-0.06	-0.03	-0.37	-0.30
20687	*Sp3*	trans-acting transcription factor 3	0.20	0.36	-0.12	0.29
20688	*Sp4*	trans-acting transcription factor 4	-0.10	0.31	-0.57	-0.01
30051	*Spdef*	SAM pointed domain containing ets transcription factor	0.69	0.22	-0.55	-0.11
56381	*Spen*	SPEN homolog, transcriptional regulator (Drosophila)	-0.12	0.26	0.19	0.11
272382	*Spib*	Spi-B transcription factor (Spi-1/PU.1 related)	0.50	-0.05	-0.12	-0.44
20728	*Spic*	Spi-C transcription factor (Spi-1/PU.1 related)	-0.34	0.71	1.56	0.59
79401	*Spz1*	spermatogenic leucine zipper 1	1.41	1.88	-0.06	0.45
20787	*Srebf1*	sterol regulatory element binding transcription factor 1	-0.38	0.30	0.31	0.29
20788	*Srebf2*	sterol regulatory element binding factor 2	-0.37	0.02	-0.18	-0.08
20807	*Srf*	serum response factor	-0.18	-0.09	0.12	-0.22

geneid	symbol	name	Day 5	Day 8	Day 11	Day 14
269397	*Ss18l1*	synovial sarcoma translocation gene on chromosome 18-like 1	0.37	0.18	0.49	0.15
240690	*St18*	suppression of tumorigenicity 18	0.81	0.33	-0.41	-0.37
20846	*Stat1*	signal transducer and activator of transcription 1	-0.18	0.50	0.45	0.88
20847	*Stat2*	signal transducer and activator of transcription 2	0.11	0.67	0.12	0.84
20849	*Stat4*	signal transducer and activator of transcription 4	-0.72	0.00	-0.34	-0.35
20851	*Stat5b*	signal transducer and activator of transcription 5B	0.30	0.43	1.37	0.67
20852	*Stat6*	signal transducer and activator of transcription 6	0.30	0.31	0.30	0.41
94186	*Strn3*	striatin, calmodulin binding protein 3	-0.08	-0.09	0.17	-0.08
226182	*Taf5*	TAF5 RNA polymerase II, TATA box binding protein (TBP)-associated factor	0.10	0.03	-0.35	-0.07
21343	*Taf6*	TAF6 RNA polymerase II, TATA box binding protein (TBP)-associated factor	0.04	0.09	-0.17	0.04
21374	*Tbp*	TATA box binding protein	-0.11	0.10	-0.35	0.09
227606	*Tbpl2*	TATA box binding protein like 2	0.41	-0.14	-0.14	-0.11
21375	*Tbr1*	T-box brain gene 1	0.07	0.15	0.41	-0.70
109575	*Tbx10*	T-box 10	1.13	1.43	-1.07	1.58
21384	*Tbx15*	T-box 15	0.59	0.10	1.03	0.64
83993	*Tbx19*	T-box 19	1.00	-1.12	-0.14	0.50
57765	*Tbx21*	T-box 21	-0.82	0.09	-0.78	0.45
245572	*Tbx22*	T-box 22	-1.52	-0.65	-1.75	-0.10
21387	*Tbx4*	T-box 4	0.27	0.53	-0.97	0.23
21389	*Tbx6*	T-box 6	0.39	0.43	0.02	-0.10
56070	*Tcerg1*	transcription elongation regulator 1 (CA150)	-0.03	-0.03	-0.40	-0.04
21406	*Tcf12*	transcription factor 12	-0.07	0.13	0.30	0.16
21411	*Tcf20*	transcription factor 20	0.12	0.39	0.12	0.33
21412	*Tcf21*	transcription factor 21	0.16	2.21	-0.78	-0.54
66855	*Tcf25*	transcription factor 25 (basic helix-loop-helix)	-0.04	0.06	0.00	0.08
21423	*Tcf3*	transcription factor 3	0.00	0.05	-0.33	0.11
21413	*Tcf4*	transcription factor 4	-0.50	0.19	-0.12	-0.08
21418	*Tcfap2a*	transcription factor AP-2, alpha	-0.09	-0.07	0.59	0.13
21419	*Tcfap2b*	transcription factor AP-2 beta	-0.15	0.20	0.40	0.05
21420	*Tcfap2c*	transcription factor AP-2, gamma	1.19	1.07	1.24	1.48
226896	*Tcfap2d*	transcription factor AP-2, delta	0.41	-0.14	-0.66	-0.11
332937	*Tcfap2e*	transcription factor AP-2, epsilon	-0.32	0.02	-0.94	0.18
209446	*Tcfe3*	transcription factor E3	-0.09	-0.13	-0.74	-0.40
21425	*Tcfeb*	transcription factor EB	0.82	0.46	0.83	0.71
21677	*Tead2*	TEA domain family member 2	-0.29	-0.02	-0.18	0.09
21678	*Tead3*	TEA domain family member 3	-0.37	0.32	1.44	0.99
21685	*Tef*	thyrotroph embryonic factor	0.48	0.06	-0.01	0.25
21780	*Tfam*	transcription factor A, mitochondrial	-0.07	-0.03	-0.31	-0.18
21781	*Tfdp1*	transcription factor Dp 1	-0.20	-0.17	-0.47	-0.29

geneid	symbol	name	Day 5	Day 8	Day 11	Day 14
211586	*Tfdp2*	transcription factor Dp 2	-0.36	0.05	-1.04	-0.11
54723	*Tfip11*	tuftelin interacting protein 11	0.06	0.11	-0.19	0.07
21815	*Tgif1*	TGFB-induced factor homeobox 1	-0.03	0.02	-0.17	0.11
228839	*Tgif2*	TGFB-induced factor homeobox 2	-0.11	0.17	-0.53	0.16
21833	*Thra*	thyroid hormone receptor alpha	0.15	-0.10	0.09	0.27
21834	*Thrb*	thyroid hormone receptor beta	0.31	1.23	0.16	0.71
21908	*Tlx1*	T-cell leukemia, homeobox 1	1.28	0.57	-1.23	1.39
21909	*Tlx2*	T-cell leukemia, homeobox 2	-0.20	1.21	-0.15	0.65
27140	*Tlx3*	T-cell leukemia, homeobox 3	0.09	0.45	0.44	0.29
21973	*Top2a*	topoisomerase (DNA) II alpha	-0.03	0.11	-0.74	-0.07
21974	*Top2b*	topoisomerase (DNA) II beta	-0.13	0.12	0.20	0.18
224829	*Trerf1*	transcriptional regulating factor 1	-0.17	-0.05	-0.80	0.00
21849	*Trim28*	tripartite motif-containing 28	0.12	-0.18	-0.58	-0.34
22061	*Trp63*	transformation related protein 63	1.31	-0.38	3.54	1.27
22062	*Trp73*	transformation related protein 73	-0.71	-1.21	-1.45	-1.54
83925	*Trps1*	trichorhinophalangeal syndrome I (human)	-0.11	-0.26	0.20	-0.42
21807	*Tsc22d1*	TSC22 domain family, member 1	0.52	0.14	1.06	-0.08
14605	*Tsc22d3*	TSC22 domain family, member 3	0.50	1.26	0.35	1.17
78829	*Tsc22d4*	TSC22 domain family, member 4	0.01	0.18	0.60	0.40
110796	*Tshz1*	teashirt zinc finger family member 1	-0.17	0.63	1.74	0.80
228911	*Tshz2*	teashirt zinc finger family member 2	-0.18	0.72	1.30	0.74
243931	*Tshz3*	teashirt zinc finger family member 3	0.23	0.05	-1.17	0.13
22160	*Twist1*	twist homolog 1 (Drosophila)	-0.32	0.29	-0.92	0.19
13345	*Twist2*	twist homolog 2 (Drosophila)	-0.68	0.43	1.42	2.04
22255	*Uncx*	UNC homeobox	-0.17	0.24	-0.27	-0.93
22278	*Usf1*	upstream transcription factor 1	-0.40	-0.25	-1.19	-0.51
22282	*Usf2*	upstream transcription factor 2	0.26	0.07	-0.02	0.11
22326	*Vax1*	ventral anterior homeobox containing gene 1	1.00	0.11	0.22	-0.11
24113	*Vax2*	ventral anterior homeobox containing gene 2	0.45	0.37	2.09	1.05
22337	*Vdr*	vitamin D receptor	0.45	0.01	2.00	1.17
21427	*Vps72*	vacuolar protein sorting 72 (yeast)	0.17	0.08	-0.05	0.12
114889	*Vsx1*	visual system homeobox 1 homolog (zebrafish)	-0.59	-0.13	2.12	0.32
12677	*Vsx2*	visual system homeobox 2	-0.13	1.07	-2.15	0.47
22433	*Xbp1*	X-box binding protein 1	-0.02	0.15	0.39	0.10
22632	*Yy1*	YY1 transcription factor	0.05	0.13	-0.08	0.09
235320	*Zbtb16*	zinc finger and BTB domain containing 16	0.04	0.74	0.72	1.41
22642	*Zbtb17*	zinc finger and BTB domain containing 17	0.09	0.01	0.16	0.21
268294	*Zbtb24*	zinc finger and BTB domain containing 24	0.24	0.27	0.39	0.28
245007	*Zbtb38*	zinc finger and BTB domain containing 38	0.42	0.19	0.39	0.12
22724	*Zbtb7b*	zinc finger and BTB domain containing 7B	0.17	0.05	0.79	0.41

geneid	symbol	name	Day 5	Day 8	Day 11	Day 14
57432	*Zc3h8*	zinc finger CCCH type containing 8	-0.31	-0.18	-0.53	-0.55
21417	*Zeb1*	zinc finger E-box binding homeobox 1	0.71	0.35	0.20	0.18
24136	*Zeb2*	zinc finger E-box binding homeobox 2	-0.37	-0.06	0.96	-0.19
11906	*Zfhx3*	zinc finger homeobox 3	-0.15	-0.12	0.36	-0.20
80892	*Zfhx4*	zinc finger homeodomain 4	-1.16	-0.31	0.15	-0.68
22646	*Zfp105*	zinc finger protein 105	-0.08	-0.28	-0.92	-0.31
22661	*Zfp148*	zinc finger protein 148	-0.08	0.19	0.14	0.17
385674	*Zfp174*	zinc finger protein 174	0.11	0.28	0.63	0.44
432731	*Zfp187*	zinc finger protein 187	-0.10	0.01	-0.01	-0.03
59057	*Zfp191*	zinc finger protein 191	-0.08	-0.17	-0.40	-0.14
69890	*Zfp219*	zinc finger protein 219	-0.08	0.04	-0.23	0.03
226442	*Zfp281*	zinc finger protein 281	0.02	-0.38	-0.30	-0.45
170740	*Zfp287*	zinc finger protein 287	-0.05	-0.01	-0.06	-0.26
21408	*Zfp354a*	zinc finger protein 354A	-0.44	0.05	-0.38	0.18
140482	*Zfp358*	zinc finger protein 358	-0.01	0.02	0.48	0.71
238673	*Zfp367*	zinc finger protein 367	0.01	-0.05	-0.35	-0.13
235682	*Zfp445*	zinc finger protein 445	-0.46	-0.12	-0.30	-0.17
232816	*Zfp628*	zinc finger protein 628	-0.11	0.26	-0.43	0.32
67778	*Zfp639*	zinc finger protein 639	0.01	-0.02	-0.21	-0.03
100009600	*Zglp1*	zinc finger, GATA-like protein 1	-0.37	-0.19	-0.49	-0.30
229007	*Zgpat*	zinc finger, CCCH-type with G patch domain	0.02	-0.12	0.14	-0.07
22770	*Zhx1*	zinc fingers and homeoboxes 1	-0.11	-0.01	0.69	0.07
387609	*Zhx2*	zinc fingers and homeoboxes 2	-0.24	-0.10	0.89	-0.10
320799	*Zhx3*	zinc fingers and homeoboxes 3	0.02	0.35	1.05	0.46
22771	*Zic1*	zinc finger protein of the cerebellum 1	-0.08	0.40	-1.16	-1.37
22772	*Zic2*	zinc finger protein of the cerebellum 2	-1.37	-0.28	-0.98	-0.10
65100	*Zic5*	zinc finger protein of the cerebellum 5	-1.04	-0.36	-1.09	-0.36
74570	*Zkscan1*	zinc finger with KRAB and SCAN domains 1	0.08	0.32	0.25	0.31
67235	*Zkscan14*	zinc finger with KRAB and SCAN domains 14	0.15	0.57	0.49	0.54
268417	*Zkscan17*	zinc finger with KRAB and SCAN domains 17	0.07	0.13	-0.30	-0.02
72739	*Zkscan3*	zinc finger with KRAB and SCAN domains 3	-0.38	-0.06	0.53	0.08
22757	*Zkscan5*	zinc finger with KRAB and SCAN domains 5	0.06	0.21	-0.47	0.00
52712	*Zkscan6*	zinc finger with KRAB and SCAN domains 6	0.28	0.28	0.18	0.29
332221	*Zscan10*	zinc finger and SCAN domain containing 10	-0.48	-0.85	-1.18	-0.97
22758	*Zscan12*	zinc finger and SCAN domain containing 12	0.15	0.08	0.10	-0.07
22691	*Zscan2*	zinc finger and SCAN domain containing 2	0.32	0.44	0.61	0.61
269585	*Zscan20*	zinc finger and SCAN domains 20	-0.11	0.08	-0.23	0.06
22697	*Zscan21*	zinc finger and SCAN domain containing 21	0.03	0.08	-0.15	0.14
80292	*Zxdc*	ZXD family zinc finger C	0.27	0.86	0.38	1.01

Supplemental Table S6. Expression levels of transcription factors that regulate cardiomyocyte differentiation in TCDD-treated AHR-positive cells relative to controls (Log2 Fold change)

**geneid**	**symbol**	**AHR PWM**	**name**	**Day 5**	**Day 8**	**Day 11**	**Day 14**
11910	*Atf3*	No	activating transcription factor 3	0.37	2.51	0.44	0.22
223922	*Atf7*	No	activating transcription factor 7	0.21	1.01	0.24	0.28
12053	*Bcl6*	Yes	B-cell leukemia/lymphoma 6	0.34	2.62	0.77	0.68
12578	*Cdkn2a*	No	cyclin-dependent kinase inhibitor 2A	0.54	1.78	0.41	0.22
12606	*Cebpa*	No	CCAAT/enhancer binding protein (C/EBP), alpha	-0.14	1.09	1.35	0.33
12705	*Cited1*	Yes	Cbp/p300-interacting transactivator with Glu/Asp-rich carboxy-terminal domain 1	0.08	2.06	1.84	1.35
17684	*Cited2*	No	Cbp/p300-interacting transactivator, with Glu/Asp-rich carboxy-terminal domain, 2	0.08	1.35	0.81	0.79
12914	*Crebbp*	Yes	CREB binding protein	0.02	0.06	-0.03	0.19
12387	*Ctnnb1*	No	catenin (cadherin associated protein), beta 1	0.00	-0.16	0.14	0.08
13393	*Dlx3*	Yes	distal-less homeobox 3	0.48	2.99	1.61	1.04
13819	*Epas1*	Yes	endothelial PAS domain protein 1	0.43	0.17	-0.06	0.11
23871	*Ets1*	Yes	E26 avian leukemia oncogene 1, 5' domain	0.45	1.79	0.00	-0.06
15376	*Foxa2*	No	forkhead box A2	-0.50	0.91	0.21	-0.10
17300	*Foxc1*	No	forkhead box C1	0.53	1.28	1.48	0.26
14234	*Foxc2*	No	forkhead box C2	1.81	1.14	2.21	1.64
14106	*Foxh1*	No	forkhead box H1	-0.72	-1.01	-1.25	-0.82
108655	*Foxp1*	No	forkhead box P1	-0.89	-1.11	-0.90	-0.53
14461	*Gata2*	Yes	GATA binding protein 2	0.73	1.66	2.09	1.41
14462	*Gata3*	Yes	GATA binding protein 3	1.34	2.28	1.47	0.69
14463	*Gata4*	No	GATA binding protein 4	0.59	0.84	0.69	0.19
14465	*Gata6*	No	GATA binding protein 6	0.90	1.91	0.94	0.43
14472	*Gbx2*	No	gastrulation brain homeobox 2	0.08	-0.20	-1.07	-0.94
14634	*Gli3*	Yes	GLI-Kruppel family member GLI3	-0.24	0.29	0.20	0.06
15110	*Hand1*	Yes	heart and neural crest derivatives expressed transcript 1	0.03	4.32	1.60	1.08
15111	*Hand2*	Yes	heart and neural crest derivatives expressed transcript 2	1.14	-0.86	1.55	1.37
433759	*Hdac1*	No	histone deacetylase 1	-0.07	-0.29	0.00	-0.01
15214	*Hey2*	No	hairy/enhancer-of-split related with YRPW motif 2	-0.33	0.57	0.30	0.58
21405	*Hnf1a*	No	HNF1 homeobox A	0.90	-0.25	0.13	0.22
74318	*Hopx*	No	HOP homeobox	-1.73	-1.46	-0.35	-0.42
15394	*Hoxa1*	Yes	homeobox A1	0.59	1.76	1.08	0.77
15395	*Hoxa10*	Yes	homeobox A10	1.22	1.19	0.44	0.93
15400	*Hoxa3*	No	homeobox A3	0.32	2.24	0.66	0.78

geneid	symbol	AHR PWM	name	Day 5	Day 8	Day 11	Day 14
15410	*Hoxb3*	No	homeobox B3	-1.18	-1.56	0.54	0.60
15413	*Hoxb5*	No	homeobox B5	0.61	-2.91	-0.54	0.87
15430	*Hoxd10*	No	homeobox D10	1.74	-0.31	0.06	0.68
50916	*Irx4*	No	Iroquois related homeobox 4 (Drosophila)	0.35	3.37	1.97	-0.01
16392	*Isl1*	No	ISL1 transcription factor, LIM/homeodomain	0.24	0.70	1.07	0.32
16476	*Jun*	No	Jun oncogene	0.46	2.16	0.24	0.20
16598	*Klf2*	No	Kruppel-like factor 2 (lung)	1.06	0.65	0.60	0.38
12224	*Klf5*	Yes	Kruppel-like factor 5	0.32	1.53	0.28	0.11
16842	*Lef1*	Yes	lymphoid enhancer binding factor 1	-2.34	-2.59	-0.86	-0.25
17260	*Mef2c*	No	myocyte enhancer factor 2C	-1.39	-2.19	0.23	0.47
17268	*Meis1*	No	Meis homeobox 1	0.33	2.85	1.28	0.56
17292	*Mesp1*	No	mesoderm posterior 1	-1.05	-0.37	0.39	0.08
17293	*Mesp2*	No	mesoderm posterior 2	1.21	-0.46	0.44	0.69
17387	*Mmp14*	Yes	matrix metallopeptidase 14 (membrane-inserted)	-0.32	2.52	0.15	-0.15
17701	*Msx1*	Yes	homeobox, msh-like 1	-2.06	-2.26	-0.48	0.77
17869	*Myc*	Yes	myelocytomatosis oncogene	-0.83	-0.54	-0.58	-0.31
18109	*Mycn*	Yes	v-myc myelocytomatosis viral related oncogene, neuroblastoma derived (avian)	-0.41	-1.39	-1.01	-0.61
71950	*Nanog*	Yes	Nanog homeobox	0.90	0.40	0.73	0.61
18091	*Nkx2-5*	No	NK2 transcription factor related, locus 5 (Drosophila)	-1.15	-0.78	-8901.00	-0.93
18092	*Nkx2-6*	No	NK2 transcription factor related, locus 6 (Drosophila)	-1.15	-0.78	-0.11	-0.14
11819	*Nr2f2*	Yes	nuclear receptor subfamily 2, group F, member 2	2.06	3.14	0.83	0.05
15370	*Nr4a1*	No	nuclear receptor subfamily 4, group A, member 1	-0.08	1.13	0.37	0.01
26423	*Nr5a1*	Yes	nuclear receptor subfamily 5, group A, member 1	-0.05	0.17	0.93	-0.62
26424	*Nr5a2*	Yes	nuclear receptor subfamily 5, group A, member 2	1.18	1.28	0.38	0.21
18505	*Pax3*	Yes	paired box gene 3	-0.21	1.36	-0.68	-0.23
18508	*Pax6*	No	paired box gene 6	-0.54	-0.77	-0.50	-0.26
18741	*Pitx2*	Yes	paired-like homeodomain transcription factor 2	-0.41	-1.07	0.05	0.30
18999	*Pou5f1*	Yes	POU domain, class 5, transcription factor 1	-0.57	-0.79	-0.32	-0.18
19130	*Prox1*	Yes	prospero-related homeobox 1	-0.01	0.83	0.14	0.28
18933	*Prrx1*	No	paired related homeobox 1	-0.05	1.22	-0.05	0.29
218772	*Rarb*	No	retinoic acid receptor, beta	0.71	2.73	1.59	0.42
19411	*Rarg*	Yes	retinoic acid receptor, gamma	-0.23	-0.47	-0.38	-0.16
19697	*Rela*	No	v-rel reticuloendotheliosis viral oncogene homolog A (avian)	0.24	1.21	0.13	0.08
12394	*Runx1*	Yes	runt related transcription factor 1	-0.05	2.53	0.04	-0.19
12393	*Runx2*	Yes	runt related transcription factor 2	-0.04	-0.44	-1.21	-0.35

geneid	symbol	AHR PWM	name	Day 5	Day 8	Day 11	Day 14
20429	*Shox2*	No	short stature homeobox 2	0.07	0.60	1.46	0.69
17127	*Smad3*	No	MAD homolog 3 (Drosophila)	0.20	1.30	0.65	0.21
17128	*Smad4*	Yes	MAD homolog 4 (Drosophila)	0.22	0.84	-0.04	0.00
20665	*Sox10*	Yes	SRY-box containing gene 10	-0.04	-1.41	0.15	0.00
20671	*Sox17*	Yes	SRY-box containing gene 17	1.11	1.08	0.89	0.14
20672	*Sox18*	No	SRY-box containing gene 18	-0.26	-2.47	-0.11	0.17
20674	*Sox2*	Yes	SRY-box containing gene 2	0.59	-0.25	0.10	0.12
20677	*Sox4*	Yes	SRY-box containing gene 4	-0.42	-0.12	-0.19	-0.09
20678	*Sox5*	Yes	SRY-box containing gene 5	0.93	0.26	1.09	-0.17
20679	*Sox6*	No	SRY-box containing gene 6	-0.19	1.04	-0.36	-0.45
20680	*Sox7*	Yes	SRY-box containing gene 7	0.88	0.13	-0.42	0.11
20681	*Sox8*	No	SRY-box containing gene 8	0.68	-0.72	0.17	0.68
20682	*Sox9*	Yes	SRY-box containing gene 9	0.09	0.94	1.38	-0.23
20848	*Stat3*	No	signal transducer and activator of transcription 3	0.35	1.25	0.37	0.27
20850	*Stat5a*	Yes	signal transducer and activator of transcription 5A	0.72	1.35	0.95	0.52
20997	*T*	Yes	brachyury	-3.14	-0.35	-0.29	-1.26
21349	*Tal1*	No	T-cell acute lymphocytic leukemia 1	-1.75	-3.80	-1.96	1.09
21380	*Tbx1*	No	T-box 1	0.89	0.78	0.39	-0.17
76365	*Tbx18*	Yes	T-box18	1.05	-0.29	0.22	0.35
21385	*Tbx2*	No	T-box 2	0.26	0.83	1.13	0.74
57246	*Tbx20*	No	T-box 20	-0.40	0.25	-0.45	0.29
21386	*Tbx3*	Yes	T-box 3	1.59	2.22	1.29	0.84
21388	*Tbx5*	Yes	T-box 5	0.48	-0.12	-0.46	-0.09
21414	*Tcf7*	No	transcription factor 7, T-cell specific	-0.19	-0.84	-0.10	-0.03
21415	*Tcf7l1*	No	transcription factor 7-like 1 (T-cell specific, HMG box)	0.00	-0.51	0.17	0.02
21416	*Tcf7l2*	No	transcription factor 7-like 2, T-cell specific, HMG-box	-0.09	0.61	0.70	0.22
21676	*Tead1*	No	TEA domain family member 1	0.06	1.30	0.25	0.07
21679	*Tead4*	Yes	TEA domain family member 4	0.43	2.29	0.46	0.19
22059	*Trp53*	Yes	transformation related protein 53	0.05	-0.26	-0.18	-0.09
22431	*Wt1*	No	Wilms tumor 1 homolog	-1.24	-3.08	-1.15	-1.06
22773	*Zic3*	Yes	zinc finger protein of the cerebellum 3	-0.23	-0.43	-0.62	-0.39

Supplemental Table S7. Summary of PcG and TxG genes affected in their expression by TCDD treatment in AHR-positive cells

Gene	Description	Function	Expression	Reference
Cbx1	Chromobox protein homolog 1	Component of heterochromatin.	In all adult and embryonic tissues	(Singh et al. 1991)
Cbx2	Chromobox protein homolog 2; also known as M33.	Component of a PcG multiprotein PRC1-like complex, which function as a transcriptional repressor. Critical roles in germ cell viability, meiosis onset and homologous chromosome synapsis in mammalian germ line.	Expressed in most embryonic tissue except the heart from ED8.5-11.5; expressed in CNS, PNS, olfactory and tongue epithelia, lung gastrointestinal duct and urogenital system at ED 13.5; CNS, thymus, various epithelial cell types at ED 15.5.	(Schoorlemmer et al. 1997; Baumann and De La Fuente 2011)
Cbx3	Chromobox protein homolog 3	Component of heterochromatin; interact with Dia-1/SRF, whose interaction is essential for smooth muscle cell differentiation from stem cells and for the development of functional cardiovascular system.	Localized in the nucleus; associated with euchromatin and is largely excluded from constitutive heterochromatin.	(Horsley et al. 1996; Xiao et al. 2011)
Cbx4	Chromobox protein homolog 4	E3 SUMO-protein ligase which facilitates SUMO1 conjugation; a component of a PcG multiprotein PRC1-like complex.	Localized in the nucleus.	(Alkema et al. 1997; Ismail et al. 2012)
Cbx8	Chromobox protein homolog 8	Component of a PcG multiprotein PRC1-like complex, which functions as a transcriptional repressor.	Expressed at low levels throughout embryogenesis, decreased at later stages.	(Hemenway et al. 2000; Cao et al. 2005)
Chd3	Chromodomain helicase DNA binding protein 3	Not known in mouse. In human, it is a component of the histone deacetylase NuRD complex which remodels chromatin by deacetylating histones.	Widely expressed in human. Not known in mouse.	(Tong et al. 1998)
Chd9	Chromodomain-helicase-DNA-binding protein 9;peroxisomal proliferator-activator receptor A-interacting complex 320 kDa protein	May act as a transcriptional co-activator for PPARA and possibly other nuclear receptor; a potential ATP-dependent chromatin remodeling protein; binds to A/T-rich DNA and has DNA-dependent ATPase activity	Expressed in osteoprogenitor cells during development and in mature bone.	(Shur et al. 2006; Surapureddi et al. 2006)
Dnmt3a	DNA methyltransferase	Required for genome-wide *de novo* methylation and is essential for the establishment of DNA methylation patterns during development. Also plays a role in paternal and maternal imprinting; essential for mammalian proper development. Regulate gene transcription in addition to *de novo* methylation.	Ubiquitously expressed in mesenchymal cells after ED 10.5 during embryogenesis. In somatic cells, isoform 1 is expressed ubiquitously at low levels. Isoform 2 is restricted to tissues containing cells which are undergoing active de novo methylation.	(Okano et al. 1999; Fuks et al. 2001; Watanabe et al. 2002)

Gene	Description	Function	Expression	Reference
Dnmt3b	DNA methyltransferase	Required for genome-wide *de novo* methylation and is essential for the establishment of DNA methylation patterns during development. Also function as transcriptional co-repressor.	Highly expressed in totipotent embryonic cells such as inner cell mass, epiblast and embryonic ectoderm cells.	(Okano et al. 1999; Watanabe et al. 2002)
Mbd5	Belong to Methyl-Binding proteins	Associate with heterochromatin but do not bind to methylated DNA. May contribute to the formation or function of heterochromatin.	Expressed in all tissues, but with a wide range of levels; the highest expression was in the brain, testis and oocytes.	(Laget et al. 2010)
Mll5	Myeloid/lymphoid or mixed-lineage leukemia protein 5 homolog	Specifically mono-and di-methylates Histone H3 Lys4. The methylated H3K4 represents a specific tag for epigenetic transcriptional activation. Also function as an important cell cycle regulator.	Nucleus speckle (by similarity)	(Sebastian et al. 2009)
Pcgf5	Polycomb group RING finger protein 5	Component of a PcG multiprotein PRC-1 like complex, which function as a transcriptional repressor.	May expressed in nucleus (by similarity)	(Barrett et al. 2009)
Pcgf6	Polycomb group RING finger protein 6	Transcriptional repressor. A component of a PcG multiprotein PRC-1 like complex, which functions as a transcriptional repressor.	Expressed in ovary, testis, stomach, liver, thymus and kidney.	(Akasaka et al. 2002)
Ph1	Polyhomeotic-like protein 1	Component of a PcG multiprotein PRC1-like complex, which functions as transcription repressor of many genes throughout development.	Highly expressed in testis with lower levels in most other tissues	(Takihara et al. 1997; Isono et al. 2005)
Ph2	Polyhomeotic-like protein 2	Refer to the above cell	Ubiquitously expressed in embryos and adult tissues.	(Yamaki et al. 2002; Isono et al. 2005)
Setbp1	SET-binding protein	May function in the SET-related leukemogenesis and tumorigenesis by suppressing SET function	Expressed ubiquitously in all human adult tissues.	(Minakuchi et al. 2001)
Setd7	Histone H4-K4 methyltransferase SETD7; SET domain-containing protein.	Specifically monomethylates Histone H3 Lys4. Methylated H4K4 represents a marker for epigenetic transcriptional activation.	Expressed during all pre-implementation stages during embryogenesis.	(Jeong et al. 2005)
Sirt2	Silent information regulator 2, members of Sir2 family share a ~260 amino acid region of homology, being divided into five classes.	NAD-dependent deacetylase. SIRT2 is an important regulator of programmed necrosis. Marked protection from ischaemic injury was observed from either Sirt2$^{-/-}$ mice or wild type mice treated with Sirt2 inhibitor.	Expressed in the cytoplasma, colocalized with microtubules.	(Narayan et al. 2012)
Smarca1	SWI/SNF-related matrix-associated actin-dependent regulator of chromatin subfamily A member 1	Energy-transducing component of the nucleosome-remodeling factor and CERF complex. May play a role in neural development.	Predominantly expressed in cortex, cerebellum, ovaries, testes, uterus and placenta. During development, expressed throughout the embryo at ED 9.5 to ED 15.5	(Lazzaro and Picketts 2001; Barak et al. 2003)

Gene	Description	Function	Expression	Reference
Smarca2	A member of the SWI/SNF family of proteins.	Transcriptional coactivator cooperating with hormone receptors to potentiate transcription activation. Involved in vitamin D-coupled transcription regulation. Belong to the neural progenitors-specific chromatin remodeling complex and the neuron-specific chromatin remodeling complex, with the former essential for the self-renewal/proliferative capacity of the neural stem cells and the later role of regulating the genes for dendrite growth. Mutations in this gene cause Nicolaides-Baraitser syndrome in human.	Expressed in the cortical plate in the embryo and in the cortex and the hippocampus in adult. Localized in the nucleus.	(Lessard et al. 2007; Van Houdt et al. 2012)
Suv39h2	Histone H3-lysine 9 N-methyltransferase 2	Specifically trimethylates Histone H3 Lys9 using monomethylationed H3 Lys9 as substrate. H3-Lys9 trimethylation is a specific tag for epigenetics transcriptional repression. Epigenetically regulate telomere length in mammalian cells; may regulate higher-order chromatin organization;	Testis specific; predominant expressed in type B spermatogonia and preleptotene spermatocytes;	(O'Carroll et al. 2000; Peters et al. 2001; Garcia-Cao et al. 2004)
Suv39h1	Histon H3-lysine 9 N-methyltransferase 1	Refer to the above cell. In addition, Suv39h1 is targeted to Histone H3 via its interaction with RB1 and is involved in many processes including regulation of the control switch for exiting the cell cycle and entering differentiation etc.	Expression present throughout embryogenesis; widely expressed.	(Nielsen et al. 2001; Peters et al. 2001; Garcia-Cao et al. 2004)

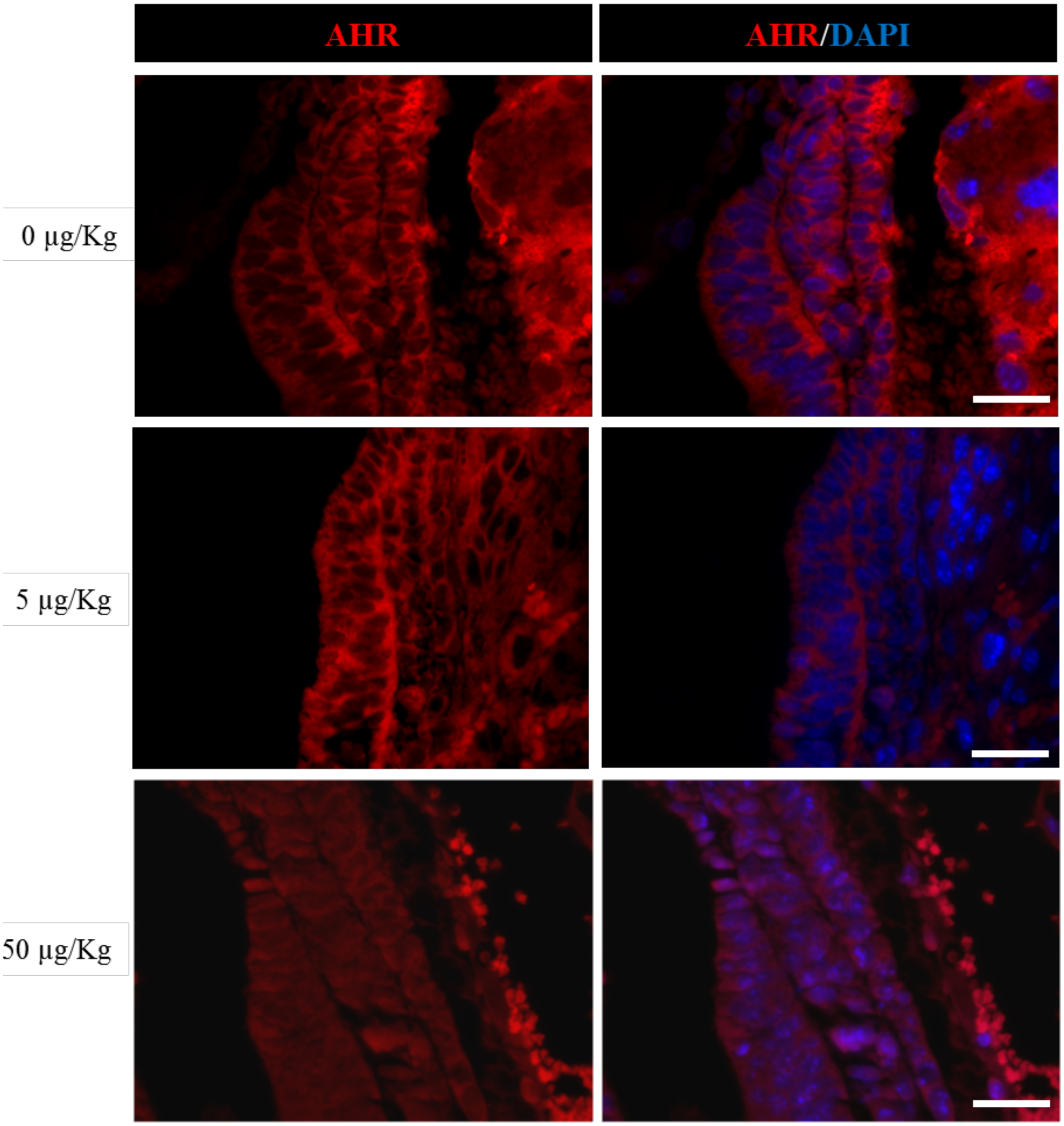

Supplemental Figure S1. Higher magnification of the GD7.5 embryos shown in Fig. 1A.

The micrograph shows the localization of the AHR in cytosol in control and at the low dose of 5 µg/kg and in both cytosol and nucleus at the higher dose of 50 µg/kg. Bar = 20 µm

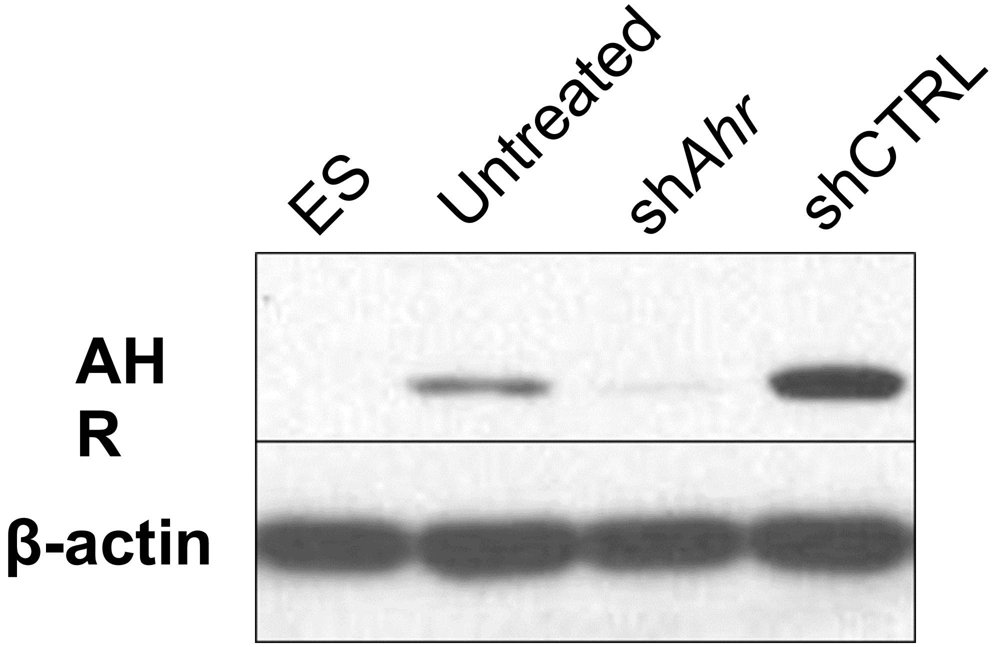

Supplemental Figure S2. Stable integration of an *Ahr* ShRNA lentivirus inhibits AHR expression.

ES cells were infected with a lentivirus control (ShCTRL) or a lentivirus expressing *Ahr* ShRNA (sh*Ahr*) and subjected to EB differentiation. Cell lysates were prepared on differentiation day 7 and used for Western immunoblotting using AHR antibodies. *ES*: ES cell lysate; *Untreated*: lysate from ES cells allowed to differentiate for 7 days.

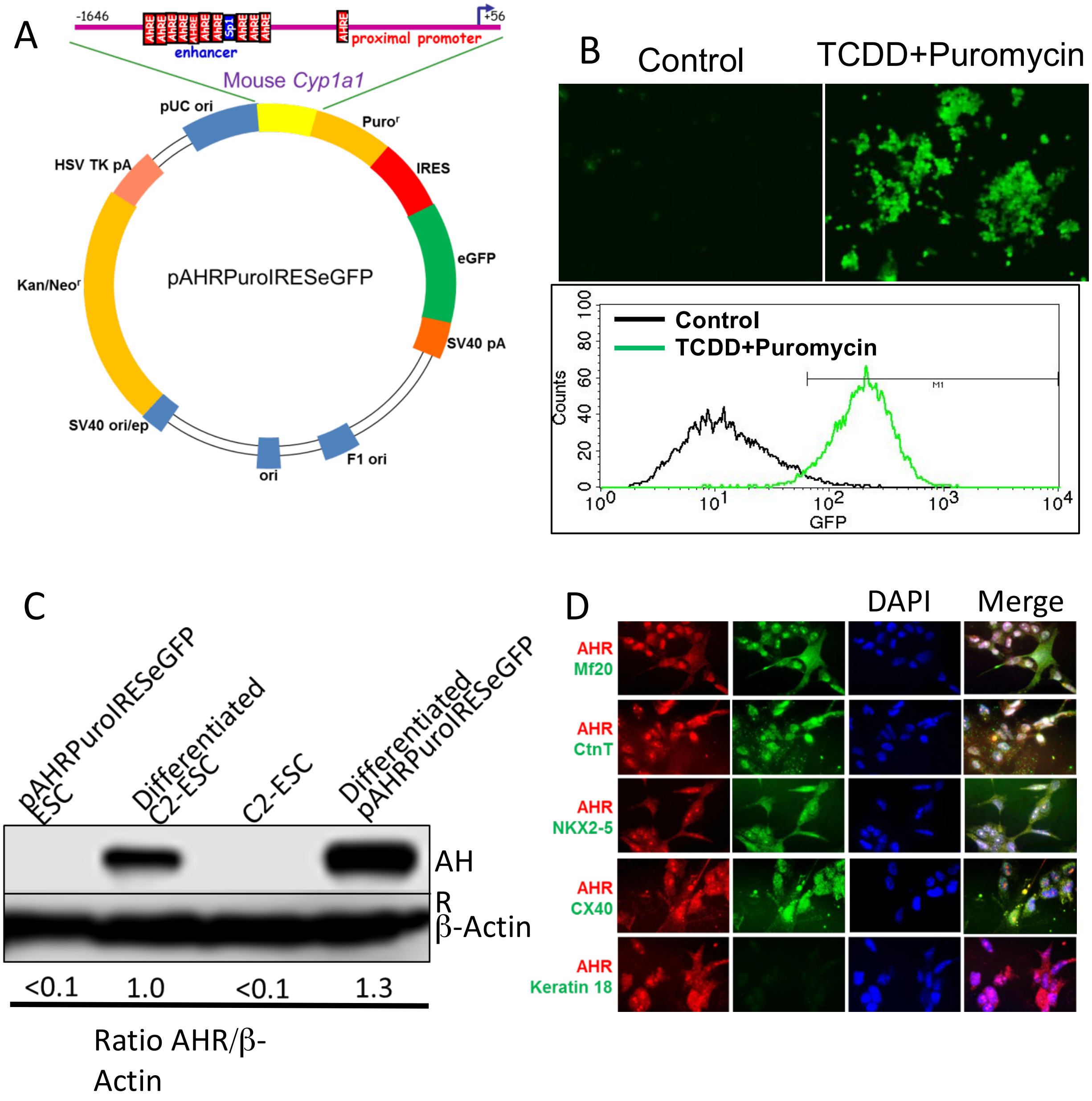

Supplemental Figure S3. Characterization of AHR-positive cardiomyocytes.

(A) Map of the pAHRPuroIRESeGFP vector used for promoter sorting cell selection. (B) EGFP fluorescence increased significantly in puromycin-resistant differentiating cells and the eGFP-expressing cells were detectable microscopically (Top panels). Fluorescence-activated cell sorting analysis demonstrated an obvious fluorescent shift (Bottom panel). We estimate the purity of these cells at greater than 90%. (C) Western blot immunodetection of AHR expression

in the pAHRPuroIRESeGFP cells. AHR expression is undetectable in both the undifferentiated pAHRPuroIRESeGFP ES cells and the parent C2-ES cells. After differentiation, the PAHRPuroIRESeGFP cells show some 30% increase in AHR levels relative to β-actin in comparison to the AHR levels in differentiated C2-ES cells, probably due to the selection against AHR-negative cells. (D) A pAHRPuroIRESeGFP clone, termed C5, was further expanded, allowed to differentiate for several passages and characterized for expression of developmental markers by immunofluorescence. All survivors of puromycin selection expressed AHR, the mesodermal marker MF20, and the mesendodermal marker GATA4, but lacked expression of the ectodermal marker keratin 18. DAPI: nuclear staining; Merge: merge of all three images in columns 1–3. These cells also expressed NKX2-5, a cardiac homeobox transcription factor and marker of mesoderm, and the cardiac markers connexin 40 and troponin T, strongly suggesting that the selected cells are cardiomycytes.

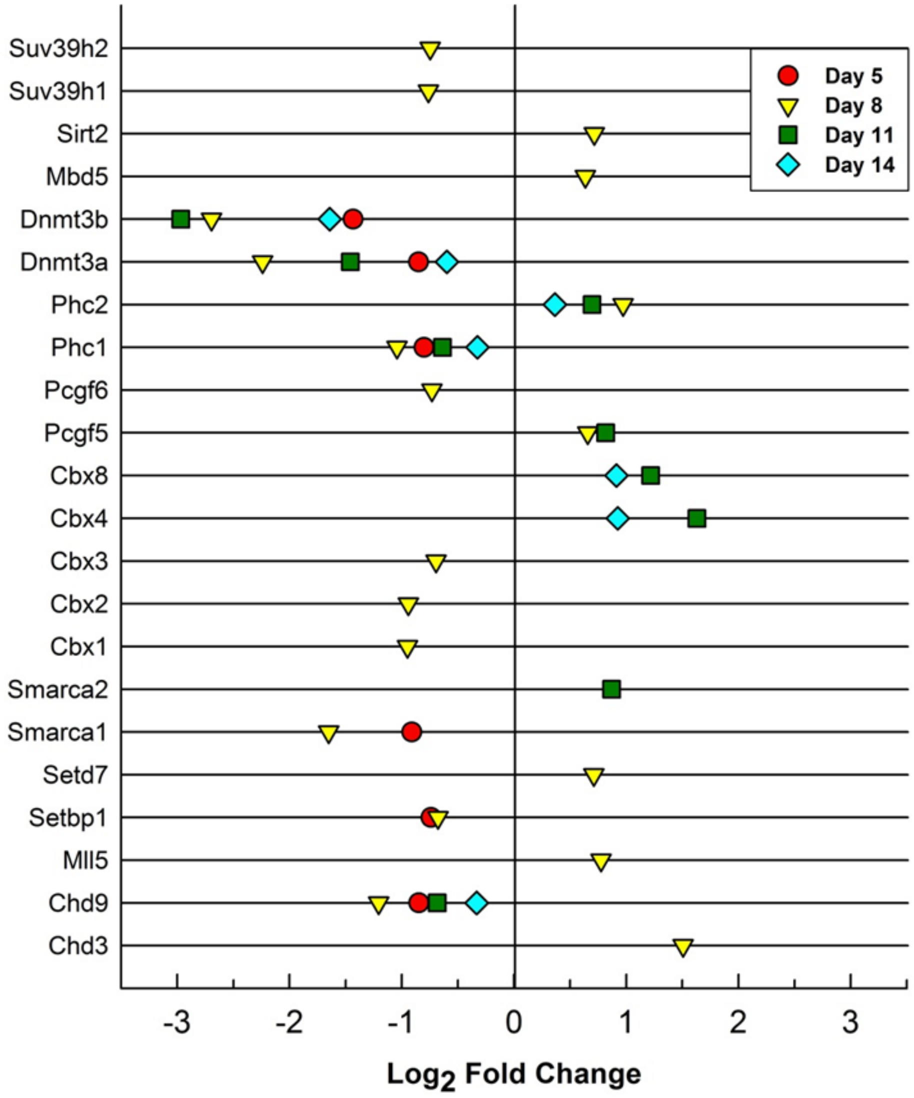

Supplemental Figure S4. Time-dependent expression changes of PcG and TxG genes deregulated by the AHR/TCDD axis.

PcG and TxG genes that show altered expression in AHR positive cells treated with TCDD.

References

Akasaka, T., N. Takahashi, et al. (2002). "MBLR, a new RING finger protein resembling mammalian Polycomb gene products, is regulated by cell cycle-dependent phosphorylation." Genes Cells **7**(8):835-850.

Alkema, M. J., J. Jacobs, et al. (1997). "MPc2, a new murine homolog of the Drosophila polycomb protein is a member of the mouse polycomb transcriptional repressor complex." J Mol Biol **273**(5):993-1003.

Anders S, Huber W. 2010. Differential expression analysis for sequence count data. Genome Biol 11:R106.

Barak, O., M. A. Lazzaro, et al. (2003). "Isolation of human NURF:a regulator of Engrailed gene expression." EMBO J **22**(22):6089-6100.

Barrett T, Troup DB, Wilhite SE, Ledoux P, Rudnev D, Evangelista C, et al. 2009. NCBI GEO:archive for high-throughput functional genomic data. Nucleic Acids Res 37:D885-D890.

Baumann, C. and R. De La Fuente (2011). "Role of Polycomb Group Protein Cbx2/M33 in Meiosis Onset and Maintenance of Chromosome Stability in the Mammalian Germline." Genes (Basel) **2**(1):59-80.

Biel, M., C. Wahl-Schott, et al. (2009). "Hyperpolarization-activated cation channels:from genes to function." Physiol Rev **89**(3):847-885.

Black, B. L. and E. N. Olson (1998). "Transcriptional control of muscle development by myocyte enhancer factor-2 (MEF2) proteins." Annu Rev Cell Dev Biol **14**:167-196.

Cao, R., Y. Tsukada, et al. (2005). "Role of Bmi-1 and Ring1A in H2A ubiquitylation and Hox gene silencing." Mol Cell **20**(6):845-854.

Chaldoupi, S. M., P. Loh, et al. (2009). "The role of connexin40 in atrial fibrillation." Cardiovasc Res **84**(1):15-23.

Durocher, D., F. Charron, et al. (1997). "The cardiac transcription factors Nkx2-5 and GATA-4 are mutual cofactors." EMBO J **16**(18):5687-5696.

El Sharaby, A. A., M. Egerbacher, et al. (2001). "Immunohistochemical demonstration of Leu-7 (HNK-1), Neurone-specific Enolase (NSE) and Protein-Gene Peptide (PGP) 9.5 in the developing camel (Camelus dromedarius) heart." Anat Histol Embryol **30**(6):321-325.

Espinoza-Lewis, R. A., L. Yu, et al. (2009). "Shox2 is essential for the differentiation of cardiac pacemaker cells by repressing Nkx2-5." Dev Biol **327**(2):376-385.

Franco, D., W. H. Lamers, et al. (1998). "Patterns of expression in the developing myocardium:towards a morphologically integrated transcriptional model." Cardiovasc Res **38**(1):25-53.

Fuks, F., W. A. Burgers, et al. (2001). "Dnmt3a binds deacetylases and is recruited by a sequence-specific repressor to silence transcription." EMBO J **20**(10):2536-2544.

Garcia-Cao, M., R. O'Sullivan, et al. (2004). "Epigenetic regulation of telomere length in mammalian cells by the Suv39h1 and Suv39h2 histone methyltransferases." Nat Genet **36**(1):94-99.

Gertsenstein M, Nutter LM, Reid T, Pereira M, Stanford WL, Rossant J, et al. 2010. Efficient generation of germ line transmitting chimeras from C57BL/6N ES cells by aggregation with outbred host embryos. PLoS One 5:e11260.

Greulich, F., C. Rudat, et al. (2011). "Mechanisms of T-box gene function in the developing heart." Cardiovasc Res **91**(2):212-222.

Harvey, R. P. (1996). "NK-2 homeobox genes and heart development." Dev Biol **178**(2):203-216.

Harzheim, D., K. H. Pfeiffer, et al. (2008). "Cardiac pacemaker function of HCN4 channels in mice is confined to embryonic development and requires cyclic AMP." EMBO J **27**(4):692-703.

Hemenway, C. S., B. W. Halligan, et al. (2000). "Identification and analysis of a third mouse Polycomb gene, MPc3." Gene **242**(1-2):31-40.

Horsley, D., A. Hutchings, et al. (1996). "M32, a murine homologue of Drosophila heterochromatin protein 1 (HP1), localises to euchromatin within interphase nuclei and is largely excluded from constitutive heterochromatin." Cytogenet Cell Genet **73**(4):308-311.

Houweling, A. C., M. M. van Borren, et al. (2005). "Expression and regulation of the atrial natriuretic factor encoding gene Nppa during development and disease." Cardiovasc Res **67**(4):583-593.

Ihaka P, Gentleman R. 1996. R:A language for data analysis and graphics. In: 299-314.

Ismail, I. H., J. P. Gagne, et al. (2012). "CBX4-mediated SUMO modification regulates BMI1 recruitment at sites of DNA damage." Nucleic Acids Res **40**(12):5497-5510.

Isono, K., Y. Fujimura, et al. (2005). "Mammalian polyhomeotic homologues Phc2 and Phc1 act in synergy to mediate polycomb repression of Hox genes." Mol Cell Biol **25**(15):6694-6706.

Jeong, K. S., J. H. Park, et al. (2005). "The analysis of X-chromosome inactivation-related gene expression from single mouse embryo with sex-determination." Biochem Biophys Res Commun **333**(3):803-807.

Kuruppu, S. and A. I. Smith (2012). "Endothelin Converting Enzyme-1 phosphorylation and trafficking." FEBS Lett **586**(16):2212-2217.

Laget, S., M. Joulie, et al. (2010). "The human proteins MBD5 and MBD6 associate with heterochromatin but they do not bind methylated DNA." PLoS One **5**(8):e11982.

Lazzaro, M. A. and D. J. Picketts (2001). "Cloning and characterization of the murine Imitation Switch (ISWI) genes:differential expression patterns suggest distinct developmental roles for Snf2h and Snf2l." J Neurochem **77**(4):1145-1156.

Lessard, J., J. I. Wu, et al. (2007). "An essential switch in subunit composition of a chromatin remodeling complex during neural development." Neuron **55**(2):201-215.

Lien, C. L., C. Wu, et al. (1999). "Control of early cardiac-specific transcription of Nkx2-5 by a GATA-dependent enhancer." Development **126**(1):75-84.

Minakuchi, M., N. Kakazu, et al. (2001). "Identification and characterization of SEB, a novel protein that binds to the acute undifferentiated leukemia-associated protein SET." Eur J Biochem **268**(5):1340-1351.

Morgan M, Anders S, Lawrence M, Aboyoun P, Pages H, Gentleman R. 2009. ShortRead:a bioconductor package for input, quality assessment and exploration of high-throughput sequence data. Bioinformatics 25:2607-2608.

Narayan, N., I. H. Lee, et al. (2012). "The NAD-dependent deacetylase SIRT2 is required for programmed necrosis." Nature **492**(7428):199-204.

O'Carroll, D., H. Scherthan, et al. (2000). "Isolation and characterization of Suv39h2, a second histone H3 methyltransferase gene that displays testis-specific expression." Mol Cell Biol **20**(24):9423-9433.

Okano, M., D. W. Bell, et al. (1999). "DNA methyltransferases Dnmt3a and Dnmt3b are essential for de novo methylation and mammalian development." Cell **99**(3):247-257.

Otsuki, T., K. Yata, et al. (2004). "Expression of protein gene product 9.5 (PGP9.5)/ubiquitin-C-terminal hydrolase 1 (UCHL-1) in human myeloma cells." Br J Haematol **127**(3):292-298.

Patient, R. K. and J. D. McGhee (2002). "The GATA family (vertebrates and invertebrates)." Curr Opin Genet Dev **12**(4):416-422.

Pereira, A. H., C. F. Clemente, et al. (2009). "MEF2C silencing attenuates load-induced left ventricular hypertrophy by modulating mTOR/S6K pathway in mice." PLoS One **4**(12):e8472.

Peters, A. H., D. O'Carroll, et al. (2001). "Loss of the Suv39h histone methyltransferases impairs mammalian heterochromatin and genome stability." Cell **107**(3):323-337.

Sartor MA, Leikauf GD, Medvedovic M. 2009. LRpath:a logistic regression approach for identifying enriched biological groups in gene expression data. Bioinformatics 25:211-217.

Schoorlemmer, J., C. Marcos-Gutierrez, et al. (1997). "Ring1A is a transcriptional repressor that interacts with the Polycomb-M33 protein and is expressed at rhombomere boundaries in the mouse hindbrain." EMBO J **16**(19):5930-5942.

Sebastian, S., P. Sreenivas, et al. (2009). "MLL5, a trithorax homolog, indirectly regulates H3K4 methylation, represses cyclin A2 expression, and promotes myogenic differentiation." Proc Natl Acad Sci U S A **106**(12):4719-4724.

Shinde K, Phatak M, Johannes FM, Chen J, Li Q, Vineet JK, et al. 2010. Genomics Portals:integrative web-platform for mining genomics data. BMC Genomics 11:27.

Shur, I., R. Socher, et al. (2006). "In vivo association of CReMM/CHD9 with promoters in osteogenic cells." J Cell Physiol **207**(2):374-378.

Singh, P. B., J. R. Miller, et al. (1991). "A sequence motif found in a Drosophila heterochromatin protein is conserved in animals and plants." Nucleic Acids Res **19**(4):789-794.

Sohl, G. and K. Willecke (2004). "Gap junctions and the connexin protein family." Cardiovasc Res **62**(2):228-232.

Stieber, J., S. Herrmann, et al. (2003). "The hyperpolarization-activated channel HCN4 is required for the generation of pacemaker action potentials in the embryonic heart." Proc Natl Acad Sci U S A **100**(25):15235-15240.

Surapureddi, S., N. Viswakarma, et al. (2006). "PRIC320, a transcription coactivator, isolated from peroxisome proliferator-binding protein complex." Biochem Biophys Res Commun **343**(2):535-543.

Takihara, Y., D. Tomotsune, et al. (1997). "Targeted disruption of the mouse homologue of the Drosophila polyhomeotic gene leads to altered anteroposterior patterning and neural crest defects." Development **124**(19):3673-3682.

Tong, J. K., C. A. Hassig, et al. (1998). "Chromatin deacetylation by an ATP-dependent nucleosome remodelling complex." Nature **395**(6705):917-921.

Trapnell C, Pachter L, Salzberg SL. 2009. TopHat:discovering splice junctions with RNA-Seq. Bioinformatics 25:1105-1111.

Van Houdt, J. K., B. A. Nowakowska, et al. (2012). "Heterozygous missense mutations in SMARCA2 cause Nicolaides-Baraitser syndrome." Nat Genet **44**(4):445-449, S441.

Wang, Y., Y. Zheng, et al. (2007). "Polymorphisms of KDR gene are associated with coronary heart disease." J Am Coll Cardiol **50**(8):760-767.

Watanabe, D., I. Suetake, et al. (2002). "Stage- and cell-specific expression of Dnmt3a and Dnmt3b during embryogenesis." Mech Dev **118**(1-2):187-190.

Weber H, Birnbaum LS. 1985. 2,3,7,8-Tetrachlorodibenzo-p-dioxin (TCDD) and 2,3,7,8-tetrachlorodibenzofuran (TCDF) in pregnant C57BL/6N mice:distribution to the embryo and excretion. Arch Toxicol 57:159-162.

Weiss, A. and L. A. Leinwand (1996). "The mammalian myosin heavy chain gene family." Annu Rev Cell Dev Biol **12**:417-439.

Xiao, Q., G. Wang, et al. (2011). "Chromobox protein homolog 3 is essential for stem cell differentiation to smooth muscles in vitro and in embryonic arteriogenesis." Arterioscler Thromb Vasc Biol **31**(8):1842-1852.

Yamaki, M., K. Isono, et al. (2002). "The mouse Edr2 (Mph2) gene has two forms of mRNA encoding 90- and 36-kDa polypeptides." Gene **288**(1-2):103-110.
